# Effects of azilsartan compared with telmisartan on insulin resistance in patients with essential hypertension and type 2 diabetes mellitus: An open-label, randomized clinical trial

**DOI:** 10.1371/journal.pone.0214727

**Published:** 2019-04-03

**Authors:** Mitsuhide Naruse, Yasuhiro Koike, Nozomu Kamei, Ryuichi Sakamoto, Yuko Yambe, Michinori Arimitsu

**Affiliations:** 1 Division of Endocrinology, Metabolism, and Hypertension, Clinical Research Institute for Endocrine and Metabolic Diseases, National Hospital Organization, Kyoto Medical Center, Kyoto, Japan; 2 Japan Medical Affairs, Takeda Pharmaceutical Company Limited, Tokyo, Japan; 3 Department of Endocrinology and Metabolism, Hiroshima Red Cross Hospital & Atomic-bomb Survivors Hospital, Hiroshima, Japan; 4 Department of Endocrinology and Metabolism, National Hospital Organization Kyushu Medical Center, Fukuoka, Japan; 5 Department of Endocrinology and Diabetes, National Hospital Organization Nagoya Medical Center, Nagoya, Japan; 6 Data Science Division, Biostatistics Department I, A2 Healthcare Corporation, Tokyo, Japan; University of Newcastle, AUSTRALIA

## Abstract

**Background:**

Based on non-clinical data, it is expected that azilsartan, an angiotensin II receptor blocker, will help improve insulin resistance in addition to its hypotensive action. The present study is aimed to explore the effect of azilsartan compared to telmisartan on insulin sensitivity in hypertensive patients in the clinical setting.

**Methods:**

This multicenter, randomized, open-label, parallel-group exploratory study was conducted in Japan. We randomized adult patients (≥20 years old) with grade I or II essential hypertension and coexisting type 2 diabetes (1:1) to receive either oral azilsartan (20 mg/day;17 patients) or telmisartan (40 mg/day;16 patients) for 12 weeks. The primary endpoint was the change in the homeostasis model assessment ratio of insulin resistance (HOMA-R) from the baseline at the end of the treatment period. We also evaluated its safety and efficacy on other diabetes-related variables and blood pressure.

**Findings:**

The mean changes in HOMA-R at the end of treatment were 0.22 (95% CI, −1.09–1.52) in the azilsartan group and −0.23 (95% CI, −0.72–0.27) in the telmisartan group. We found no clinically remarkable changes between the groups in diabetes-related variables such as fasting blood glucose, fasting insulin, HbA1c (NGSP), HOMA-β, or 1,5-anhydroglucitol. Reductions in clinic systolic and diastolic blood pressure were observed at week 4 and the reduced levels were maintained throughout the treatment period in both groups. No serious treatment-emergent adverse events (TEAEs) were observed. Only one drug-related TEAE (mild decrease in blood pressure) was reported in one patient in the azilsartan group.

**Conclusion:**

Neither azilsartan nor telmisartan had any clinically remarkable effects on insulin resistance parameters when administered for 12 weeks to patients with grade I or II essential hypertension and coexisting type 2 diabetes mellitus. Azilsartan (20 mg/day) and telmisartan (40 mg/day) exerted comparable antihypertensive effects.

**Trial registration:**

ClinicalTrials.gov NCT02079805

## Introduction

The Guidelines for the Management of Hypertension issued by the Japanese Society of Hypertension state that hypertension and type 2 diabetes mellitus are primary risk factors for major vascular disorders caused by endothelial dysfunction, atherothrombosis, and so on [1]. Since the incidence of cerebrovascular disease and/or ischemic heart disease increases in the concurrent presence of hypertension and type 2 diabetes mellitus, strict management of blood pressure and blood glucose levels is recommended in hypertensive patients with type 2 diabetes mellitus [1, 2].

Hypertension and type 2 diabetes mellitus represent components of metabolic syndrome, a disorder based on insulin resistance [2, 3]. Therefore, when selecting antihypertensive agents for hypertensive patients with type 2 diabetes mellitus, not only the antihypertensive effect but also effects on insulin sensitivity, glucose metabolism, and lipid metabolism should be considered. Thus, the Guidelines for the Management of Hypertension recommend angiotensin II receptor blockers (ARBs) and angiotensin converting enzyme (ACE) inhibitors, which have been shown to improve insulin sensitivity, as the first-line drugs for patients with concurrent hypertension and type 2 diabetes mellitus without adversely affecting lipid metabolism [1]. Telmisartan, an ARB with strong antihypertensive efficacy, has been shown to activate peroxisome proliferator-activated receptor γ (PPARγ) in non-clinical studies and to improve diabetes-related indices, including insulin resistance, in hypertensive patients with type 2 diabetes mellitus [3–10]. PPARγ activation is believed to promote adiponectin production by adipose cells, which in turn enhances insulin sensitivity [11].

Azilsartan is a relatively new ARB that has considerable clinical hypotensive action compared with other ARBs [12, 13]. Non-clinical studies have shown that azilsartan increases the expression of PPARγ and decreases that of tumor necrosis factor-α (TNF-α), a cytokine that reduces insulin sensitivity; therefore, it is expected to improve insulin resistance in clinical settings [14–16]. However, no clinical data comparing the effects of azilsartan and other ARBs on insulin resistance are available.

Thus, we planned to explore the effects of azilsartan compared with telmisartan on insulin resistance by measuring the homeostasis model assessment ratio of insulin resistance (HOMA-R) and other diabetes-related variables in patients with concurrent grade I or II essential hypertension and type 2 diabetes mellitus.

## Materials and methods

### Study design and ethics statement

This multicenter, randomized, open-label, parallel-group exploratory study was conducted at 27 centers in Japan between June 2014 and April 2016. The study protocol was reviewed and approved by the Institutional Review Boards of all 27 centers, and the study was conducted in compliance with all Institutional Review Board regulations and in accordance with the principles of the Declaration of Helsinki and the Ethical Guidelines for Clinical Research (Ministry of Health, Labour, and Welfare of Japan). The trial was registered at ClinicalTrials.gov (identification number, NCT02079805). Informed consent was obtained from all patients included in the study.

### Patients

We assessed patients for eligibility after obtaining their informed consent. The inclusion criteria were as follows: (a) outpatients aged ≥20 years, (b) presence of grade I or II essential hypertension (sitting systolic blood pressure ≥130 mmHg and <180 mmHg or sitting diastolic blood pressure ≥80 mmHg and <110 mmHg at the start of the treatment period), (c) presence of coexisting type 2 diabetes with an HbA1c level of <8.4% (National Glycohemoglobin Standardization Program [NGSP]) and a ≤0.3% change in HbA1c (peak minus nadir during three months before the start of the treatment period), and (d) fixed diet/exercise therapy, if applicable, for three months before the start of the treatment period. The exclusion criteria were as follows: (a) grade III essential hypertension (i.e., sitting systolic blood pressure ≥180 mmHg or sitting diastolic blood pressure ≥110 mmHg), secondary hypertension, or malignant hypertension; (b) use of oral antihypertensive medication within two weeks before the start of the treatment period; (c) grade II essential hypertension treated with antihypertensive drugs (sitting systolic blood pressure ≥160 mmHg or sitting diastolic blood pressure ≥100 mmHg); (d) use of renin–angiotensin system (RAS) inhibitors or thiazolidines within three months before the start of the treatment period; (e) type 1 diabetes mellitus; (f) fasting blood glucose ≥180 mg/dL and HOMA-R ≤1.6 at the start of the treatment period (Week 0); (g) receiving or requiring treatment with insulin, glucagon-like peptide (GLP)-1 receptor agonists, or other parenteral hypoglycemic agents, or combination therapy with three or more oral hypoglycemic agents; (h) antidiabetic medication changes (including dosage and administration changes) within three months before the start of the treatment period; (i) having been diagnosed with myocardial infarction, cerebral infarction, cerebral hemorrhage, or transient ischemic attack, or having undergone a coronary revascularization procedure within three months before the start of the treatment period, or presenting with unstable conditions after the start of the treatment period; (j) having been diagnosed with or treated for advanced hypertensive retinopathy within three months before the start of treatment; (k) presenting with severe ketosis, diabetic coma (or precoma), severe infection, serious trauma, clinically evident renal disorder (e.g., eGFR <30 mL/min/1.73 m^2^), markedly low bile secretion, or severe hepatic disorder; (l) having a history of hyper-sensitivity or allergy to azilsartan or telmisartan, or both; and (m) being pregnant, possibly pregnant, or breast-feeding.

### Randomization and interventions

Eligible patients were randomized to the azilsartan or telmisartan groups at a ratio of 1:1 considering baseline HOMA-R (<2.5 or ≥2.5) and concurrent use of biguanides (yes or no) as stratification factors. The registration center performed the randomization, and the related information was not disclosed to the sponsors/investigators until the end of the study.

The patients orally received azilsartan (20 mg/day) or telmisartan (40 mg/day) in the morning before or after breakfast. On days of scheduled site visits, we administered the study drugs after tests/examinations. Concomitant use of drugs affecting the HOMA-R or blood pressure, including thiazolidines, insulin, GLP-1 receptor agonists, and all antihypertensives, was prohibited during the study. We also prohibited the concomitant use of oral hypoglycemic agents other than those that had been taken at the time of obtaining informed consent. We allowed the continuous use of other drugs used to treat concurrent diseases. However, we did not allow changes in the dosage and administration of oral hypoglycemic agents from those used at the time of obtaining informed consent. We required participating patients using antihypertensives at the time of obtaining informed consent to undergo a two-week washout period before the start of treatment.

### Endpoints

We chose the change in HOMA-R from baseline at the end of the treatment period as the primary efficacy endpoint. HOMA-R was calculated according to the formula: fasting insulin (μU/mL) × fasting glucose (mg/dL) / 405. The secondary efficacy endpoints were changes in the following variables: fasting blood glucose, fasting insulin, HbA1c (NGSP), HOMA-β, and 1, 5-anhydroglucitol (AG). HOMA-β was calculated according to the formula: fasting insulin (μU/mL) × 360 / (fasting glucose [mg/dL] − 63). We also assessed changes in clinic and home blood pressure levels.

We monitored adverse events (AEs) during the study and evaluated their severity and causal relationship with the study drugs; we used the system organ class and preferred term to report AEs coded according to the Medical Dictionary for Regulatory Activities (MedDRA) version 19.0.

### Statistical analysis

We analyzed the efficacy endpoints using the full analysis set (FAS), which comprised patients who were randomized and received at least one dose of the tested drugs during the study period. For the primary endpoint, we calculated descriptive statistics of HOMA-R for each treatment group on the FAS population as the primary analysis. As a secondary analysis of the primary endpoint, we also examined the change in HOMA-R for subgroups stratified using the baseline HOMA-R values (<2.5 or ≥2.5) and concurrent use of biguanides (Yes or No). We analyzed other efficacy endpoints using the FAS population. We performed safety analysis using the safety analysis set (SAS), which comprised patients who received at least one dose of the test medication during the study period.

Since this was an exploratory study, we did not perform statistical significance tests on each variable.

We had originally planned to include 50 patients in each treatment group in consideration of feasibility, not based on a statistical rationale. However, we reduced that number to 20 in each group due to the slow enrollment of patients meeting the stringent eligibility criteria and the limited study period.

## Results

### Study population

**Fig 1:**
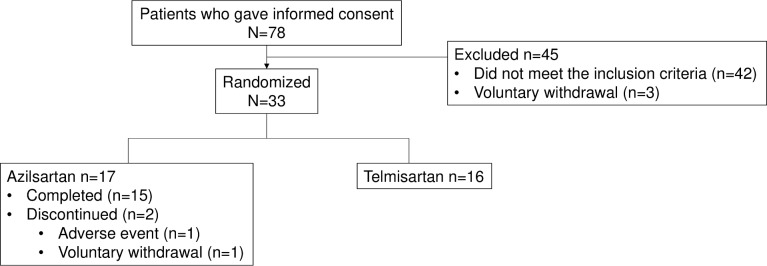
CONSORT diagram of participant recruitment.

Of the 78 patients who provided informed consent, we excluded 45 who did not meet the inclusion criteria or who met the exclusion criteria (n = 42) or who withdrew voluntarily (n = 3). Thus, we randomly assigned 33 patients to either one of the two treatment groups (17, azilsartan group; 16, telmisartan group) and included them in the FAS and SAS populations (Fig 1). Of these, 31 (15, azilsartan group; 16, telmisartan group) completed the study, and 2 in the azilsartan group prematurely discontinued the drug due to an AE and voluntary withdrawal (1 each). The mean (SD) treatment durations were 80.5 days (±19.37) in the azilsartan group and 87.6 days ((4.65) in the telmisartan group.

The baseline characteristics in the FAS were comparable between the two groups (Table 1).

**Table 1:** Demographic and baseline characteristics of patients (FAS).

		Azilsartan	Telmisartan	Total
		20 mg	40 mg	
Variable		(n = 17)	(n = 16)	(n = 33)
Age (years)				
	Mean (SD)	63.2 (12.76)	65.3 (9.10)	64.2 (11.02)
	Range	43–82	49–84	43–84
Sex (n[%])				
	Male	7 (41.2)	7 (43.8)	14 (42.4)
	Female	10 (58.8)	9 (56.3)	19 (57.6)
Body weight (kg)				
	Mean (SD)	70.4 (14.88)	71.0 (16.69)	70.7 (15.53)
	Range	43.8–102.0	47.5–112.4	43.8–112.4
Height (cm)				
	Mean (SD)	160.6 (11.19)	160.6 (11.13)	160.6 (10.99)
	Range	143–178	144–183	143–183
Body Mass Index (kg/m^2^)				
	Mean (SD)	27.2 (4.64)	27.2 (3.72)	27.2 (4.15)
	Range	19.5–35.3	21.4–37.6	19.5–37.6
Duration of hypertension (years)				
	Mean (SD)	3.5 (4.39)	4.7 (4.39)	4.1 (4.36)
	Range	0–15.5	0–16.3	0–16.3
Duration of diabetes mellitus (years)				
	Mean (SD)	4.9 (5.04)	4.5 (4.28)	4.7 (4.62)
	Range	0.3–13.8	0–16.1	0–16.1
Concurrent use of biguanides (n[%])				
	Presence	4 (23.5)	3 (18.8)	7 (21.2)
	Absence	13 (76.5)	13 (81.3)	26 (78.8)
Clinic SBP (mmHg)				
	Mean (SD)	143.3 (9.28)	145.6 (9.91)	144.4 (9.51)
	Range	131–162	126–165	126–165
Clinic DBP (mmHg)				
	Mean (SD)	88.8 (7.19)	89.3 (10.61)	89.0 (8.87)
	Range	75–101	72–108	72–108
Morning SBP (home; mmHg)				
	Mean (SD)	147.3 (11.95)	140.1 (14.62)	143.5 (13.66)
	Range	126.2–173.4	117.0–159.9	117.0–173.4
Morning DBP (home; mmHg)				
	Mean (SD)	88.4 (8.95)	85.5 (9.28)	86.9 (9.08)
	Range	74.3–104.4	73.5–104.2	73.5–104.4
Before bedtime SBP (mmHg)				
	Mean (SD)	145.1 (12.43)	141.4 (13.90)	143.1 (13.14)
	Range	119.9–165.8	115.9–162.6	115.9–165.8
Before bedtime DBP (mmHg)				
	Mean (SD)	84.5 (7.68)	82.9 (8.61)	83.6 (8.09)
	Range	72.7–97.3	69.2–98.8	69.2–98.8

FAS, full analysis set; SD, standard deviation; HOMA-R, homeostasis model assessment ratio of insulin resistance; SBP, systolic blood pressure; DBP, diastolic blood pressure.

### Efficacy results

The mean (SD) of baseline HOMA-R was 4.24 (±1.843) in the azilsartan group and 3.31 (±1.366) in the telmisartan group. The mean [95% confidence interval (CI)] changes in HOMA-R from the baseline at the end of treatment were 0.22 (−1.09–1.52) in the azilsartan group and −0.23 (−0.72–0.27) in the telmisartan group (Table 2). The mean difference in the changes from the baseline in HOMA-R between the azilsartan and telmisartan groups was 0.44 (−0.89–1.78).

**Table 2:** Changes from the baseline values in HOMA-R at the end of the treatment period (FAS).

	Azilsartan 20 mg		Telmisartan 40 mg		Total		Azilsartan − Telmisartan^a^
	(n = 17)		(n = 16)		(n = 33)		
	Observed value at visit	Change from baseline	Observed value at visit	Change from baseline	Observed value at visit	Change from baseline	Change from baseline
Baseline							
n	17		16		33		
Mean (SD)	4.24 (1.843)		3.31 (1.366)		3.79 (1.671)		
End of treatment							
n	16	16	16	16	32	32	
Mean (SD)	4.34 (2.031)	0.22 (2.449)	3.09 (1.403)	−0.23 (0.928)	3.72 (1.832)	0.00 (1.835)	0.44
[95% CI]		[−1.09, 1.52]		[−0.72, 0.27]		[−0.66, 0.66]	[−0.89, 1.78]

FAS, full analysis set; SD, standard deviation; HOMA-R, homeostasis model assessment ratio of insulin resistance; 95% CI, 95% confidence interval [lower, upper].

^a^The mean difference in the changes from baseline between the azilsartan and telmisartan groups

Patient populations were stratified by subgroups based on the baseline HOMA-R values of <2.5 or ≥2.5 and with or without concurrent use of biguanides. No clinically remarkable differences were observed in the changes in HOMA-R from baseline in the subgroups with HOMA-R values of ≥2.5 and without concurrent use of biguanides. The number of the patients in the subgroups with HOMA-R values <2.5 and concurrent use of biguanides was too small to be evaluated (Table 3).

**Table 3:** Changes from the baseline values in HOMA-R at the end of the treatment period by subgroup (FAS).

		Azilsartan 20 mg		Telmisartan 40 mg		Total	
Subgroup		(n = 17)		(n = 16)		(n = 33)	
		Observed value at visit	Change from baseline	Observed value at visit	Change from baseline	Observed value at visit	Change from baseline
Baseline HOMA-R <2.5							
	Baseline						
	n	4		3		7	
	Mean (SD)	2.05 (0.058)		1.87 (0.306)		1.97 (0.206)	
	End of treatment						
	n	4	4	3	3	7	7
	Mean (SD)	2.73 (1.595)	0.68 (1.640)	1.80 (0.608)	−0.07 (0.651)	2.33 (1.280)	0.36 (1.282)
Baseline HOMA-R ≥2.5							
	Baseline						
	n	13		13		26	
	Mean (SD)	4.91 (1.564)		3.65 (1.293)		4.28 (1.546)	
	End of treatment						
	n	12	12	13	13	25	25
	Mean (SD)	4.88 (1.914)	0.07 (2.710)	3.38 (1.375)	−0.26 (0.999)	4.10 (1.791)	-0.10 (1.973)
Concurrent use of biguanides Yes							
	Baseline						
	n	4		3		7	
	Mean (SD)	3.28 (1.634)		3.77 (1.504)		3.49 (1.469)	
	End of treatment						
	n	4	4	3	3	7	7
	Mean (SD)	3.48 (1.357)	0.20 (2.309)	3.37 (1.137)	−0.40 (0.436)	3.43 (1.164)	-0.06 (1.683)
Concurrent use of biguanides No							
	Baseline						
	n	13		13		26	
	Mean (SD)	4.53 (1.860)		3.21 (1.375)		3.87 (1.739)	
	End of treatment						
	n	12	12	13	13	25	25
	Mean (SD)	4.63 (2.181)	0.23 (2.593)	3.02 (1.491)	−0.18 (1.017)	3.80 (1.991)	0.01 (1.908)

FAS, full analysis set; SD, standard deviation; HOMA-R, homeostasis model assessment ratio of insulin resistance.

We also found no clinically remarkable effects of azilsartan or telmisartan on the other diabetes-related variables (Table 4).

**Table 4:** Changes from the baseline values in other diabetes-related variables at the end of the treatment period (FAS).

		Azilsartan 20 mg		Telmisartan 40 mg		Total	
Variable		(n = 17)		(n = 16)		(n = 33)	
		Observed value at visit	Change from baseline	Observed value at visit	Change from baseline	Observed value at visit	Change from baseline
Fasting blood glucose (mg/dL)							
	Baseline						
	n	17		16		33	
	Mean (SD)	131.53 (19.539)		125.44 (20.468)		128.58 (19.920)	
	End of treatment						
	n	16	16	16	16	32	32
	Mean (SD)	132.13 (20.232)	2.00 (18.308)	124.38 (21.450)	−1.06 (14.991)	128.25 (20.885)	0.47 (16.533)
	[95% CI]		[−7.76, 11.76]		[−9.05, 6.93]		[−5.49, 6.43]
Fasting insulin (μU/mL)							
	Baseline						
	n	17		16		33	
	Mean (SD)	13.006 (5.4046)		10.808 (4.0803)		11.940 (4.8635)	
	End of treatment						
	n	16	16	16	16	32	32
	Mean (SD)	13.306 (6.2115)	0.475 (6.3847)	9.990 (4.5035)	−0.818 (2.7623)	11.648 (5.5965)	-0.171 (4.8834)
	[95% CI]		[−2.927, 3.877]		[−2.289, 0.654]		[−1.932, 1.589]
HbA1c (NGSP) (%)							
	Baseline						
	n	17		16		33	
	Mean (SD)	6.81 (0.488)		6.63 (0.411)		6.72 (0.454)	
	End of treatment						
	n	17	17	16	16	33	33
	Mean (SD)	6.89 (0.360)	0.09 (0.382)	6.73 (0.471)	0.10 (0.290)	6.82 (0.419)	0.09 (0.335)
	[95% CI]		[−0.11, 0.28]		[−0.05, 0.25]		[−0.02, 0.21]
HOMA-β (%)							
	Baseline						
	n	17		16		33	
	Mean (SD)	73.28 (34.302)		69.30 (32.681)		71.35 (33.061)	
	End of treatment						
	n	16	16	16	16	32	32
	Mean (SD)	73.51 (39.200)	−0.44 (30.985)	65.42 (36.544)	−3.88 (20.151)	69.47 (37.505)	-2.16 (25.770)
	[95% CI]		[−16.95, 16.07]		[−14.62, 6.86]		[−11.45, 7.13]
1, 5-AG (μg/mL)							
	Baseline						
	n	17		16		33	
	Mean (SD)	13.10 (6.893)		13.06 (7.095)		13.08 (6.881)	
	End of treatment						
	n	16	16	16	16	32	32
	Mean (SD)	12.26 (6.815)	−0.66 (2.454)	13.31 (8.069)	0.24 (2.143)	12.78 (7.366)	-0.21 (2.312)
	[95% CI]		[−1.96, 0.65]		[−0.90, 1.39]		[−1.04, 0.63]

FAS, full analysis set; SD, standard deviation; 95% CI, 95% confidence interval [lower, upper]; 1, 5-AG, 1, 5-anhydroglucitol.

**Fig 2:**
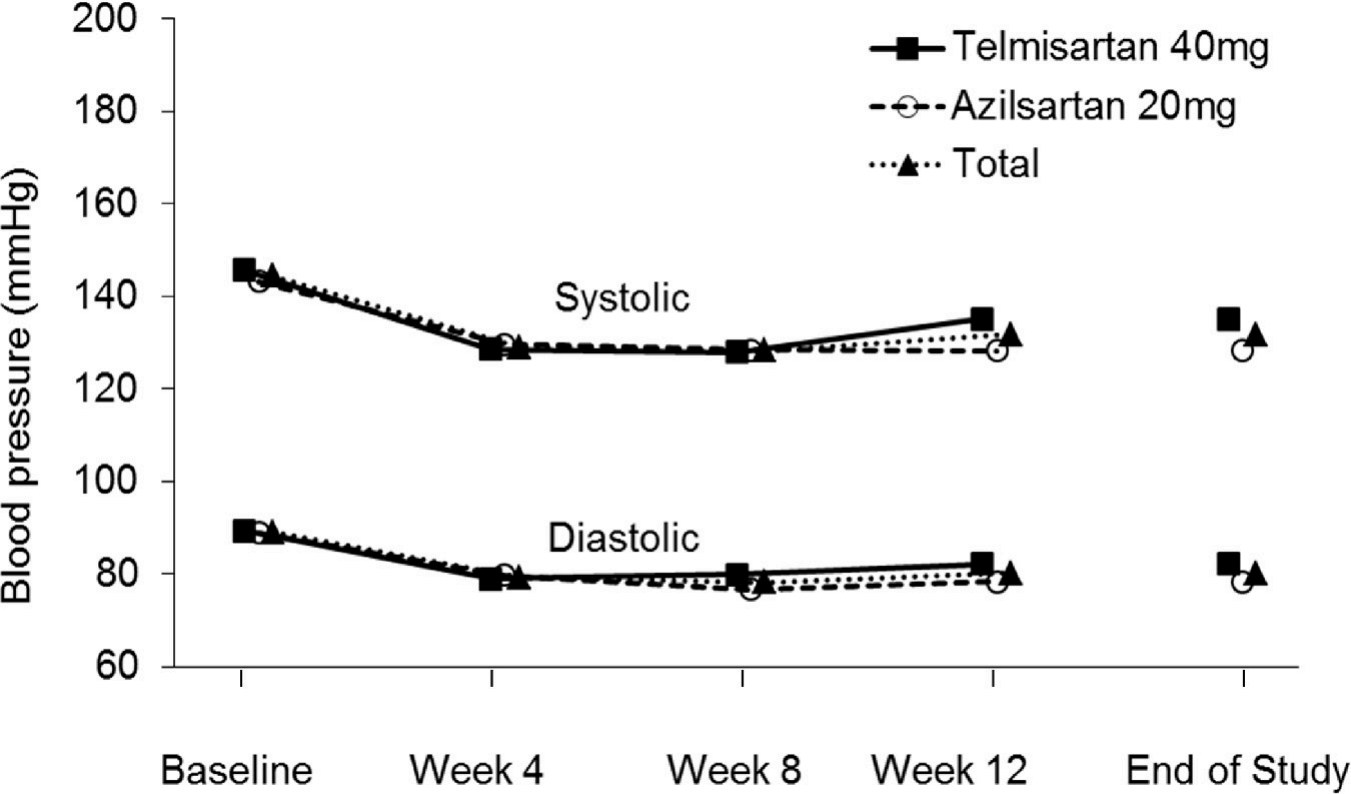
Mean clinic blood pressure values during the treatment period. Sitting systolic and diastolic blood pressure levels were measured at the clinic before initiating dosing at scheduled clinic visits in weeks 4, 8, and 12. Blood pressure levels at the end of the study are derived from values calculated from blood pressure levels at the last dose in all patients receiving at least one dose of the study drugs.

The clinic systolic and diastolic blood pressure values in the azilsartan and telmisartan groups decreased at comparative levels by week 4 and then remained stable at similar low levels until the end of the treatment (Fig 2).

The changes from the baseline values in blood pressure at the end of the treatment period are summarized in Table 5. The mean clinic systolic blood pressure changes from the baseline values at the end of the treatment period were −15.0 mmHg in the azilsartan group and −10.6 mmHg in the telmisartan group. The mean clinic diastolic blood pressure changes were −9.8 mmHg in the azilsartan group and −7.2 mmHg in the telmisartan group. The percentages of patients with normal clinic blood pressure levels at the end of treatment (systolic blood pressure <130 mmHg and diastolic blood pressure <80 mmHg) were 31.3% (5/16) in the azilsartan group and 18.8% (3/16) in the telmisartan group (S2 Table).

**Table 5:** Changes from the baseline values in blood pressure at the end of the treatment period (FAS).

Variable	Azilsartan 20 mg	Telmisartan 40 mg	Total
Clinic SBP (mmHg)	−15.0 [−23.6, −6.4], n = 16	−10.6 [−16.4, −4.7], n = 16	−12.8 [−17.7, −7.8], n = 32
Clinic DBP (mmHg)	−9.8 [−14.6, −5.0], n = 16	−7.2 [−10.2, −4.1], n = 16	−8.5 [−11.2, −5.8], n = 32
Morning SBP (home) (mmHg)	−6.4 [−14.7, 2.0], n = 13	−4.4 [−10.2, 1.4], n = 14	−5.3 [−10.0, -0.65], n = 27
Morning DBP (home) (mmHg)	−4.0 [−8.7, 0.7], n = 13	−3.7 [−7.6, 0.3], n = 14	−3.8 [−6.6, -1.0], n = 27
Before bedtime SBP (home) (mmHg)	−13.2 [−20.9, −5.5], n = 12	−9.9 [−16.6, −3.2], n = 14	−11.4 [−16.2, −6.7], n = 26
Before bedtime DBP (home) (mmHg)	−8.1 [−12.8, −3.3], n = 12	−5.5 [−9.6, −1.3], n = 14	−6.7 [−9.6, −3.7], n = 26

FAS, full analysis set; SBP, systolic blood pressure; DBP, diastolic blood pressure. Mean [95% confidence interval; lower, upper]

We observed no apparent changes from the baseline values in home systolic and diastolic blood pressure in terms of day-by-day and circadian blood pressure variability at the end of treatment in either group (S1–S8 Figs).

### Safety results

The incidences of treatment-emergent AEs (TEAEs) during the treatment period were 35.3% (6/17) in the azilsartan group and 50.0% (8/16) in the telmisartan group as summarized in Table 6. All TEAEs were mild to moderate in severity. We encountered one case of TEAE reported as a mild decrease in blood pressure in the azilsartan group, and this subject discontinued treatment at an early stage.

**Table 6:** Treatment-emergent adverse events during the treatment period (SAS).

System organ class/preferred term^a^	Azilsartan 20 mg		Telmisartan 40 mg	
	(n = 17)		(n = 16)	
	TEAEs	Drug-related TEAEs	TEAE	Drug-relatedTEAEs
Patients with any TEAEs	6 (35.3)		8 (50.0)	
Infections and infestations	2 (11.8)	0 (0)	4 (25.0)	0 (0)
Nasopharyngitis	2 (11.8)	0 (0)	2 (12.5)	0 (0)
Bronchitis	0 (0)	0 (0)	1 (6.3)	0 (0)
Influenza	0 (0)	0 (0)	1 (6.3)	0 (0)
Musculoskeletal and connective tissue disorders	2 (11.8)	0 (0)	1 (6.3)	0 (0)
Back pain	1 (5.9)	0 (0)	1 (6.3)	0 (0)
Spinal osteoarthritis	1 (5.9)	0 (0)	0 (0)	0 (0)
Eye disorders	1 (5.9)	0 (0)	0 (0)	0 (0)
Diabetic retinopathy	1 (5.9)	0 (0)	0 (0)	0 (0)
Gastrointestinal disorders	0 (0)	0 (0)	1 (6.3)	0 (0)
Abdominal pain lower	0 (0)	0 (0)	1 (6.3)	0 (0)
General disorders and administration site conditions	0 (0)	0 (0)	1 (6.3)	0 (0)
Chest discomfort	0 (0)	0 (0)	1 (6.3)	0 (0)
Investigations	1 (5.9)	1 (5.9)	0 (0)	0 (0)
Blood pressure decreased	1 (5.9)	1 (5.9)	0 (0)	0 (0)
Metabolism and nutrition disorders	0 (0)	0 (0)	1 (6.3)	0 (0)
Diabetes mellitus	0 (0)	0 (0)	1 (6.3)	0 (0)
Reproductive system and breast disorders	0 (0)	0 (0)	1 (6.3)	0 (0)
Vaginal hemorrhage	0 (0)	0 (0)	1 (6.3)	0 (0)
Skin and subcutaneous tissue disorders	0 (0)	0 (0)	1 (6.3)	0 (0)
Cutaneous amyloidosis	0 (0)	0 (0)	1 (6.3)	0 (0)

SAS, safety analysis set; TEAEs, Treatment-emergent adverse events; ^a^MedDRA Version 19.0. Values represent the number (%) of patients.

## Discussion

It is important to strictly control blood pressure levels to prevent microvascular and macrovascular diseases in patients with hypertension coexisting with type 2 diabetes mellitus [1]. Various management guidelines recommend ARBs and ACE inhibitors as the first-line antihypertensive treatments for such patients [1, 17, 18] based on the beneficial reduction in the frequency of cardiovascular events and mortality [19] attributed to the insulin resistance-improving effects of ARBs and ACEs.

Here, we compared the effects of azilsartan and telmisartan on the insulin resistance index (HOMA-R) and other diabetes-related variables in patients with grade I or II essential hypertension complicated with type 2 diabetes mellitus. The mean (95% CI) changes from the baseline values in HOMA-R at the end of the treatment period were 0.22 (−1.09–1.52) in the azilsartan group and −0.23 (−0.72–0.27) in the telmisartan group, thus indicating that azilsartan and telmisartan did not clinically remarkably improve insulin resistance, although telmisartan showed a trend of slightly decreasing insulin sensitivity. The mean difference in the changes from the baseline values in HOMA-R between the azilsartan and telmisartan groups was 0.44 (−0.89–1.78). The stratifying factors of the baseline HOMA-R values (<2.5 vs. ≥2.5) and the concurrent use of biguanides (yes vs. no) did not impact the changes in HOMA-R in either group. Moreover, we found no clinically remarkable changes from the baseline values concerning other diabetes-related variables, such as fasting blood glucose, fasting insulin, HbA1c (NGSP), HOMA-β, or 1, 5-AG, in either group.

Despite the results from meta-analysis of randomized studies demonstrating significant improvements in insulin resistance with telmisartan compared with active controls in patients with hypertension [10], the effects of ARBs on insulin resistance are not always reproducible. Studies investigating telmisartan in patients with hypertension and type 2 diabetes mellitus have failed to show beneficial effects on diabetes-related indices, including insulin resistance [20, 21]. Azilsartan improves glucose intolerance and insulin sensitivity in animal models [14, 15]. These findings indicate that the beneficial effect of ARBs on insulin resistance in patients with hypertension and type 2 diabetes mellitus is likely affected by unknown factors. Therefore, although we failed to obtain data indicating the clinical insulin resistance-improving effects of azilsartan and telmisartan, it is considered that the variety of the disease status, rather than the potential activity of ARBs, would affect the effect of ARBs on such patients in the clinical settings. In our study, we selected strict eligibility criteria to ensure a rigorous approach. However, some inclusion and exclusion criteria, such as “inclusion criterion (c): presence of coexisting type 2 diabetes with an HbA1c level of <8.4% and a change in HbA1c of ≤0.3%” and “exclusion criterion (f): fasting blood glucose ≥180 mg/dL and HOMA-R ≤1.6 at the start of the treatment period,” might have been too strict, leading to a smaller sample size in this study than we had hoped to recruit. The small sample size in this exploratory trial may also have led to false negative results. To explore the clinical effects of azilsartan and telmisartan on insulin resistance, we consider that a larger sample size, including patients with disease of various degrees of severity may be necessary. Such a sample will probably be feasible by easing the inclusion and exclusion criteria of patients with hypertension and coexisting type 2 diabetes, increasing the number of study centers and including patients with a high body mass index (BMI).

Both azilsartan and telmisartan resulted in reductions in the clinic diastolic and systolic blood pressure levels, and some patients achieved normal blood pressure levels at the end of the treatment period. Azilsartan (20 mg/day) appeared as effective as telmisartan (40 mg/day) in controlling blood pressure in this patient population. Although the reductions in clinic systolic and diastolic pressure levels in the azilsartan group were numerically higher than those in the telmisartan group, our findings cannot be considered conclusive because the direct comparison of the antihypertensive efficacy of the two treatments was not a pre-specified objective of the study.

Azilsartan and telmisartan both demonstrated favorable safety and tolerability profiles in our patient population, and we did not identify any new safety signals.

The main limitations of this study were the open-label design, small number of enrolled patients, and relatively short treatment period. Double-blind studies utilizing larger patient populations and longer treatment periods are required to conclusively determine the effects of azilsartan and telmisartan on insulin resistance and glycemic control in patients with hypertension and type 2 diabetes mellitus.

In conclusion, we did not observe any clinically remarkable effects of 12-week treatment with azilsartan and telmisartan on the variables of insulin resistance and the disease state of diabetes mellitus, including HOMA-R, fasting blood glucose, fasting insulin, HbA1c (NGSP), HOMA-β, and 1, 5-AG in hypertensive patients with type 2 diabetes mellitus enrolled in this study.

## Supporting information

S1 Table CONSORT checklist. (DOCX)

S2 Table Number and percentage of subjects with normal values of vital signs parameters by visit. (PDF)

S1 Fig Day-by-day blood pressure variability (SD: Standard Deviation) for average of 1st and 2nd measurement for Morning / Bedtime Home SBP. (PDF)

S2 Fig Day-by-day blood pressure variability (SD: Standard Deviation) for average of 1st and 2nd measurement for Morning / Bedtime Home DBP. (PDF)

S3 Fig Day-by-day blood pressure variability (CV: Coefficient of Variation) for average of 1st and 2nd measurement for Morning / Bedtime Home SBP. (PDF)

S4 Fig Day-by-day blood pressure variability (CV: Coefficient of Variation) for average of 1st and 2nd measurement for Morning / Bedtime Home DBP. (PDF)

S5 Fig Circadian blood pressure variability (SD: Standard Deviation) for average of 1st and 2nd measurement for Morning / Bedtime Home SBP. (PDF)

S6 Fig Circadian blood pressure variability (SD: Standard Deviation) for average of 1st and 2nd measurement for Morning / Bedtime Home DBP. (PDF)

S7 Fig Circadian blood pressure variability (CV: Coefficient of Variation) for average of 1st and 2nd measurement for Morning / Bedtime Home SBP. (PDF)

S8 Fig Circadian blood pressure variability (CV: Coefficient of Variation) for average of 1st and 2nd measurement for Morning / Bedtime Home DBP. (PDF)

S1 Protocol Clinical study protocol (English). (PDF)

S2 Protocol Clinical study protocol (Japanese). (PDF)
