# Supplementary material for: Effects of azilsartan compared with telmisartan on insulin resistance in patients with essential hypertension and type 2 diabetes mellitus: An open-label, randomized clinical trial
Source: PLoS One. 2019 Apr 3;14(4):e0214727. doi: 10.1371/journal.pone.0214727 (PMC6447197; doi:10.1371/journal.pone.0214727)
Supplement: S2 Table — (PDF) [file pone.0214727.s002.pdf]

S2 Table. Number and Percentage of Subjects With Normal Clinic Blood Pressure Levels

Study Visit	Telmisartan 40 mg (N=16)	Azilsartan 20 mg (N=17)
Week 4	7 / 16 (43.8)	6 / 16 (37.5)
Week 8	5 / 16 (31.3)	5 / 16 (31.3)
Week 12	3 / 16 (18.8)	5 / 15 (33.3)
End of Study	3 / 16 (18.8)	5 / 16 (31.3)
