# Supplementary material for: Effects of azilsartan compared with telmisartan on insulin resistance in patients with essential hypertension and type 2 diabetes mellitus: An open-label, randomized clinical trial
Source: PLoS One. 2019 Apr 3;14(4):e0214727. doi: 10.1371/journal.pone.0214727 (PMC6447197; doi:10.1371/journal.pone.0214727)
Supplement: S8 Fig — (PDF) [file pone.0214727.s010.pdf]

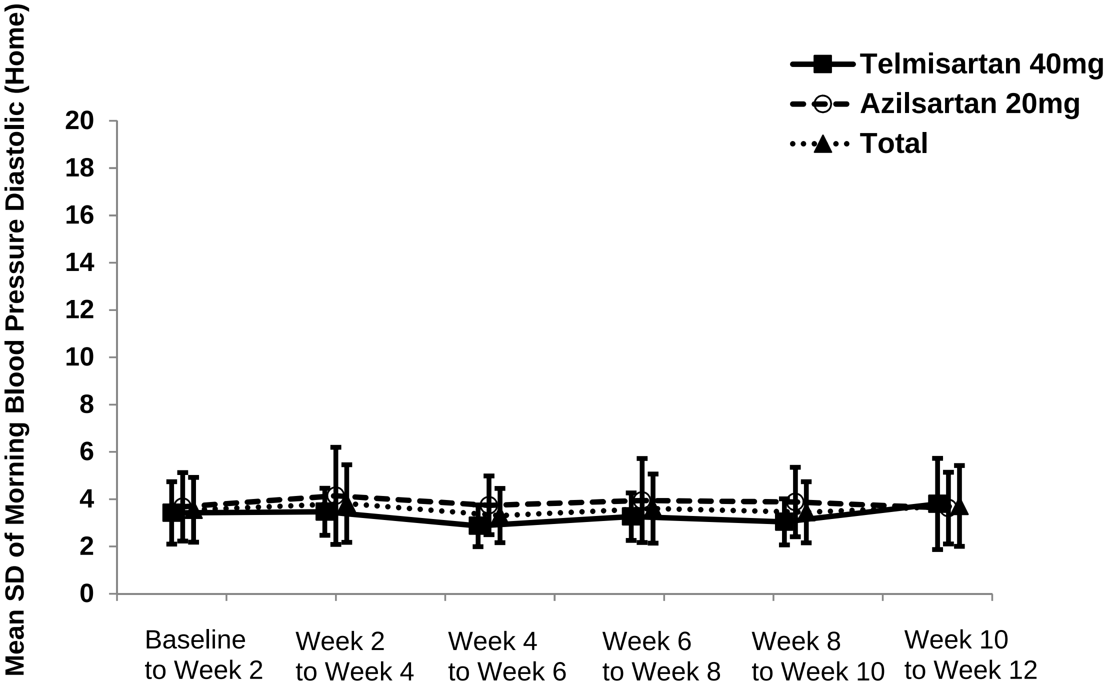

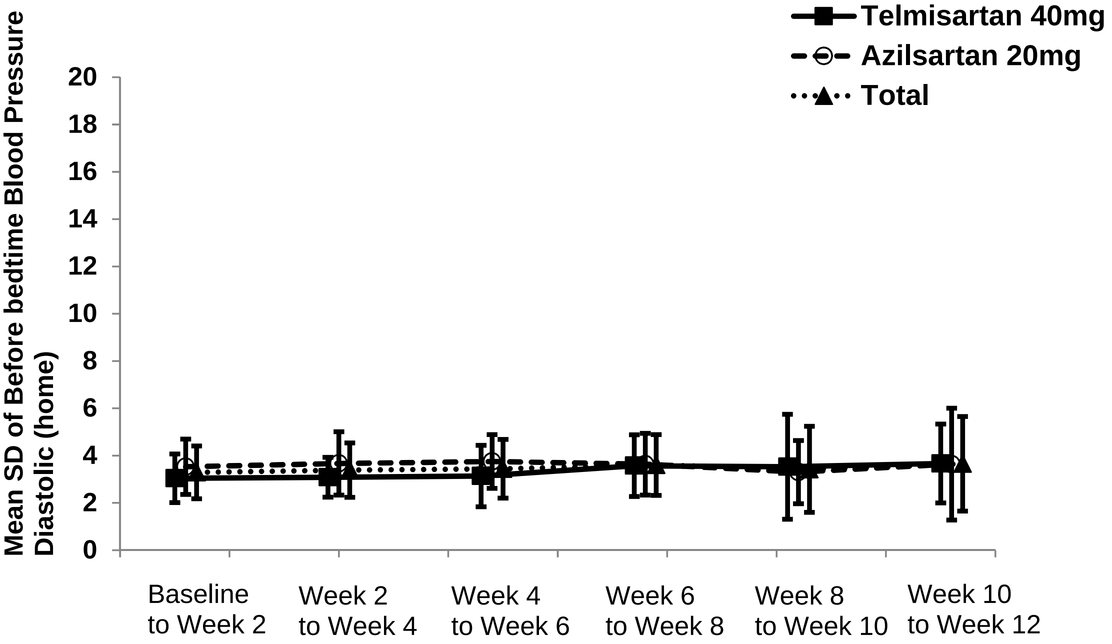

S8 Fig. Circadian blood pressure variability (CV: Coefficient of Variation) for average of 1st and 2nd measurement for Morning / Bedtime Home DBP.

Data are presented as mean ±SD.
