# Supplementary material for: Effects of azilsartan compared with telmisartan on insulin resistance in patients with essential hypertension and type 2 diabetes mellitus: An open-label, randomized clinical trial
Source: PLoS One. 2019 Apr 3;14(4):e0214727. doi: 10.1371/journal.pone.0214727 (PMC6447197; doi:10.1371/journal.pone.0214727)
Supplement: S1 Protocol — (PDF) [file pone.0214727.s011.pdf]

Sponsor-initiated clinical study protocol

A study to explore the effects of Azilsartan compared to Telmisartan on insulin resistance of patients with essential hypertension on type 2 diabetes mellitus by HOMA-R (AT-HOMA)

**Sponsor**	Takeda Pharmaceutical Company Limited
**Clinical study protocol No.**	279/NRP-001
**Version No.**	Second version
**Investigational product**	Azilsartan
**Date of preparation**	October 20, 2015

Amendment history

Date	Version No.	Region
January 31, 2014	Initial version	All investigational sites
October 20, 2015	Second version	All investigational sites

1.0 ADMINISTRATIVE INFORMATION AND STUDY PRINCIPLES

1.1 Contacts and responsibilities for study-related activities

A separate information list will be provided.

1.2 Study principles

This study will be conducted with the highest respect for the individual participants in accordance with the requirements of this clinical study protocol and also in accordance with the following:

- The ethical principles based on the Declaration of Helsinki.
- Ethical Guidelines for Clinical Research (Ministry of Health, Labour and Welfare, revised in 2008).
- All applicable laws and regulations, including, without limitation, data privacy laws and regulations, conflict-of-interest (COI) guidelines and so forth.

TABLE OF CONTENTS

1.0 ADMINISTRATIVE INFORMATION AND STUDY PRINCIPLES 3
1.1 Contacts and responsibilities for study-related activities 3
1.2 Study principles 3
2.0 STUDY SUMMARY 8
3.0 ABBREVIATIONS 15
4.0 INTRODUCTION 16
4.1 Background 16
4.2 Rationale for the proposed study 16
5.0 STUDY OBJECTIVES AND ENDPOINTS 17
5.1 Objectives 17
5.2 Endpoints 17
5.2.1 Primary endpoint 17
5.2.2 Secondary endpoints 17
5.2.3 Other endpoints 17
6.0 STUDY DESIGN 19
6.1 Study design 19
6.2 Justification of study design, dosage, treatment period, and planned number of subjects 20
6.3 Premature termination of the study or investigational sites 21
6.3.1 Criteria for premature termination of the study 21
6.3.2 Criteria for premature termination of investigational sites 21
6.3.3 Procedures for premature termination or Suspension of the Study or investigational sites 21
6.4 Procedures for protocol revision 21
7.0 SELECTION AND DISCONTINUATION OF SUBJECTS 22
7.1 Inclusion criteria 22
7.2 Exclusion criteria 23
7.3 Prohibited concomitant drugs and treatments 25
7.4 Subject management 26
7.5 Criteria for Discontinuation or Withdrawal of a Subject 27

7.6 Procedures for discontinuation of individual subjects 28
8.0 STUDY TREATMENTS 29
8.1 Treatment with investigational product or comparator 29
8.1.1 Dose and regimen 29
8.1.2 Overdose 29
8.2 Treatment with oral hypoglycemic agents 29
8.3 Treatment with drugs other than investigational product/comparator or oral hypoglycemic agents 30
8.4 Creation and retention of randomization code lists 30
8.5 Assignment and dispensing procedures 30
9.0 STUDY PLAN 31
9.1 Study procedures 31
9.1.1 Informed consent 31
9.1.2 Demographic data, medical history, and prior medication 31
9.1.3 Physical examination 32
9.1.4 Weight, height, and BMI 32
9.1.5 Concomitant medications 32
9.1.6 Concurrent diseases 32
9.1.7 Vital signs 33
9.1.8 Home blood pressure 33
9.1.9 Clinical laboratory tests 34
9.1.10 Pregnancy 34
9.1.11 Randomization 34
9.1.12 Documentation of screen failure 34
9.2 Subject treatment compliance 35
9.3 Observation schedule 36
9.3.1 Pre-treatment period 36
9.3.2 Treatment period 36
9.4 Use, retention, and destruction of biological samples 38
10.0 PRETREATMENT EVENTS AND ADVERSE EVENTS 39
10.1 Definitions 39
10.1.1 Pretreatment events 39

10.1.2 Adverse events (AEs) ... 39
10.1.3 Points to consider for PTEs and AEs ... 39
10.1.4 Serious adverse events (SAEs) ... 41
10.1.5 Severity of PTEs or AEs ... 42
10.1.6 Causality of AEs ... 42
10.1.7 Relationship to study procedures ... 43
10.1.8 Date of onset ... 43
10.1.9 Date of resolution ... 43
10.1.10 Actions taken for the investigational product or comparator ... 44
10.1.11 Outcome ... 44
10.2 Procedures ... 45
10.2.1 Collection and reporting of PTEs/AEs ... 45
10.2.2 Collection and reporting of SAEs ... 46
10.2.3 Notification of SAEs to other investigational sites ... 47
10.3 Follow-up of SAEs ... 47
11.0 STUDY-SPECIFIC COMMITTEES ... 48
12.0 DATA MANAGEMENT AND RECORDKEEPING ... 49
12.1 CRFs ... 49
12.2 Record retention ... 50
13.0 STATISTICAL ANALYSIS METHODS ... 51
13.1 Statistical and analytical plans ... 51
13.1.1 Analysis set ... 51
13.1.2 Analysis of demographic and other baseline characteristics ... 51
13.1.3 Efficacy analysis ... 51
13.1.3.3 Other efficacy endpoints ... 52
13.1.3.4 Significance level and confidential coefficient ... 53
13.1.4 Safety analysis ... 53
13.2 Interim analysis and criteria for early termination ... 54
13.3 Determination of sample size ... 54
14.0 QUALITY CONTROL AND QUALITY ASSURANCE ... 55
14.1 Monitoring of investigational sites ... 55
14.2 Deviations from the ethical guidelines for clinical research and protocol ... 55

14.3 Quality assurance audits and regulatory agency inspections 55
15.0 ETHICAL CONDUCT OF THE STUDY 56
15.1 Conflict of interest.. 56
15.2 IEC approval 56
15.3 Information sheet for subjects, informed consent form, and subject authorization.. 56
15.4 Protection of subjects' private/personal information 57
15.5 Benefits and inconveniences to subjects 58
15.5.1 Benefits to subjects.. 58
15.5.2 Inconveniences to subjects 58
15.6 Attribution of study results, access right, and procedure for disclosing procedures 58
15.6.1 Attribution of study results... 58
15.6.2 Right of access to data.. 58
15.6.3 Procedures for disclosing study results 58
15.7 Clinical trial registration and clinical trial results disclosure.......................... 58
15.7.1 Clinical trial registration... 58
15.7.2 Clinical trial results disclosure 59
15.8 Compensation for injury... 59
16.0 REFERENCES.. 60

LIST OF APPENDIX

Appendix A Schedule of Study Procedures... 61
Appendix B Responsibilities of the Investigator... 62
Appendix C Detailed Description of Amendments to Text.. 63

2.0 STUDY SUMMARY

**Sponsor:**<br>Takeda Pharmaceutical Company Limited	**Investigational product:**<br>Azilsartan (trade name: Azilva)
**Title of Study:**<br>A study to explore the effects of Azilsartan compared to Telmisartan on insulin resistance of patients with essential hypertension on type 2 diabetes mellitus by HOMA-R (AT-HOMA)	
**Clinical study protocol No.:**<br>279/NRP-001	
**Study Design:**<br>Multicenter, randomized, open-label, parallel-group exploratory study	
**Objectives:**<br>To explore the effects of azilsartan 20 mg, compared with telmisartan 40 mg, once daily orally for 12 weeks on insulin resistance in patients with essential hypertension complicated by type 2 diabetes mellitus	
**Patient Population:**<br>Patients with essential hypertension complicated by type 2 diabetes mellitus	
**Planned Number of Subjects (as randomized):**<br>Azilsartan group: 20subjects to receive azilsartan 20 mg<br>Telmisartan group: 20 subjects to receive telmisartan 40 mg	**Number of Study Centers:**<br>Approximately 10 sites
**Dosage and Administration:**<br>Subjects will receive azilsartan 20 mg or telmisartan 40 mg once daily in the morning before or after breakfast. However, subjects should come to the sites on scheduled visits without taking their morning dose of antihypertensives and take the dose after completing the specified tests/examinations.	**Route of administration**：<br>Oral
**Run-in Period and Number of Study Visits:**<br>The study design is outlined below.<br>Treatment period: 12 weeks<br>Number of study visits: 4	

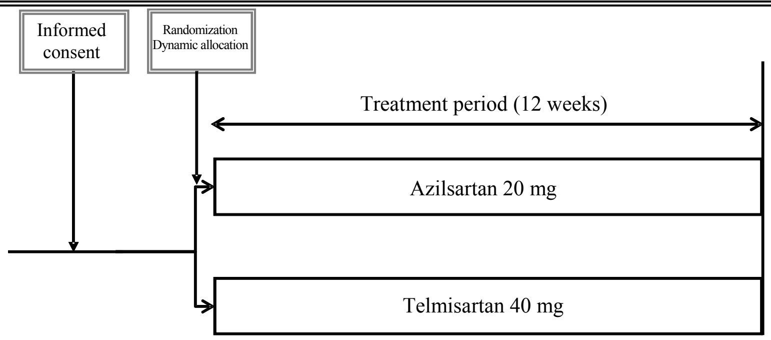

Main Criteria for Inclusion:

1. The subject was given the diagnosis of grade I or II essential hypertension and was judged by the principal investigator or investigator that they can be appropriately treated with azilsartan 20 mg and telmisartan 40 mg.
2. Sitting systolic blood pressure of ≥ 130 mmHg and < 180 mmHg or sitting diastolic blood pressure* of ≥ 80 mmHg and < 110 mmHg at the start of the treatment period (Week 0)

   * Sitting blood pressure will be measured until 2 consecutive stable measurements are obtained (i.e., the difference between 2 measurements: diastolic blood pressure of < 5 mmHg and systolic blood pressure of < 10 mmHg) after resting in a sitting position for at least 5 minutes. The average value of the last 2 measurements will be recorded (the first the decimal place is rounded off).
3. Type 2 diabetes mellitus
4. HbA1c (NGSP value) of < 8.4% during 3 months before informed consent, with a ≤ 0.3% change in HbA1c (peak minus nadir) during 3 months before informed consent
5. No change in diet/exercise therapy during the 3 months before the informed consent in a subject who has been on diet/exercise therapy and instructed to improve life style (e.g., diet and exercise)
6. Age ≥ 20 years at the time of consent
7. Outpatients
8. Capable of providing written consent before participation in this study.

Main Criteria for Exclusion:

1. Grade III essential hypertension (i.e., sitting systolic blood pressure ≥ 180 mmHg or sitting diastolic blood pressure ≥ 110 mmHg), secondary hypertension, or malignant hypertension.
2. Grade II essential hypertension (i.e., sitting systolic blood pressure ≥ 160 mmHg or sitting diastolic blood pressure ≥ 100 mmHg) for which antihypertensive drug(s) are used
3. Use of oral antihypertensive medication within 2 weeks before the start of the treatment period

   Subjects who are on any antihypertensive agent at the time of informed consent can be enrolled in the study only after 2-week washout following informed consent.
4. Use of RAS inhibitors or thiazolidines within 3 months before the start of the treatment period
5. Type 1 diabetes mellitus

6. Fasting blood glucose of < 180 mg/dL and HOMA-R of ≤ 1.6 at the start of the treatment period (Week 0)
7. Receiving or requiring any of the following at the time of informed consent:
    - Insulin, glucagon-like peptide-1 (GLP-1) receptor agonists, or other parenteral hypoglycemic agents
    - Combination therapy with 3 or more oral hypoglycemic agents
8. Change of antidiabetic medication (including dosage change) within 3 months before the start of the treatment period
9. Having diagnosed/treated any of the following cardiovascular diseases within 3 months before the start of the treatment period:
    - Cardiac disease/condition: myocardial infarction, coronary revascularization procedure
    - Cerebrovascular disease: cerebral infarction, cerebral haemorrhage, transient ischaemic attack
    - Advanced hypertensive retinopathy (retinal bleeding or oozing, papilloedema)
10. Having diagnosed/treated any of the following cardiovascular diseases more than 3 months before the start of the treatment period, and is now still in unstable condition.
    - Cardiac disease/condition: myocardial infarction, coronary revascularization procedure
    - Cerebrovascular disease: cerebral infarction, cerebral haemorrhage, transient ischaemic attack
11. Past or current history of any of the following cardiovascular diseases .
    - Cardiac valve stenosis
    - Angina pectoris requiring medication
    - Congestive cardiac failure requiring medication
    - Arrhythmia requiring medication (e.g., paroxysmal atrial fibrillation, severe bradycardia, torsade de pointes, and ventricular fibrillation)
    - Arteriosclerosis obliterans with intermittent claudication or other symptoms
12. Have severe ketosis, diabetic coma or precoma, severe infection, or serious trauma
13. Clinically evident renal disorder (e.g., eGFR <30 mL/min/1.73 m^2)
14. Markedly low bile secretion or severe hepatic disorder
15. History of hypersensitivity or allergy to azilsartan or telmisartan or to both.
16. Presence of hyperkalemia (potassium level ≥ 5.5 mEq/L on laboratory testing)
17. Currently participating in any other clinical study.
18. Pregnant women, women with possible pregnancy, or breast-feeding women.
19. Other patients who are inappropriate for participation in this study in the opinion of the principal investigator or investigator.

Endpoints:

<Primary endpoint>

Change in insulin resistance index (HOMA-R*)

- Change from the start of the treatment period at the end of the treatment period (Week 12)

*：HOMA-R = fasting insulin (μU/mL)×fasting glucose (mg/dL)/405

<Secondary endpoints>

(1) Efficacy:

1) Change in fasting blood glucose from the start of the treatment period at the end of the treatment period (Week 12)

2) Change in fasting insulin

3) Change in HbA1c

4) HOMA-β

HOMA-β=fasting insulin (μU/mL)×360/{fasting glucose (mg/dL) - 63}

5) Change in 1,5-AG

(2) Safety: Adverse events

<Other efficacy endpoints>

1) Change in office blood pressure measured during physical examination

2) Change in early morning blood pressure measured using a home sphygmomanometer

3) Change in blood urea nitrogen level (hereinafter, BUN)

4) Change in serum creatinine level

5) Percentage change in total cholesterol level

6) Percentage change in HDL level

7) Percentage change in LDL level

8) Percentage change in fasting triglyceride level

9) Change in high-molecular-weight adiponectin level

10) Change in plasma aldosterone level

11) Change in plasma renin activity

12) Change in high-sensitive C-reactive protein level (hereinafter, CRP)

13) Change in the urinary albumin/creatinine ratio*

*: Urinary albumin/creatinine ratio (mg/g Cr) = Urinary albumin (mg)/Urinary creatinine (mg/dL)

14) Change in the urinary Na/creatinine ratio*

*: Urinary Na/creatinine ratio (g/day) = Urinary Na (mEq/L)/Urinary creatinine (mg/dL)

Statistical Analysis Methods:

(1) Analysis Set

For data analysis in this study, two different analysis sets, such as Full Analysis Set (FAS) and Safety Analysis Set (SAS) will be used.

The FAS is defined as a group of all enrolled subjects satisfying the following criterion:

- The subject has received either the investigational product or comparator at least once during the study after randomization

The SAS is defined as a group of all enrolled subjects satisfying the following criterion:

- The subject has received either the investigational product or comparator at least once during the study

(2) Efficacy analysis

1. Primary endpoint

[Primary endpoint]

- Insulin resistance index (HOMA-R*)

  Change from the start of the treatment period at the end of the treatment period

  *: HOMA-R = fasting insulin (μU/mL) × fasting glucose (mg/dL)/405

[Statistical analysis methods]

1) Primary analysis

Using the FAS population, descriptive statistics for the primary endpoint will be calculated for each treatment group based on the FAS population. In addition, a two-sided 95% confidence interval for the point estimate and the difference in mean change of insulin resistance index between the two treatment groups (change in the azilsartan group minus change in the telmisartan group) will also be calculated.

2) Secondary analysis

Using the FAS population, descriptive statistics for the primary endpoint will be calculated for each treatment group by setting the baseline HOMA-R values (< 2.5 or ≥ 2.5) and concurrent use of biguanides (Yes or No) as stratified factors. Using the FAS population, descriptive statistics for the primary endpoint will be calculated for each treatment group based on the FAS population. In addition, a two-sided 95% confidence interval for the point estimate and the difference in mean change of insulin resistance index between the two treatment groups (change in the azilsartan group minus change in the telmisartan group) will also be calculated. Descriptive statistics for the primary endpoint will be calculated for each treatment group by setting the baseline HOMA-R values (< 2.5 or ≥ 2.5) and concurrent use of biguanides (Yes or No) as stratified factors.

2. Secondary endpoints

Descriptive statistics for the following endpoints will be calculated for each treatment group based on the FAS population.

- Change in fasting blood glucose

  Change from the start of the treatment period to the end of the treatment period (Week 12)

- Change in fasting insulin

  Change from the start of the treatment period to the end of the treatment period (Week 12)

- Change in HbA1c

  Change from the start of the treatment period to the end of the treatment period (Week 12)

- HOMA-β*

  Change from the start of the treatment period to the end of the treatment period (Week 12)

  *: HOMA-β = fasting insulin (μU/mL)×360/{fasting glucose (mg/dL) - 63}

- Change in 1,5-AG

  Change from the start of the treatment period to the end of the treatment period (Week 12)

3. Other endpoints

- Change in office blood pressure measured during physical examination

  Change from the start of the treatment period at the end of the treatment period

- Change in early morning blood pressure measured using a home sphygmomanometer

  Change from the start of the treatment period at the end of the treatment period

- Change in blood urea nitrogen level (hereinafter, BUN)

  Change from the start of the treatment period at the end of the treatment period

- Change in serum creatinine level

  Change from the start of the treatment period at the end of the treatment period

- Percentage change in total cholesterol level

  Percentage change from the start of the treatment period at the end of the treatment period

- Percentage change in HDL level

  Percentage change from the start of the treatment period at the end of the treatment period

- Percentage change in LDL level

  Percentage change from the start of the treatment period at the end of the treatment period

- Percentage change in fasting triglyceride level

  Percentage change from the start of the treatment period at the end of the treatment period

- Change in high-molecular-weight adiponectin level

  Change from the start of the treatment period at the end of the treatment period

- Change in plasma aldosterone level

  Change from the start of the treatment period at the end of the treatment period

- Change in plasma renin activity

  Change from the start of the treatment period at the end of the treatment period

- Change in high-sensitive C-reactive protein level (hereinafter, CRP)

  Change from the start of the treatment period at the end of the treatment period

- Change in the urinary albumin/creatinine ratio

  Change from the start of the treatment period at the end of the treatment period

- Change in the urinary Na/creatinine ratio

  Change from the start of the treatment period at the end of the treatment period

(3) Safety analysis

- Adverse events

  Adverse events will be translated to their MedDRA terms. The incidences of the following adverse events were tabulated by System Organ Class (SOC) and by Preferred Term (PT) for each treatment group.

- All adverse events
- Adverse events tabulated by severity
- Drug-related adverse events
- Drug-related adverse events tabulated by severity
- Adverse events leading to discontinuation of treatment
- Serious adverse events

Sample Size Justification:

The above sample size has been selected, with consideration to feasibility, as the number of subjects used to explore the effects of azilsartan 20 mg or telmisartan 40 mg on insulin resistance index. This selection was not based on a statistical rationale.

3.0 ABBREVIATIONS

AE	adverse event
ACE	angiotensin converting enzyme
1,5-AG	1,5-anhydroglucitol
ARB	angiotensin II receptor blocker
BMI	body mass index
BUN	Blood urea nitrogen
COI	conflict of interest
CRP	C-reactive protein
EDC	electronic data capture
eGFR	estimate glomerular filtration rate
FDA	food and drug administration
GCP	good clinical practice
GLP-1	Glucagon-like peptide-1
γ-GTP	γ-glutamyl transpeptidase
GOT	glutamic oxaloacetic transaminase
GPT	glutamic pyruvic transaminase
HbA1C	Hemoglobin A1C
HDL	High density lipoprotein
HOMA-R	homeostasis model assessment ratio
HOMA-β	homeostasis model assessment beta cell
LDL	Low density lipoprotein
MedDRA	medical dictionary for regulatory activities
MHRA	medicines and healthcare products regulatory agency
PPARγ	peroxisome proliferator-activated receptor γ
PT	Preferred Term
SAE	serious adverse event
SAP	statistical analysis plan
SOC	System Organ Class
TNF-α	tumor necrosis factor
WHO	world health organization

4.0 INTRODUCTION

4.1 Background

The “Guidelines for the Management of Hypertension 2009” issued by the Japanese Society of Hypertension[1)] position both hypertension and type 2 diabetes mellitus as primary risk factors of major vascular disorders due to atherosclerosis and require strict management of blood pressure and blood glucose level because the onset frequency of cerebrovascular disorder and/or ischemic heart diseases are greatly increased in the concurrent presence of hypertension and type 2 diabetes mellitus.

Hypertension and type 2 diabetes mellitus are major components of metabolic syndrome which have a common underlying factor of insulin resistance. Therefore, in antihypertensive treatment for patients who have both conditions concurrently, due consideration should be paid not only to antihypertensive effects but also to effects on, for example, insulin sensitivity, glucose metabolism, and lipid metabolism. For this reason, in the “Guidelines for the Management of Hypertension 2009 [1)],” angiotensin II receptor blockers (ARBs) and angiotensin converting enzyme (ACE) inhibitors have been recommended as first-line drugs because they improve insulin sensitivity and effectively prevent new onset of diabetes mellitus without effects on lipid metabolism.

On the other hand, adiponectin, a physiologically active substance produced by adipose cells, increases sensitivity to insulin secreted by the pancreas. The production of adiponectin would be promoted through the activation of peroxisome proliferator-activated receptor γ (PPARγ) specifically expressed in fat tissues, which may lead to the improvement in insulin resistance.

Telmisartan is an ARB with excellent antihypertensive effects for which efficacy via PPARγ activation has previously been suggested from nonclinical results[2)]. In clinical settings, telmisartan has been prescribed in expectation of benefits for diabetic patients[3)], including improvement of insulin resistance.

Azilsartan, a new ARB, has a clinically marked hypotensive action compared to other ARBs. In addition, it has been shown in a nonclinical setting that azilsartan increases the expression of PPARγ while decreasing the expression of tumor necrosis factor-α (TNF-α), a cytokine which decreases the sensitivity to insulin[4)]. Therefore, azilsartan is also expected to improve insulin resistance as measured by, for example, homeostasis model assessment ratio (HOMA-R) in clinical settings.[5)]

Nonetheless, the data on the effects of ARBs on insulin resistance as compared to other antihypertensive drugs are limited. Therefore, the present study will explore the effect of azilsartan (vs. telmisartan as a comparator) on diabetes-related indexes including the HOMA-R when administered to essential hypertensive patients complicated with type 2 diabetes mellitus.

4.2 Rationale for the proposed study

This study is intended to contribute to the development of hypertension management strategy through an exploration of the effects of azilsartan or telmisartan on insulin resistance when administered to patients with grade I or II essential hypertension complicated by type 2 diabetes mellitus.

5.0 STUDY OBJECTIVES AND ENDPOINTS

5.1 Objectives

The objective of the present study is to explore the effects of azilsartan 20 mg, compared with telmisartan 40 mg, once daily orally for 12 weeks on insulin resistance in patients with grade I or II essential hypertension complicated by type 2 diabetes mellitus.

5.2 Endpoints

5.2.1 Primary endpoint

Change in insulin resistance index (HOMA-R*)

- Change from the start of the treatment period at the end of the treatment period

*: HOMA-R = fasting insulin (μU/mL)×fasting glucose (mg/dL)/405

5.2.2 Secondary endpoints

(1) Efficacy:

1) Change in fasting blood glucose

2) Change in fasting insulin

3) Change in HbA1c (NGSP value)

4) Change in HOMA-β*

*: HOMA-β = fasting insulin (μU/mL)×360/(fasting glucose [mg/dL] - 63)

5) Change in 1,5-AG

(2) Safety:

Adverse events

5.2.3 Other endpoints

(1) Efficacy:

1) Change in office blood pressure measured during physical examination

2) Change in early morning blood pressure measured using a home sphygmomanometer

3) Change in blood urea nitrogen level (hereinafter, BUN)

4) Change in serum creatinine level

5) Percentage change in total cholesterol level

6) Percentage change in HDL level

7) Percentage change in LDL level

8) Percentage change in fasting triglyceride level

9) Change in high-molecular-weight adiponectin level

10) Change in plasma aldosterone level

11) Change in plasma renin activity

12) Change in high-sensitive C-reactive protein level (hereinafter, CRP)

13) Change in the urinary albumin/creatinine ratio*

*: Urinary albumin/creatinine ratio (mg/g Cr) = Urinary albumin (mg)/Urinary creatinine (mg/dL)

14) Change in the urinary Na/creatinine ratio*

*: Urinary Na/creatinine ratio (g/day) = Urinary Na (mEq/L)/Urinary creatinine (mg/dL)

6.0 STUDY DESIGN

6.1 Study design

(1) Study design

This is a multicenter, randomized, open-label, parallel-group exploratory study.

(2) Subject enrollment

The principal investigator or researchers engaged in the study with the principal investigator (hereinafter, investigators) will conduct tests/examinations in each subject at the start of the treatment period after obtaining informed consent from the subject.

Any antihypertensives used by subjects at the time of informed consent must be discontinued for 2 weeks after informed consent is obtained from them.

(3) Treatment

Subjects who are considered eligible for participation in the study based on the results of eligibility assessment will be randomized to the azilsartan 20 mg or telmisartan 40 mg group at a ratio of 1:1 according to the following stratification factors: HOMA-R at the start of the treatment period (less than 2.5 vs. 2.5 or higher); and the current use of oral hypoglycemic agents (use/non-use of biguanides).

The principal investigator or investigator will prescribe azilsartan 20 mg or telmisartan 40 mg based on the results of treatment group allocation to respective subjects.

Subjects will orally take azilsartan 20 mg or telmisartan 40 mg once daily before or after breakfast in the morning for 12 weeks.

(4) Planned number of subjects (as randomized)

Azilsartan 20 mg group: 20 subjects
Telmisartan 40 mg group: 20 subjects

(5) Number of investigational sites

Approximately 10 sites

(6) Treatment period and number of study visits

A schematic of the study design is included as Figure 6.a.

Treatment period: 12 weeks

Number of study visits: 4

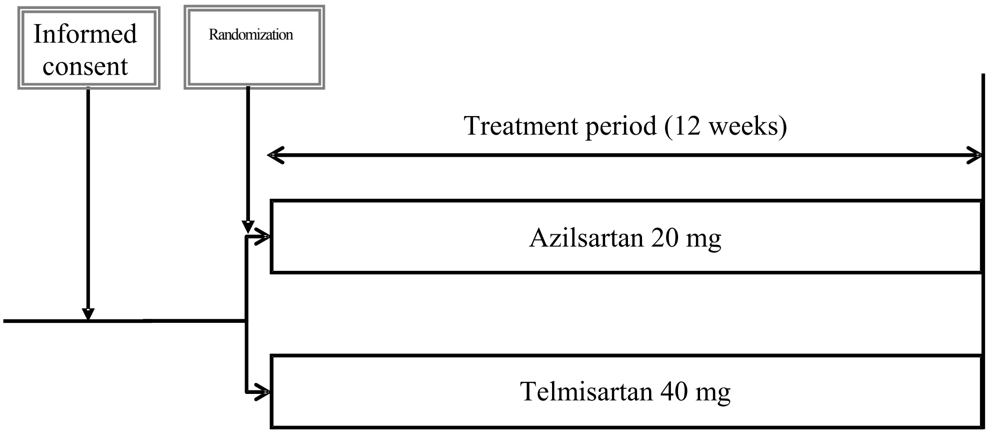

Fig. 6.a Schematic of the study design

6.2 Justification of study design, dosage, treatment period, and planned number of subjects

(1) Justification of study design

A two-parallel-group, random stratified allocation algorithm using the following stratification factors will be used to objectively evaluate the effects of azilsartan 20 mg and telmisartan 40 mg on insulin resistance in patients with grade I or II essential hypertension complicated by type 2 diabetes mellitus: HOMA-R at the start of the treatment period (less than 2.5 vs. 2.5 or higher); and the current use of oral hypoglycemic agents (use/non-use of biguanides).

Among various ARBs, telmisartan, which has been shown to activate PPARγ in non-clinical studies[2)] and has been used in clinical practices in expectation of benefits for diabetic patients[3)], was chosen as a comparator for azilsartan, in order to explore the effects of azilsartan on diabetes-related indexes including the HOMA-R.

Subjects who are on any antihypertensive at the time of signing the informed consent form will undergo a washout period for 2 weeks prior to study treatment to remove the effects of the prior medication (with antihypertensives).

(2) Justification of dosage

In order to assess the effects at the usual clinical dosages, dose levels of 20 mg for azilsartan and 40 mg for telmisartan were chosen.

(3) Justification of treatment period

In light of the fact that the effects of telmisartan in improving insulin resistance have been assessed under 12-week dosing[2)] as well as the fact that the stabilization of the antihypertensive effects of these agents may take 8-12 weeks based on data from the developmental processes of azilsartan and telmisartan, subjects will receive either antihypertensive for 12 weeks.

(4) Justification of the planned number of subjects

Refer to Section 13.3.

6.3 Premature termination of the study or investigational sites

6.3.1 Criteria for premature termination of the study

The study must be terminated by the sponsor if any significant violation of the ethical guidelines that compromises the ability to achieve the primary study objectives or subject safety occurs.

6.3.2 Criteria for premature termination of investigational sites

An investigational site may be terminated prematurely at the discretion of the sponsor if the site, the principal investigator or investigators is found in significant violation of the ethical guidelines, protocol, or contractual agreement, or is unable to ensure adequate conduct of the study.

6.3.3 Procedures for premature termination or Suspension of the Study or investigational sites

In the event that the sponsor or the committee at each investigational site (such as the independent ethics committee [IEC]) decides to terminate or suspend the study or the participation of an investigational site, study-specific procedure for early termination or suspension will be provided by the sponsor. The procedure must be followed by applicable investigational sites during the course of termination or study suspension.

6.4 Procedures for protocol revision

If the protocol needs to be revised, the sponsor will discuss and decide whether to revise the protocol.

The details of each protocol revision must be informed to the principal investigator of each investigational site.

Upon notification, the principal investigator at each investigational site must submit the revised contents to the IEC as necessary according to institutional regulations for review and obtain approval from the director of the site.

7.0 SELECTION AND DISCONTINUATION OF SUBJECTS

7.1 Inclusion criteria

Subjects who meet all of the following criteria will be enrolled in the study:

1. The subject was given the diagnosis of grade I or II essential hypertension and was judged by the principal investigator or investigator that they can be appropriately treated with azilsartan 20 mg and telmisartan 40 mg.
2. Sitting systolic blood pressure of ≥ 130 mmHg and < 180 mmHg or sitting diastolic blood pressure* of ≥ 80 mmHg and < 110 mmHg at the start of the treatment period (Week 0)

   * Sitting blood pressure will be measured until 2 consecutive stable measurements are obtained (i.e., the difference between 2 measurements: diastolic blood pressure of < 5 mmHg and systolic blood pressure of < 10 mmHg) after resting in a sitting position for at least 5 minutes. The average value of the last 2 measurements will be recorded (the first the decimal place is rounded off).

3. Type 2 diabetes mellitus
4. HbA1c (NGSP value) of < 8.4% during 3 months before informed consent, with a ≤ 0.3% change in HbA1c (peak minus nadir) during 3 months before informed consent
5. No change in diet/exercise therapy during the 3 months before the informed consent in a subject who has been on diet/exercise therapy and instructed to improve life style (e.g., diet and exercise)
6. Age ≥ 20 years at the time of consent
7. Outpatient
8. Capable of providing written consent before participation in this study

[Rationale for the inclusion criteria]

1.-3. The target disease of this study is grade I or II essential hypertension.

4. In consideration of the safety of subjects, the upper limit was set at 8.4% (NGSP value) in order to exclude patients with poor glycemic control. Moreover, in consideration of the safety of subjects, a criterion for the change in HbA1c was also adopted in order to exclude patients with unstable glycemic control.
5. A criterion for diet/exercise therapy was employed in consideration of the possible effects of such therapy on the results of assessment.
6. The lower limit of age is 20 years to allow subjects to make a voluntary decision about their participation in the study. No upper age limit is defined for this study in order to accumulate data from various populations as much as practical.
7. Blood pressure during hospitalization is very likely to differ from that during everyday life. To ensure the reliability of evaluation, only outpatients should be included in the study.

8. This is an essential condition for a clinical research.

7.2 Exclusion criteria

Any subject who meets any of the following criteria will not qualify for participation in the study

1. Grade III essential hypertension (i.e., sitting systolic blood pressure ≥ 180 mmHg or sitting diastolic blood pressure ≥ 110 mmHg), secondary hypertension, or malignant hypertension.
2. Grade II essential hypertension (i.e., sitting systolic blood pressure ≥ 160 mmHg or sitting diastolic blood pressure ≥ 100 mmHg) for which antihypertensive drug(s) are used
3. Use of oral antihypertensive medication within 2 weeks before the start of the treatment period

   Subjects who are on any antihypertensive agent at the time of informed consent can be enrolled in the study only after 2-week washout following informed consent.
4. Use of RAS inhibitors or thiazolidines within 3 months before the start of the treatment period
5. Type 1 diabetes mellitus
6. Fasting blood glucose of < 180 mg/dL and HOMA-R of ≤ 1.6 at the start of the treatment period (Week 0)
7. Receiving or requiring treatment with any of the following at the time of informed consent:
   - Insulin, glucagon-like peptide-1 (GLP-1) receptor agonists, or other parenteral hypoglycemic agents
   - Combination therapy with 3 or more oral hypoglycemic agents
8. Change in antidiabetic medication (including change in dosage and administration) within 3 months before the start of the treatment period
9. Having diagnosed/treated any of the following cardiovascular diseases within 3 months before the start of the treatment period:
   - Cardiac disease/condition: myocardial infarction, coronary revascularization procedure
   - Cerebrovascular disease: cerebral infarction, cerebral haemorrhage, transient ischaemic attack
   - Advanced hypertensive retinopathy (retinal bleeding or oozing, papilloedema)
10. Having diagnosed/treated any of the following cardiovascular diseases more than 3 months before the start of the treatment period, and is now still in unstable condition:

- Cardiac disease/condition: myocardial infarction, coronary revascularization procedure
- Cerebrovascular disease: cerebral infarction, cerebral haemorrhage, transient ischaemic attack

11. Past or current history of any of the following cardiovascular diseases
    - Cardiac valve stenosis
    - Angina pectoris requiring medication
    - Congestive cardiac failure requiring medication
    - Arrhythmia requiring medication (e.g., paroxysmal atrial fibrillation, severe bradycardia, torsade de pointes, and ventricular fibrillation)
    - Arteriosclerosis obliterans with intermittent claudication or other symptoms
12. Have severe ketosis, diabetic coma or precoma, severe infection, or serious trauma.
13. Clinically evident renal disorder (e.g., eGFR < 30 mL/min/1.73 m^2)
14. Markedly low bile secretion or severe hepatic disorder
15. History of hypersensitivity or allergy to azilsartan or telmisartan or to both
16. Presence of hyperkalemia (potassium level ≥ 5.5 mEq/L on laboratory testing)
17. Currently participating in any other clinical study
18. Pregnant women, women with possible pregnancy, or breast-feeding women.
19. Other patients who are inappropriate for participation in this study in the opinion of the principal investigator or investigator.

[Rationale for the Exclusion Criteria]

1-6. These diseases are not suitable for the objectives of the study.

7-8. The criterion was adopted in order to accurately assess the efficacy of antihypertensives allocated to subjected (azilsartan or telmisartan).

9-11. To exclude high-risk patients for hypertensive complications, “Associated clinical conditions” under “Factors influencing prognosis” in WHO/ISH 2003 statement on management of hypertension were referenced.

12-13. These criteria were employed to ensure subjects’ safety.

14. The criterion was adopted because these conditions are contraindications of telmisartan.

15. The criterion was employed because both azilsartan and telmisartan are contraindicated in patients with history of hypersensitivity or allergy to ingredients of azilsartan or telmisartan.

16. Since hyperkalemia has been reported as a serious side effect of both azilsartan and telmisartan, the criterion was adopted to ensure subjects' safety.
17. To assure the validity of efficacy evaluation in this study.
18. Azilsartan and telmisartan are contraindicated for use in pregnant or possibly pregnant women. Nonclinical studies of azilsartan and telmisartan have demonstrated the transfer of these drugs into breast milk.
19. This is an essential condition for clinical research.

7.3 Prohibited concomitant drugs and treatments

Concomitant use of the following drugs affecting the HOMA-R or blood pressure is prohibited throughout the duration of the study, from the start of the treatment period until the end of the treatment period. Continuous use of drugs, other than these prohibited concomitant medications, to treat concurrent diseases is permitted on condition that the dosage and administration of oral hypoglycemic agents which have already been used at the time of informed consent should not be changed.

(1) All antihypertensives (including calcium antagonists, diuretics) other than the allocated investigational product

(2) Any other oral hypoglycemic agents than those which have been taken at the time of informed consent

(3) Glucocorticosteroid products (excluding locally-acting drugs such as topical preparations)

(4) Fibrates

(5) Estrogen preparations (including oral contraceptives)

(6) Antianginals (excluding antiplatelet agents such as aspirin)

(7) Anti-arrhythmics (including beta blockers)

(8) Digitalis preparations

(9) Potassium supplements

(10) Thiazolidines

(11) Insulin

(12) GLP-1 receptor agonists

[Rationale for the prohibited concomitant medications and treatments]

(1)-(5) To evaluate appropriately the effects of allocated antihypertensive drugs for the purpose of the study.

(6), (7), (9)-(12) To ensure consistency with the exclusion criteria.

(8) Combination use of azilsartan and these drugs/supplements may cause hyperkalemia.

7.4 Subject management

The principal investigator and investigators should pay attention to the following or instruct subjects on the following.

(1) Subjects should be instructed to take allocated antihypertensive agents as instructed. In cases where any evidence of non-compliance of a subject has been found such as the intake of less than 50% of specified dose since the last visit and no improvement in compliance has been observed, participation of the relevant subject should be terminated according to the situation.

(2) Subjects receiving concomitant oral hypoglycemic agents should be instructed to take the allocated antihyperglycemic agents as instructed, without any change in dosage and administration. In cases where any evidence of non-compliance (such as the intake of less than 50% of specified dose since the last visit) has been found and no improvement in compliance has been observed in a subject, the subject should be excluded from the study depending on the situation.

(3) Full explanation should be given to subjects about the possibility of hypotension due to treatment with antihypertensive agents. Subjects should be instructed to immediately take and keep a lying position at rest in cases where they develop symptoms associated with hypotension (such as dizziness and lightheadedness) at home or elsewhere on days other than scheduled visit days and then, if such symptoms would not be improved, report to the principal investigator or investigator or return to the site without delay to seek further instructions.

(4) When a subject experiences any symptom accompanying increased blood pressure (e.g., headache, palpitations, flash, or perspiration) between scheduled visits, the subject should notify the principal investigator or investigator of the symptoms by telephone, etc. in a timely manner to seek directions. The principal investigator or investigator will instruct the subject to return to the site as necessary.

(5) Subjects will measure home blood pressure at least once each in the morning and evening during the period after signing of informed consent when no antihypertensive is administered. If there is a significant increase in blood pressure, i.e., sitting systolic blood pressure ≥180 mmHg or sitting diastolic blood pressure ≥110 mmHg, the subject should notify the principal investigator or investigator of the increase by telephone, etc. in a timely manner to seek directions. The principal investigator or investigator will instruct the subject to return to the site as necessary.

(6) Concerning blood pressure measurement at home using a home manometer, subjects should be instructed to take and keep a sitting position at rest for 2 minutes or more before reading the monometer as well as to repeat measurement twice at the interval of 30 seconds or more. During the present study, collection of data on blood pressure measurements using a home manometer will be conducted at 4 time points. Subjects should be instructed to keep the same conditions as much as possible at these respective time points.

(7) The principal investigator or investigator should ensure that, in subjects on any diet/exercise therapy, instructions about such therapy (such as specified amount of caloric intake and salt intake) remain constant throughout the study. In addition,

subjects should be instructed to observe the prescribed methods of diet/exercise therapy.

(8) Subjects should be instructed to take glucose or sucrose (sugar) when they develop symptoms of hypoglycemia (abnormal hunger, weakness, tremor of fingers, cold sweat, palpitation and so on) and then, if the symptoms would not be improved, to return to the site without delay.

(9) Subjects should be instructed to avoid taking foods/drinks containing caffeine or smoking within 30 minutes before blood pressure measurement.

(10) Subjects will be instructed to visit the investigational site without taking the allocated antihypertensive in the morning of each visit. The principal investigator or investigator will interview subjects about whether study medication was taken on the day before visit and in the morning of visit.

(11) If it has been judged that safety of treatment with azilsartan 20 mg or telmisartan 40 mg should be specifically confirmed because of, for example, the concurrent presence of mild renal/liver disease or marked decrease/increase in blood pressure, the relevant subjects should be carefully followed through the scheduling of visits to the sites at Week 2 of the treatment period.

(12) Subjects with child-bearing potential should be instructed to practice effective means of contraception. Subjects who become pregnant during the study must immediately notify the principal investigator or investigator of their pregnancy and be withdrawn from the study.

(13) The principal investigator or investigator will instruct subjects not to take the defined prohibited concomitant medications. Subjects should report the details of any drugs taken other than those prescribed by the principal investigator or investigator.

(14) The principal investigator or investigator will interview subjects about the occurrence of any sign/symptom at each visit to record its term, date of onset, severity, outcome, and date of outcome assessment as necessary.

(15) Subjects should be instructed to fast for 10 hours or more before coming to the site on the days of visits for which clinical laboratory tests are planned.

7.5 Criteria for Discontinuation or Withdrawal of a Subject

The principal investigator or investigator will discontinue the study participation of subjects who meet any of the following conditions. The principal investigator or investigator will record the primary reason for discontinuation of each subject from the study in the case report form (CRF) using the following categories.

1. **Pretreatment event or adverse event**

   If a subject experiences a pretreatment event or adverse event that requires an early termination of the study because continued participation imposes an unacceptable risk to the subject’s health, or if a subject is unwilling to continue study participation because of the pretreatment event or adverse event

2. **Major protocol deviation**

   Following randomization, it was found that the subject failed to meet protocol-defined entry criteria or did not adhere to protocol requirements, and continued participation poses an unacceptable risk to the subject's health.

3. **Lost to follow-up**

   The subject did not return to the site and attempts to contact the subject were unsuccessful. In this case, the attempts to contact the subject should be recorded in the source documents.

4. **Voluntary withdrawal**

   The subject wishes to withdraw from the study.

5. **Study termination**

   The sponsor, IEC or regulatory authority has decided to terminate the study.

6. **Pregnancy**

   The female subject is found to be pregnant.

7. **Others**

   For any other reason, the principal investigator or investigator decides to terminate the study. The specific reasons should be recorded in the CRF.

7.6 Procedures for discontinuation of individual subjects

The principal investigator or investigator will terminate a subject's study participation when the subject meets the study termination criteria described in Section 7.5. Individual subjects may discontinue their study participation without giving a reason at any time during the study. Should a subject's participation be discontinued, the principal investigator or investigator should record the primary reason for termination in the CRF and make all efforts to perform all procedures scheduled for the Early Termination Visit.

8.0 STUDY TREATMENTS

8.1 Treatment with investigational product or comparator

8.1.1 Dose and regimen

Subjects will orally take azilsartan 20 mg or telmisartan 40 mg once daily before or after breakfast in the morning. At scheduled visits, each subject should return to the investigational site without taking the allocated drug (azilsartan 20 mg or telmisartan 40 mg) and receive the drug after completing the scheduled tests/examinations.

Table 8.a Dose and regimen

Group	Dose	Route of administration	Regimen
Azilsartan	Azilsartan 20mg	Oral	Once daily, before or after breakfast in the morning
Telmisartan	Telmisartan 40 mg	Oral	Once daily, before or after breakfast in the morning

8.1.2 Overdose

Overdose is defined as a dose of the investigational product or comparator exceeding the dose specified in the clinical study protocol, which can occur because of identified aforethought or mistake (by healthcare personnel or by subjects).

To consistently collect important safety information about overdose, the principal investigator or investigator(s) will record all cases of overdose on the page of "Overdose" in the CRF, irrespective of the presence or absence of accompanying adverse event. Accompanying adverse events will be recorded on the page of "Adverse events" in the CRF, in accordance with Section "10.0 PRETREATMENT EVENTS AND ADVERSE EVENTS".

In addition, serious adverse events associated with overdose will be recorded in accordance with the procedures described in Section "10.2.2 Collection and reporting of SAEs".

If the investigational product or comparator is overdosed in a subject, the principal investigator or investigator(s) will treat the subject depending on symptoms.

8.2 Treatment with oral hypoglycemic agents

Treatment with oral hypoglycemic agents which have already been used at the time of informed consent may be continued, but the dosage and administration of such agents should not be changed during the study.

8.3 Treatment with drugs other than investigational product/comparator or oral hypoglycemic agents

Prohibited concomitant drugs (see Section 7.3) cannot be used. Other treatments should be given in routine practice.

8.4 Creation and retention of randomization code lists

The key controller will create randomization code lists and manage randomization information of each subject. All randomization information will be stored in a secured area, accessible only by authorized personnel to guarantee the independence of such information from the study.

8.5 Assignment and dispensing procedures

To ensure that factors that may affect the efficacy evaluation of the antihypertensives are equally allocated to each group, subjects will be randomized using a stratified allocation algorithm. Randomization will be performed by the Registration Center at the start of treatment (Week 0) considering HOMA-R (less than 2.5 vs. 2.5 or more) at the start of the treatment period and treatment with oral hypoglycemic agents (presence/absence of treatment with biguanides). For stratified allocation, the Registration Center will refer to the randomization code list and the latest number of subjects allocated to each group. The randomization code lists and the information regarding the number of randomized subjects will not be disclosed to the sponsor, coordinating investigator, principal investigators, and investigators until the end of the study.

For randomization of subjects, the principal investigator or principal investigator’s designee will provide necessary information, including subject ID number, to the Registration Center. The ID code of the antihypertensive to be administered to each subject will then be provided by the Registration Center. The principal investigator or investigator will dispense either antihypertensive to each subject according to the ID code assigned to the subject and enter information regarding the dispensed antihypertensive onto the CRF of the subject.

[Randomization factors]

The following factors should be taken into consideration for stratified allocation:

1) HOMA-R: less than 2.5 vs. 2.5 or more

2) Use of biguanides: Yes vs. No

9.0 STUDY PLAN

9.1 Study procedures

The principal investigator or investigator will collect data according to the following procedures. In principle, the tests/examinations, observation and assessments of a subject should be performed by the same principal investigator or investigator. The study schedule is provided in Appendix A.

9.1.1 Informed consent

The procedures for obtaining informed consent are described in Section 15.3.

After the conduct of the study is approved at each investigational site, subjects who meet all of the inclusion criteria and none of the exclusion criteria will be provided with an explanation using the informed consent form/information sheet for subjects and registered on the electronic CRF (eCRF) system in order of signing of the informed consent form to start observation. Discontinuation of prior antihypertensives in a subject to be enrolled in the study can be done only after having obtained the informed consent from the subject. Enrollment of new subjects at each site will be stopped when the planned number of subjects for the site is reached. Overall enrollment will be completed when the total number of randomized subjects reaches the planned sample size for the whole study (n = 20 per group, 40 in total).

The number of subjects to whom explanation for informed consent has been given should be recorded. In addition, a subject code list should be created to protect subject's personal information and a unique subject ID number should be assigned to each subject who has given informed consent and be anonymized (de-identified). This subject ID number will be used throughout the study, and will be kept unchanged.

9.1.2 Demographic data, medical history, and prior medication

(1) Demographic data

Demographic information to be obtained will include birth date, sex, smoking and drinking status, as well as the timing of onset (or diagnosis) of hypertension and diabetes mellitus.

(2) Medical history

Subjects will be interviewed about whether they have any of the symptoms or diseases shown in 1) and 2) below that disappeared or resolved prior to signing of informed consent. Ongoing symptoms or diseases observed at signing of informed consent are considered concurrent diseases rather than medical history (see Section 9.1.6).

1) Circulatory diseases listed below:

- Cardiac disease/condition: myocardial infarction, coronary revascularization procedure

- Cerebrovascular disease: cerebral infarction, cerebral haemorrhage, transient ischaemic attack
- Advanced hypertensive retinopathy (retinal bleeding or oozing, papilloedema)

2) Other symptoms or diseases that are clinically significant in the principal investigator's or investigator's opinion

(3) Prior medication

For each antihypertensive or antidiabetic drug which has been discontinued within 3 months prior to the informed consent, its name and date of withdrawal will be investigated.

9.1.3 Physical examination

All subsequent physical examinations after the start of the treatment period should assess for clinically significant changes from the baseline examination.

9.1.4 Weight, height, and BMI

The height and weight of each subject should be measured. The BMI is calculated using the formula provided below.

Metric: BMI = Weight (kg)/[Height (m)]^2

Height will be measured as a whole number in centimeters and weight will be collected in kilograms to 1 decimal place. BMI will be expressed by rounding off to 1 decimal place. Height measured at the start of the treatment period will be used to calculate BMI.

9.1.5 Concomitant medications

Concomitant medication is any drug given between informed consent and the end of the treatment period in addition to the study medication. At each study visit from the time of informed consent until the end of treatment, any medication taken after the last visit, other than the allocated antihypertensive agents, must be recorded with its name and duration of use. At the time of obtaining the informed consent, subjects should be checked for the use of biguanides.

9.1.6 Concurrent diseases

Concurrent diseases are those ongoing symptoms or diseases that are present at the time of informed consent. Clinically significant laboratory, electrocardiogram (ECG), or physical examination abnormalities detected through baseline examination will also be recorded as concurrent diseases based on principal investigator's or investigator's opinion.

As for concurrent diseases, the following symptoms and diseases (Conditions 1 to 3) will be investigated:

1) Hyperlipemia

2) Following cardiovascular diseases:

- Stable cardiac disease (such as myocardial infarction)
- Stable cerebrovascular disorders (such as cerebral infarction and cerebral hemorrhage)

3) Other symptoms/diseases which have been judged by the principal investigator/investigator to be clinically significant

9.1.7 Vital signs

Sitting systolic and diastolic blood pressure and sitting pulse rate will be measured.

After resting in a sitting position for at least 5 minutes, sitting blood pressure will be measured at least twice repeatedly at the interval of 1 to 2 minutes until 2 consecutive stable measurements are obtained (i.e., the difference between 2 measurements: systolic blood pressure of < 10 mmHg and diastolic blood pressure of < 5 mmHg), and the last 2 consecutive measurements will be recorded.

Intake of caffeine-containing foods and drinks and smoking within 30 minutes prior to blood pressure measurement are prohibited. Blood pressure is measured on the right arm (in case of difficulty in measurement on the right arm for a certain reason, the left arm can be used), and this condition is unchanged throughout the study period.

For blood pressure monitoring beyond Week 2 of the treatment period, each subject should take the allocated antihypertensive on the day before measurement and skip the morning dose on the day of measurement, and the measurement should be started in the morning.

9.1.8 Home blood pressure

For home blood pressure, sitting systolic/diastolic pressure and sitting pulse rate should be measured on awakening and before bedtime.

When the content of informed consent is explained to each subject, the subject should be instructed to measure his/her own blood pressure, along with an explanation that the study will require the measurements of home blood pressure.

At the beginning of the study period, a manometer (for measurement of home blood pressure) will be distributed to each subject, along with a full instruction on the manipulation of the manometer. At each time, blood pressure should be measured after resting in a sitting position for at least 2 minutes and measurement should be repeated twice at the interval of at least 30 seconds.

For more detail, see the procedures formulated separately for the measurements of home blood pressure.

9.1.9 Clinical laboratory tests

Clinical laboratory tests will be performed according to the study schedule (Appendix A). Samples for the laboratory tests will be collected under a fasted condition (fasted for at least 10 hours).

Table 9.a Clinical laboratory tests

Serum chemistry		Urinalysis
Blood urea nitrogen (BUN)<br>Serum creatinine<br>Total cholesterol<br>HDL cholesterol<br>LDL cholesterol (calculated value)<br>Fasting triglyceride	HbA1c (NGSP value)<br>1,5-AG<br>Fasting blood glucose<br>Fasting insulin<br>High-molecular-weight adiponectin<br>Plasma aldosterone<br>Plasma renin activity*<br>High-sensitive CRP	Urinary albumin**<br>Urinary creatinine**<br>Urinary sodium (Na)

* Plasma aldosterone, plasma renin activity: Samples for this test should be collected following resting for at least 15 minutes in the sitting position.

** Using a spot urine sample, urinary albumin level was determined and then corrected with the urinary creatinine level determined using the same spot urine sample: Urinary albumin/creatinine ratio (mg/g Cr) = Urinary albumin (mg)/Urinary creatinine (mg/dL)

The tests listed in the table above will be performed at clinical investigational sites. The principal investigator and investigators should assess the reported results of laboratory tests.

9.1.10 Pregnancy

When a female subject is found pregnant during her participation in the study, if the female subject agrees, the principal investigator or investigator(s) will inform the subject’s primary physician (obstetrician and gynecologist) of her having participated in the study as well as the detailed description of the investigational product she has received.

All reported pregnancies will be followed up to final outcome, using the pregnancy form. The outcome, including any premature termination, must be reported to the sponsor. An evaluation after the birth of the child will also be conducted.

9.1.11 Randomization

Only subjects who meet all of the inclusion criteria and none of the exclusion criteria will be randomized according to Section 8.5.

9.1.12 Documentation of screen failure

For all subjects who signed informed consent and failed screening, a CRF should be completed. The CRF should include the following:

- Date of consent
- Age
- Sex
- Eligibility
- Presence/absence of pretreatment event (if any, please specify it)
- Date of termination and the reason for termination

The primary reason for screen failure will be recorded in the CRF using the following categories:

- Pretreatment event
- Inclusion/exclusion criteria violation
- Major protocol deviation
- Lost to follow-up
- Voluntary withdrawal
- Study termination
- Pregnancy
- Others (the reason should be documented)

Subject ID numbers assigned to subjects who have discontinued before randomization should not be reused.

9.2 Subject treatment compliance

The principal investigator or investigator will check whether each subject complies with the allocated antihypertensive or concomitant antidiabetic drug at each visit. In addition, whether the subject took the investigational product or comparator on the day of the visit will also be investigated. At the end of treatment with the investigational product or comparator, subjects will be classified into four groups depending on how strictly the treatment is complied with: "Properly (compliance rate of ≥ 90%)", "Almost (compliance rate of ≥ 70%)", "Half or more (compliance rate of ≥ 50%)", and "Less than half (compliance rate of < 50%)".

All subjects should be instructed about the dosing requirement throughout the study period. In cases where any evidence of non-compliance (such as the compliance rate of < 50% of specified total doses since the last visit) has been found and no improvement in compliance has been observed in a subject, the subject should be excluded from the study depending on the situation.

9.3 Observation schedule

The schedule for the study-related procedures for all evaluations is shown in Appendix A. The principal investigator or investigator should perform evaluations at the designated time points shown below.

9.3.1 Pre-treatment period

Subjects will be screened for enrollment in the study after the informed consent. Subject's eligibility will be determined in accordance with the inclusion and exclusion criteria as described in Sections 7.1 and 7.2, respectively.

Subjects receiving any antihypertensive at signing of informed consent should discontinue the prior medication after signing of informed consent.

9.3.2 Treatment period

Subjects who are found to be eligible for participation in the study based on the results of tests/examinations, observation and assessments during the pre-treatment period will be randomized as described in Section 8.5.

The tests/examinations, observation and assessments to be performed during the treatment period are described below.

9.3.2.1 Start of the treatment period (Week 0)

- Inclusion/exclusion criteria
- Demographic data
- Medical history
- Prior medication (antihypertensive or antidiabetic drug)
- Physical examination
- Height and weight, BMI
- Concurrent diseases
- Concomitant medications
- Vital signs (sitting systolic and diastolic blood pressure and pulse rate)
- Home blood pressure (sitting systolic/diastolic pressure and pulse rate)
- Clinical laboratory tests
- Pretreatment events

9.3.2.2 Treatment period (Week 2)

The following tests/examinations and assessments will be done when the principal investigator and investigator have judged that such tests/examinations and assessments

are necessary (in cases where, for example, the safety of treatment with azilsartan 20 mg or telmisartan 40 mg should be specifically confirmed because of, for example, the concurrent presence of mild renal/liver disease or marked decrease/increase in blood pressure):

- Physical examination
- Concomitant medications
- Vital signs (sitting systolic and diastolic blood pressure and pulse rate)
- Treatment compliance (antihypertensive or hypoglycemic agent)
- Adverse events

Home Blood Pressure

9.3.2.3 Treatment period (Weeks 4 and 8)

- Physical examination
- Concomitant medications
- Vital signs (sitting systolic and diastolic blood pressure and pulse rate)
- Home blood pressure (sitting systolic/diastolic pressure and pulse rate)
- Treatment compliance (antihypertensive or hypoglycemic agent)
- Adverse events

9.3.2.4 End of the treatment period (Week 12)

- Physical examination
- Weight, BMI
- Concomitant medications
- Vital signs (sitting systolic and diastolic blood pressure and pulse rate)
- Home blood pressure (sitting systolic/diastolic pressure and pulse rate)
- Clinical laboratory tests
- Treatment compliance (antihypertensive or hypoglycemic agent)
- Adverse events
- End of study status

9.3.2.5 Early termination of the treatment period

- Physical examination
- Weight, BMI

- Concomitant medications
- Vital signs (sitting systolic and diastolic blood pressure and pulse rate)
- Home blood pressure (sitting systolic/diastolic pressure and pulse rate)
- Clinical laboratory tests
- Treatment compliance (antihypertensive or hypoglycemic agent)
- Adverse events
- Reason for early termination

9.4 Use, retention, and destruction of biological samples

The principal investigator will organize the management system necessary to protect subject's personal information, and collect, store, and destroy specimens according to the institutional rules.

10.0 PRETREATMENT EVENTS AND ADVERSE EVENTS

10.1 Definitions

10.1.1 Pretreatment events

A pretreatment event (PTE) is defined as any untoward medical occurrence prior to administration of the investigational product or comparator in a subject who has signed informed consent to participate in a study; it does not necessarily have to have a causal relationship with study procedures.

10.1.2 Adverse events (AEs)

An AE is defined as any untoward medical occurrence in a subject receiving a drug; it does not necessarily have to have a causal relationship with this treatment.

An AE can therefore be any unfavorable and unintended sign (e.g., a clinically significant laboratory abnormality), symptom, or disease temporally associated with the use of a drug whether or not it is considered related to the drug.

10.1.3 Points to consider for PTEs and AEs

An untoward finding generally may:

- indicate a new diagnosis or unexpected worsening of a pre-existing condition (intermittent events for a pre-existing condition will not be considered as PTEs or AEs):
- necessitate any intervention or treatment:
- require an invasive diagnostic procedure:
- require discontinuation or a change in the dose of the study antihypertensive or a concomitant medication: or
- be considered unfavorable by the principal investigator.

Diagnoses and signs/symptoms:

PTEs or AEs (hereinafter, PTE or AEs) should be recorded to represent a single diagnosis. Accompanying signs (including abnormal laboratory values or ECG findings) or symptoms should not be recorded as PTEs or AEs. If a diagnosis is unknown, sign(s) or symptom(s) should be recorded appropriately as a PTE(s) or AE(s).

Laboratory values and ECG findings:

Changes in laboratory values or ECG parameters are considered to be PTEs or AEs if they are clinically significant in the principal investigator's or investigator's opinion (i.e., if any intervention or treatment is required or if the principal investigator or investigator judges the change to be beyond the range of normal physiologic fluctuation).

A laboratory re-test and/or continued monitoring of an abnormal value are not considered a therapeutic intervention. In addition, repeated or additional noninvasive testing for verification, evaluation, or monitoring of an abnormality is not considered a therapeutic intervention.

If abnormal laboratory values or ECG findings are the result of pathology for which there is an overall diagnosis as a PTE or AE (e.g., increased creatinine in renal failure), the diagnosis should be reported appropriately as a PTE or AE.

Pre-existing conditions (diseases or symptoms that are present at the time of informed consent):

Pre-existing conditions (present at the time of informed consent) are considered concurrent diseases and should not be recorded as PTEs or AEs. In addition, abnormal findings on the screening examination/observation (i.e., the first test after signing the informed consent) for clinical test values, ECG, and X-ray examination will not be regarded as PTEs, except for abnormal findings associated with the study procedures. However, an abnormal finding associated with the procedures for the screening examination/observation (e.g., internal hemorrhage at blood collection, etc.) will be regarded as a PTE and recorded in the CRF. If the subject experiences a worsening of a concurrent disease, the principal investigator or investigator should record a worsening of the condition as a PTE/AE in the CRF (e.g., “worsening of diabetes”).

If a subject has a pre-existing episodic condition (e.g., asthma, epilepsy), each episode should be captured as a PTE or AE if the episodes become more frequent, serious or severe in nature. If a subject has a chronic concurrent condition (e.g., cataracts, rheumatoid arthritis), worsening of the condition should be captured as AE if the degree of the worsening exceeds that which would be expected. The principal investigator or investigator should ensure that the AE term to be recorded represents the change in the condition from baseline (e.g. “worsening of…”).

Worsening of PTEs or AEs:

If the subject experiences a worsening or complication of a PTE after the start of treatment with the investigational product or comparator, the worsening or complication should be recorded appropriately as a new AE. The principal investigator or investigator should ensure that the AE term to be recorded represents the change in the condition (e.g., “worsening of…”).

Changes in the severity of AEs or serious PTEs:

If the subject experiences changes in the severity of an AE or serious PTE, the event will be recorded once with the maximum severity recorded.

Preplanned surgeries or interventions:

Preplanned surgeries or interventions that were scheduled prior to signing of informed consent are not considered PTEs or AEs. However, if a preplanned surgery or intervention is performed as an emergency procedure because of a worsening of the pre-existing condition, the worsening of the condition should be captured appropriately as a PTE or AE. Complications resulting from any planned surgery should be reported as PTEs or AEs.

Elective surgeries or interventions:

Elective surgeries or interventions (e.g., cosmetic surgery) that are unlikely to affect subject's medical condition should not be recorded as PTEs or AEs. Complications resulting from an elective surgery should be reported as PTEs or AEs.

Insufficient clinical response (lack of efficacy):

Insufficient clinical response, efficacy, or pharmacological action should not be recorded as a PTE or AE. The principal investigator or investigator must make the distinction between worsening of a pre-existing condition and lack of therapeutic efficacy.

Overdose:

Overdose of any medication without manifested symptoms will not be considered an AE. However, any manifested symptoms will be recorded as AEs on the AE page of the CRF.

10.1.4 Serious adverse events (SAEs)

An SAE is defined as any untoward medical occurrence that meets any of the following conditions at any dose (if PTEs meet the definition of SAEs, they should be reported according to the same procedures as those for SAEs [see Sections 10.2.2 and 10.2.3]):

1. results in death,
2. is life threatening*,
3. requires inpatient hospitalization or prolongation of existing hospitalization,
4. results in persistent or significant disability/incapacity,
5. leads to a congenital anomaly/birth defect, or
6. any event which is judged to be medically significant in light of the definitions 1-5 above. A significant medical problem by which subjects are at risks and require certain treatment to prevent an occurrence of the event described in the above categories 1-5 is classified into this category, even if it is not immediately life-threatening or the one causing death or hospital admission. In addition, the events listed in Takeda Medically Significant AE List (Table 10.a) are also included in this category.

* The term "life threatening" refers to an event in which the subject was at risk of death at the time of the event; it does not refer to an event that hypothetically might have caused death if it were more severe.

Table 10.a Takeda Medically Significant AE List

Acute respiratory failure/acute respiratory distress syndrome (ARDS)	Hepatic necrosis
Torsade de Pointes/ventricular fibrillation/ventricular tachycardia	Acute hepatic failure
Malignant hypertension	Anaphylactic shock
Convulsive seizure (including spasm and epilepsy)	Acute renal failure
Agranulocytosis	Pulmonary hypertension
Aplastic anemia	Pulmonary fibrosis
Toxic epidermal necrolysis/ (Stevens-Johnson syndrome)	Confirmed or suspected endotoxin shock
	Confirmed or suspected transmission of infectious agent by a medicinal product
	Neuroleptic Malignant syndrome/malignant hyperthermia
	Spontaneous abortion/stillbirth and fetal death

10.1.5 Severity of PTEs or AEs

The severity of PTEs or AEs will be classified and defined as shown below.

Mild	The event is transient and easily tolerated by the subject.
Moderate	The event interrupts the subject's usual activities.
Severe	The event causes considerable interference with the subject's usual activities.

10.1.6 Causality of AEs

The relationship of each AE to the investigational product or comparator will be classified and defined as shown below.

Related	An AE that follows an apparent temporal sequence from administration of a drug (including the course after withdrawal of the drug), or for which possible involvement of the relevant investigational product or comparator can be argued, although factors other than the drug, such as underlying diseases, concurrent diseases, concomitant drugs, and concurrent treatments, may also be responsible.
Not related	An AE that does not follow an apparent temporal sequence from administration of the relevant investigational product or comparator and/or that can reasonably be explained by other factors, such as underlying diseases, concurrent diseases, concomitant drugs, and concurrent treatments.

10.1.7 Relationship to study procedures

The relationship should be recorded as "Yes" if the principal investigator or investigator considers that there is reasonable possibility that a PTE or AE is due to a study procedure. Otherwise, the relationship should be captured as "No."

10.1.8 Date of onset

The date of onset of PTEs or AE will be determined according to the following rules.

PTEs/AEs	Date of onset
Signs, symptoms, diseases (diagnoses)	The date that the first signs/symptoms were noted by the subject and/or the principal investigator or investigator.
Asymptomatic diseases	The date that a diagnosis was confirmed through a test(s).<br>The date that a diagnosis was confirmed even when the test result indicates an old sign(s) of the disease or an approximate time of its onset.
Worsening or complication of PTEs	The date that the first worsening of diseases/symptoms was noted by the subject and/or the principal investigator or investigator.
When normal values at the time of screening (i.e., the first test after signing the informed consent) are changed to abnormal ones at the subsequent test (for PTEs), or changed to abnormal ones at the test performed after the start of administration of the investigational product or comparator (for AEs)	The date that a clinically significant laboratory abnormality was detected.
When normal values at the time of screening (i.e., the first test after signing the informed consent) are changed to abnormal ones at the subsequent test (for PTEs), or changed to abnormal ones at the test performed after the start of administration of the investigational product or comparator (for AEs)	The date that a clear increase/decrease in a laboratory parameter was clinically confirmed based on the time profile of the parameter.

10.1.9 Date of resolution

The date of resolution of a PTE or AE is the date at which the subject recovered, the event resolved with sequelae, or the subject died. The PTE or AE will be recorded as "ongoing" if the subject has not recovered yet by the end of the study.

10.1.10 Actions taken for the investigational product or comparator

Actions taken for the investigational product or comparator will be classified or defined as shown below.

Drug withdrawn	The investigational product or comparator is discontinued because of the concerned AE (including withdrawal by the subject).
Dose not changed	The dose was not changed after the onset of the concerned AE.<br>The investigational product or comparator is discontinued, reduced, or increased because of an AE(s) other than the concerned AE.<br>The investigational product or comparator was discontinued or reduced for a reason other than the concerned AE, e.g., inadvertent behavior by the subject.
Unknown	When it has not been possible to determine what action has been taken because of loss of follow-up.
Not Applicable	The investigational product or comparator was already completed or discontinued before the onset of the concerned AE.
Dose reduced	The dose was reduced because of the concerned AE (including dose reduction by the subject).
Doe increased	The dose was increased because of the concerned AE (including dose reduction by the subject).
Washout	The investigational product or comparator is suspended or temporarily discontinued because of an AE or other reasons (including subject's own convenience) but resumed thereafter.

10.1.11 Outcome

The outcome of a PTE or AE will be determined according to the following criteria:

Category	Criteria
Resolved	● The signs/symptoms have disappeared or resolved.<br>● The laboratory value returned to the normal range or baseline value (for AEs), or the initial value at the first test after signing informed consent (for PTEs).
Resolving	● The intensity is lowered by one or more stages.<br>● The signs/symptoms has almost disappeared.<br>● The abnormal laboratory value improved, but has not returned to the normal range or the baseline value (for AEs) or the initial value at the first test after signing informed consent (for PTEs).<br>● The subject died from a cause other than the concerned PTE/AE while the condition was resolving (recording of the date of death unnecessary).
Not resolved	● There is no change in the signs/symptoms or laboratory values.<br>● The intensity of the signs/symptoms or laboratory values on the last day of the follow-up period has worsened from the initial intensity.<br>● The PTE/AE is an irreversible congenital anomaly.

	● The subject died from another cause before resolution of the concerned PTE/AE (recording of the date of death unnecessary).
Resolved with sequelae	● The PTE/AE resulted in incapacity that interferes with subject's daily activities.
Fatal	● The concerned PTE/AE was directly related to death.<br>A "direct relationship with the PTE/AE" represents that the concerned PTE/AE caused the death or clearly contributed to the death.<br>● The outcome of a PTE/AE that was observed in the same subject but was determined or assumed not to have directly caused the death is not "fatal."<br>● The date of death will be recorded.
Unknown	● The course of the PTE/AE cannot be followed up as defined in the protocol because of hospital change or residence change.

10.2 Procedures

10.2.1 Collection and reporting of PTEs/AEs

10.2.1.1 PTE/AE collection period

PTEs/AEs will be collected during the periods shown below.

- All PTEs: from the time of informed consent until the start of treatment with the investigational product or comparator
- All AEs: from the start of treatment with the investigational product or comparator until the end of treatment (Week 12) or at discontinuation of treatment

10.2.1.2 Reporting of PTEs/AEs

At each study visit, the principal investigator or investigator will interview the subject about whether any AEs have occurred. A neutral question, such as "How have you been feeling since your last visit?" may be asked. Subjects will be interviewed about any AEs occurring at any other time than study visits during the study.

The principal investigator or investigator must follow up all subjects experiencing a serious PTE until the symptoms resolve, or any clinically significant abnormal laboratory values have returned to the screening values or there is a satisfactory explanation for the change (for PTEs that are permanent or irreversible). Non-serious PTEs, related or unrelated to the study procedure, need not to be followed-up for the purposes of the protocol.

The principal investigator or investigator must follow up all subjects experiencing PTEs or AEs, whether considered associated with the use of the investigational product/comparator or not, until the symptoms resolve, or any clinically significant abnormal laboratory values have returned to the values at the start of the investigational product/comparator or, otherwise (in the case of permanent/irreversible PTEs or AEs), until there is a satisfactory explanation for the changes observed.

All PTEs or AEs will be documented in the CRF. The following information will be documented for each PTEs or AEs: event term, date of onset, date of resolution, severity, causal relationship with the study procedures (Yes or No), causal relationship with the investigational product/comparator (Yes or No), action taken for the investigational product/comparator, outcome of event, and seriousness.

AEs or serious PTEs will be followed up until they have resolved or the principal investigator or investigator decides that no further follow-up is necessary.

Upon request from the sponsor, the principal investigator or investigator should check necessary additional information and data and will complete data entry into the eCRF system within the designated period.

10.2.2 Collection and reporting of SAEs

10.2.2.1 SAEs collection period

SAEs will be collected during the periods shown below.

- All SAEs: from the start of treatment with the investigational product or comparator until the end of the treatment period (Week 12 or at discontinuation of treatment)
- All drug-related SAEs: from the end of the treatment period (Week 12 or at discontinuation of treatment)

10.2.2.2 Reporting of SAEs

Any SAE occurring during the AE collection period should be reported according to the following procedure. In addition, serious PTEs defined in Section 10.1.4 will be reported according to the procedure similar to that for SAEs.

The principal investigator or investigators will report any SAE to the director of the investigational site and the sponsor (for the contact information, refer to the attachment) within 24 hours of first onset or notification of the event. In addition, the principal investigator will submit an official report detailing the event within 10 calendar days.

The following information must be included in the report, along with other information as much as possible:

- Title of Study
- Subject ID number
- Name of investigational site
- Principal investigator’s name
- Term and clinical course of the SAE (onset date, reason for seriousness assessment, name of the antihypertensive at use, causality with the antihypertensive, clinical course, and outcome)

- Action taken for the SAE (interruption of new enrollment, revision of the informed consent form/information sheet for subjects, and/or re-consent from other subjects)

10.2.3 Notification of SAEs to other investigational sites

Once any SAE is reported by the principal investigator, the director of each investigational site should consult with the IEC and notify all other investigational sites (via the sponsor) of the following information, along with the original report from the principal investigator. If a PTE meets the definition of serious events in Section 10.1.4, they should be reported according to the procedures for SAEs.

- Date, summary, and results of review by the IEC, necessary actions to be taken, etc.

10.3 Follow-up of SAEs

The principal investigator or investigator should follow up all SAEs until resolution or permanent outcome of the event is determined. If a PTE meets the definition of serious events in Section 10.1.4, they should be followed up according to the procedures for SAEs.

If information regarding a SAE, including its outcome, has been changed, the principal investigator or investigator should submit a report describing the change(s) to the director of the investigational site and the sponsor. Relevant data collected at the investigational site (e.g., ECG charts, laboratory test values, discharge summary, postmortem results) should be sent to the sponsor or IEC upon request.

11.0 STUDY-SPECIFIC COMMITTEES

No special committee will be established for this study.

12.0 DATA MANAGEMENT AND RECORDKEEPING

The full details of procedures for data management will be documented in the Data Management Plan. PTEs, AEs, medical history, and concurrent diseases will be translated using the Medical Dictionary for Regulatory Activities (MedDRA). Drugs will be translated using the WHO Drug Dictionary.

12.1 CRFs

The principal investigator or investigator must complete CRFs for each subject who signs informed consent.

The sponsor or its designee will provide the principal investigator, investigators, and collaborator(s) with access to eCRFs (electronic data capture [EDC] system). Before using the EDC system, the sponsor will provide education to the principal investigator, investigator(s), and collaborator(s). CRFs will be created by directly entering data in the EDC system. eCRFs must be completed in Japanese.

Any change or correction to the CRF will be recorded as an audit trail describing the following information: information before and after the change/correction, the name of person putting the change/correction, the date of putting the change/correction, and the reason for the change/correction.

The principal investigator will check for the accuracy and integrity of the CRF then put his/her digital signature on it. Furthermore, the principal investigator must retain full responsibility for the accuracy and authenticity of all data entered on the eCRFs.

Data to be directly entered in the CRFs in the EDC system are as follows (excluding cases where there are comments in the source materials):

- Comments from the principal investigator or investigator
- Seriousness, severity, causal relationship with the investigational product or comparator, study procedures and outcome

The sponsor or its designee will visit each investigational site as necessary to check for the accuracy and integrity of the CRF prepared at each site. The sponsor or its designee will also access the study-associated subject’s medical record and hospital record as necessary to ensure the accuracy of the CRF at each site.

The CRF thus completed will belong to the sponsor, and the coordinating investigator, principal investigator, and investigators cannot disclose the CRF to others (other than the regulatory authority) without written authorization.

When the principal investigator or investigator(s) needs to change or correct on the CRF after locking of the database, the change/correction should be made using a Data Clarification Form provided by the sponsor. The principal investigator will check the accuracy and integrity of the Data Clarification Form then sign and seal it, along with the date of checking it.

12.2 Record retention

The principal investigator or the director of the investigational site should keep the following documents that include study-specific documents to enable inspections or audits by regulatory authorities, the sponsor or its designees; the subject code lists, subject's medical records, all original signed and dated informed consent forms, and eCRF (copy) including Data Clarification Form (copy) and audit trails. The principal investigator or the director of each investigational site should also retain specified essential documents until the termination of the study or for 5 years after the completion of the study. If the sponsor requires longer retention of these documents, the institution will discuss with the sponsor about the period and condition of retention.

Furthermore, the principal investigator and the director of each investigational site should retain the essential documents until the sponsor notifies the principal investigator and the director of each site of the documents being unnecessary.

13.0 STATISTICAL ANALYSIS METHODS

13.1 Statistical and analytical plans

The important contents of statistical analysis for this study are presented below.

The persons in charge of analyses will prepare and finalize a statistical analysis plan to provide further details regarding the definition of analysis variables and analysis methodology to address all study objectives.

13.1.1 Analysis set

For data analysis in this study, two different analysis sets, such as Full Analysis Set (FAS) and Safety Analysis Set (SAS) will be used.

The FAS is defined as all enrolled subjects who meet the following criterion:

- The subject has received either the investigational product or comparator at least once during the study after randomization

The SAS is defined as all enrolled subjects who meet the following criterion:

- The subject has received either the investigational product or comparator at least once during the study

13.1.1.1 Efficacy data set

The efficacy analysis will be performed using the FAS.

13.1.1.2 Safety data set

The safety will be analyzed using the SAS.

13.1.2 Analysis of demographic and other baseline characteristics

The primary subject’s baseline characteristics data collected from all randomized subjects will be summarized for each treatment group and for all groups combined.

13.1.3 Efficacy analysis

13.1.3.1 Primary endpoint

(1) Primary endpoint

- Insulin resistance index (HOMA-R*)

Change from the start of the treatment period at the end of the treatment period

*: HOMA-R = fasting insulin (μU/mL)×fasting glucose (mg/dL)/405

(2) Analysis of the primary endpoint

Using the FAS population, descriptive statistics for the primary endpoint will be calculated for each treatment group by setting the baseline HOMA-R values (< 2.5 or ≥ 2.5) and concurrent use of biguanides (Yes or No) as stratified factors.

13.1.3.2 Secondary endpoints

Descriptive statistics for the following endpoints will be calculated for each treatment group based on the FAS population:

● Change in fasting blood glucose

Change from the start of the treatment period at the end of the treatment period

● Change in fasting insulin

Change from the start of the treatment period at the end of the treatment period

● Change in HbA1c

Change from the start of the treatment period at the end of the treatment period

● Change in HOMA-β*

Change from the start of the treatment period at the end of the treatment period

*: HOMA-β=fasting insulin (μU/mL)×360/(fasting glucose [mg/dL] - 63)

● Change in 1,5-AG

Change from the start of the treatment period to at the end of the treatment period (Week 12)

13.1.3.3 Other efficacy endpoints

Descriptive statistics for the following endpoint will be calculated for each treatment group based on the FAS population:

● Change in office blood pressure measured during physical examination

Change from the start of the treatment period at the end of the treatment period

● Change in early morning blood pressure measured using a home sphygmomanometer

Change from the start of the treatment period at the end of the treatment period

● Change in BUN level

Change from the start of the treatment period at the end of the treatment period

● Change in serum creatinine level

Change from the start of the treatment period at the end of the treatment period

● Percentage change in total cholesterol level

Percentage change from the start of the treatment period at the end of the treatment period

● Percentage change in HDL level

Percentage change from the start of the treatment period at the end of the treatment period

● Percentage change in LDL level

Percentage change from the start of the treatment period at the end of the treatment period

● Percentage change in fasting triglyceride level

Percentage change from the start of the treatment period at the end of the treatment period

● Change in high-molecular-weight adiponectin

Change from the start of the treatment period at the end of the treatment period

● Change in plasma aldosterone

Change from the start of the treatment period at the end of the treatment period

● Change in plasma renin activity

Change from the start of the treatment period at the end of the treatment period

● Change in high-sensitive C-reactive protein level (hereinafter, CRP)

Change from the start of the treatment period at the end of the treatment period

● Change in the urinary albumin/creatinine ratio*

Change from the start of the treatment period at the end of the treatment period

*: Urinary albumin/creatinine ratio (mg/g Cr) = Urinary albumin (mg)/Urinary creatinine (g)

● Change in the urinary Na/creatinine ratio*

Change from the start of the treatment period at the end of the treatment period

*: Urinary Na/creatinine ratio (g/day) = Urinary Na (mEq/L)/Urinary creatinine (mg/dL)

13.1.3.4 Significance level and confidential coefficient

Confidential coefficient: two-sided 95% confidence interval

13.1.4 Safety analysis

● Adverse events

Adverse events will be translated to their MedDRA terms. The incidences of the following adverse events were tabulated by System Organ Class (SOC) and by Preferred Term (PT) for each treatment group.

● All adverse events

● Adverse events tabulated by severity

- Drug-related adverse events
- Drug-related adverse events tabulated by severity
- Adverse events leading to discontinuation of treatment
- Serious adverse events

13.2 Interim analysis and criteria for early termination

No interim analysis is planned.

13.3 Determination of sample size

Planned number of randomized subjects

Azilsartan 20 mg group: 20 subjects

Telmisartan 40 mg group: 20subjects

[Sample size justification]

The above sample size has been selected, with consideration to feasibility, as the number of subjects used to explore the effects of azilsartan 20 mg or telmisartan 40 mg on insulin resistance. This selection was not based on a statistical rationale.

14.0 QUALITY CONTROL AND QUALITY ASSURANCE

14.1 Monitoring of investigational sites

In order to check whether the study is being conducted in accordance with the all the requirements described in the clinical study protocol, the sponsor or its designee will perform monitoring of each investigational site at regular intervals throughout the study period. In the monitoring, the data recorded on the CRF will be checked by comparing them with those in the source documents. The source documents are referred to as the original documents, original data, and original records. The principal investigator and the director of each investigational site will guarantee that the sponsor or its designee and the IEC are given access to the source documents.

The sponsor or its designee will be given access to the subject code list, medical record, and signed and dated original informed consent form. The sponsor or its designee will check whether the study is being appropriately conducted in accordance with the clinical study protocol. The principal investigator, investigators, and other personnel engaged in the study are required to strive to be cooperative and spend enough time so that the monitoring can be done smoothly when the sponsor or its designee visits the investigational sites.

Detail procedures for monitoring will be described in the Monitoring Plan.

14.2 Deviations from the ethical guidelines for clinical research and protocol

The principal investigator will document any deviations from the Ethical Guidelines for Clinical Research and the study protocol. If any deviation occurs, the principal investigator will notify the director of the investigational site and the sponsor of it in a timely manner. The principal investigator will consult with the coordinating investigator about the necessity of protocol revision if necessary. If the sponsor decides to revise the protocol, the principal investigator will submit a draft revision to the director of the institution for approval of the IEC.

The full details of the rules regarding deviations will be defined in the Deviation Rules.

14.3 Quality assurance audits and regulatory agency inspections

Each study center also may be subject to quality assurance audits by the sponsor or its designees. In this circumstance, the sponsor-designated auditor will contact the site in advance to arrange an auditing visit. The auditor may ask to visit the facilities where laboratory samples are collected and any other facilities used during the study. In addition, this study may be inspected by regulatory agencies, including those of foreign governments (e.g., the Food and Drug Administration [FDA], the United Kingdom Medicines and Healthcare products Regulatory Agency [MHRA]). If the investigational site is contacted for an inspection by a regulatory body, the sponsor should be notified immediately. The principal investigator and the director of the institution should guarantee access for auditors to all original study documents.

15.0 ETHICAL CONDUCT OF THE STUDY

This study will be conducted with the highest respect for the individual subjects according to the protocol, the ethical principles that have their origin in the Declaration of Helsinki, and the Ethical Guidelines for Clinical Research (Ministry of Health, Labour, and Welfare, revised in 2008).

15.1 Conflict of interest

Prior to the conduct of the study, the principal investigator should assure that there will be no conflict of interest (COI) arising from the participation in the study in accordance with the requirements specified at the clinical trial institution[7)-11)].

Each investigational site should comply with all requirements specified by the IEC, including the COI self-statement form, the study protocol, and the information sheet for subjects/informed consent form.

15.2 IEC approval

The principal investigator will provide the director of the institution with the protocol, the information sheet for subjects/informed consent form, and other regulation-specified documents for IEC's review on the conduct or continuation of the study. The director of the site will request the IEC to review the protocol as well as other necessary information for the proper conduct of the study to ensure that the clinical study plan meets the Ethical Guidelines for Clinical Research. The principal investigator will obtain a record, which should be approved by the IEC and permitted by the director of the institution prior to the conduct or continuation of the study.

The investigational sites must comply with all requirements specified by the IEC, including all protocol revisions, revisions of the information sheet for subjects/informed consent form, safety reports according to regulatory requirements, study progress reports made at an IEC-specified frequency, and submission of study completion report, and notifications to the IEC.

15.3 Information sheet for subjects, informed consent form, and subject authorization

Written consent documents will embody the elements of informed consent as described in the Declaration of Helsinki, the Ethical Guidelines for Clinical Research, and all applicable laws and regulations. The informed consent form and information sheet for subjects will describe the planned and permitted uses (in or outside Japan, for third parties) and disclosures of subject's personal and personal health information for purposes of conducting the study. The informed consent form and the information sheet will explain in detail the nature of the study, its objectives, and potential risks and benefits. The informed consent form will detail the requirements of the participant and the fact that he or she is free to withdraw at any time without giving a reason and without any negative effect on the further medical care.

The principal investigator is responsible for the preparation, contents, and IEC approval of the informed consent form and subject information sheet. The informed consent form and information sheet must be approved by the IEC prior to use.

The informed consent form and subject information sheet must be written in a language fully comprehensible to the candidate subject. It is the responsibility of the principal investigator or investigator to fully explain the details of the informed consent form and subject information sheet to the subject. Information should be given in both oral and written form whenever possible and in a manner deemed appropriate by the IEC.

The principal investigator or investigator must provide each candidate subject with ample opportunity and time to: (1) inquire about details of the study and (2) decide whether or not to participate in the study. If the subject determines he or she will participate in the study, then the informed consent form must be signed/sealed and dated by the subject prior to the subject entering into the study. The principal investigator or investigator will instruct the subject to sign using their legal names, not nicknames, using a blue or black ballpoint ink pen. The principal investigator or investigator must also sign/seal and date the informed consent form prior to subject entering into the study.

Once signed/sealed, the original informed consent form will be retained by the principal investigator or investigator. The principal investigator or investigator must document the date the subject signs/seals the informed consent form in the subject's medical record. Copies of the signed/sealed informed consent form should be given to the subject.

All revised informed consent forms/information sheets for subjects must be signed/sealed by relevant subjects in the same manner as the original informed consent, and the original signed forms must be kept. The date the revised consent was obtained should be recorded in the subject's medical record, and the subject should receive a copy of the revised informed consent form.

15.4 Protection of subjects' private/personal information

The sponsor and its designees affirm and uphold the principle of the protection of subjects' private/personal information. Throughout this study, subject's source data will only be linked to the study-specific database or documentation via a unique subject identification number. As permitted by all applicable laws and regulations, limited subject attributes, such as sex and date of birth, may be used to identify the subject and to verify the accuracy of the subject's unique identification number.

To verify that the study is being conducted in compliance with this protocol according to the Ethical Guidelines for Clinical Research, the sponsor requires the principal investigator to permit its designee, representatives from any regulatory authority, the designated auditors, and the IECs to review subject's original medical records (source data or documents), including laboratory test results, admission and discharge summaries during a subject's study participation, and autopsy reports. The principal investigator or investigator must obtain specific authorization of the subject as part of the informed consent process for access to subject's original medical records by the sponsor's designees and representatives from regulatory authorizes (see Section 15.3).

Copies of any subject source documents that are provided to the sponsor, must have certain personally identifiable information removed (i.e., subject's name, address, and other identifier fields not collected on the subject's eCRF).

15.5 Benefits and inconveniences to subjects

15.5.1 Benefits to subjects

Subjects will be able to know, for example, the status of their hypertension and diabetes mellitus as a result of their participation in this study.

15.5.2 Inconveniences to subjects

Subjects need to visit the clinic and undergo tests/examinations more frequently during their participation in the study than usual routine treatment, which may lead to a larger burden on the subjects. In addition, subjects are not allowed to take any antihypertensive drug during a certain period of time before the initiation of treatment and, in the treatment period, some restrictions may be put on treatment for hypertension, diabetes mellitus and concurrent diseases.

15.6 Attribution of study results, access right, and procedure for disclosing procedures

15.6.1 Attribution of study results

All data and information from the study belong to the sponsor.

15.6.2 Right of access to data

The right of access to all data and information from the study will be given to personnel approved by the sponsor.

15.6.3 Procedures for disclosing study results

During the study and after the end of the study, the sponsor or its designee should summarize and disclose the results in a medical journal(s) and/or an academic meeting(s) in a timely manner.

The sponsor may publish any data and information from the study.

To disclose any information collected through this study in academic meetings, etc., the principal investigator must obtain a prior written permission from the sponsor.

15.7 Clinical trial registration and clinical trial results disclosure

15.7.1 Clinical trial registration

In order to ensure that information on clinical trials reaches the public in a timely manner and to comply with applicable law, regulation and guidance, the sponsor or its designee will register this study and the synopsis of the study plan on a publicly accessible website (UMIN-CTR, etc.) before trial initiation.

15.7.2 Clinical trial results disclosure

The sponsor or its designee will post the results of this clinical trial, regardless of outcome, on the above database (UMIN-CTR, etc.).

15.8 Compensation for injury

Each subject in the study must be compensated in accordance with the regulations applicable to the site where the subject is participating. The sponsor will obtain clinical study insurance against the risk of injury to clinical study subjects.

16.0 REFERENCES

1) Guidelines for the Management of Hypertension 2009 (the Japanese Society of Hypertension).

2) Guidelines for the Management of Diabetes Mellitus 2012-2013 (the Japanese Diabetes Society).

3) Benson S. C. et al.: Identification of Telmisartan as a unique Angiotensin II receptor antagonist with selective PPARγ-modulating activity. Hypertension 2004; 43; 993-1002

4) Watanabe M. et al: Effects of Telmisartan on insulin resistance in Japanese type 2 diabetic patients. Inter Med. 2010; 49; 1843-1847

5) Iwai M. et al.: TAK-536, a new AT1 receptor blocker, improves glucose intolerance and adipocyte differentiation. Am J Hypertension 2007; 20; 579-586

6) Kazuhiro Hiramitsu et al.: Usefulness of azilsartan (Azilva tablet) in patients with hypertension complicated by diabetes mellitus. Blood Pressure 2013; 20 (1); 94-99

7) Conflict of interest working group report (Ministry of Education, Culture, Sports, Science and Technology, November 1, 2002).

8) Guidelines for formulation of conflict of interest policy in clinical research (Study Group for Ethics and Conflict of Interest, March 2006).

9) Guidelines for Management of Conflict of Interest (COI) in Health and Labour Sciences Research (March 31, 2008).

10) Guidelines for COI Management in Medical Research (Conflict of Interest Committee, Japanese Association of Medical Sciences Task Force, February 2011).

11) Common Guidelines for Conflict of Interest (COI) in Clinical Research (Japanese Society of Internal Medicine, Japan Society of Hepatology, Japanese Circulation Society, Japan Endocrine Society, Japan Diabetes Society, Japanese Respiratory Society, Japanese Society of Hematology, Japanese Society of Allergology, and Japanese Association for Infectious Diseases (August 2011).

Appendix A Schedule of Study Procedures

				Treatment period				
Time in study	Week	-	0	2**	4	8	12	Withdrawal***
	Day	-	1*	15	29	57	85	-
VISIT Number		-	1	2	3	4	5	-
Informed consent		X(a)						
Inclusion/exclusion criteria		X(a)	X					
Demographic data			X					
Medical history/Prior medication (antihypertensive medication)			X					
Physical examination			X	(X)	X	X	X	X
Height			X					
Body weight, BMI			X				X	X
Concurrent disease			X					
Concomitant medication			← X →					
Vital signs Sitting blood pressure/pulse rate			X	(X)	X	X	X	X
Home blood pressure			← X →					
Laboratory tests			X(b)(d)				X(b)	X(c)
Prescription of antihypertensive medication			X	(X)	X	X		
Treatment compliance				(X)	X	X	X	X
Evaluation for adverse events, etc.			← X →					

X: Implementation of the examination/observation

← X → : Examination/observation implemented throughout the study.

(a) In subjects taking any antihypertensive medication at the time of consent, their antihypertensive medication will be discontinued after the consent.

(b) Fasting values will be measured (after fasting for 10 hours or longer).

(c) If possible, fasting values will be measured (after fasting for 10 hours or longer).

(d) To be performed within 1 week before the start of treatment (otherwise, on the day as near as possible prior to the start of treatment) so that test results can be checked at the time of start of treatment.

*: Performed before administration of the antihypertensive medication at Week 0.

**: Performed when considered necessary by the primary investigator or investigator (e.g., in case the safety of treatment with azilsartan 20 mg or telmisartan 40 mg must be confirmed such as when the patient has concomitant mild renal/hepatic disease or shows significant increase/decrease in blood pressure)

***: Performed as far as possible.

Appendix B Responsibilities of the Investigator

1. Appropriately conduct the study in compliance with this study protocol and the Ethical Guidelines for Clinical Research and with the highest respect for human rights, safety, and welfare of subjects.
2. Prepare the informed consent form and the information sheet for subjects and revise them if necessary.
3. Confirm the details of the study contracts.
4. Provide the investigators or other site staff with sufficient information regarding the protocol and individual study activities, and instruct and supervise them.
5. Select candidate subjects who meet the purpose of this study protocol, explain to them about the study using explanatory documents, and obtain written consent from each subject.
6. Take responsibility for all study-related medical judgments.
7. Ensure that subjects are provided with sufficient medical treatment for all study-related clinically significant PTEs or AEs throughout the duration of subject's participation in the study and after the study, together with the director of the institution.
8. Submit an expedited report of a serious PTE or AE to the director of the investigational site as soon as possible, as well as notify the directors of other investigational sites participating in this study via the sponsor.
9. Prepare accurate and complete CRFs.
10. Discuss with the sponsor when protocol revision, etc. is proposed by the sponsor.
11. Submit a notification on the completion of the study to the director of the institution when the study is completed.

Appendix C Detailed Description of Amendments to Text

Section	1st version	2nd version	Reason for Amendment
Page 8<br>2.0 Study Summary Planned Number of Subjects (as randomized)	Azilsartan group: 50 subjects to receive azilsartan 20 mg<br>Telmisartan group: 50 subjects to receive telmisartan 40 mg	Azilsartan group: **20** subjects to receive azilsartan 20 mg<br>Telmisartan group: **20** subjects to receive telmisartan 40 mg	To consider for feasibility.
Page 19<br>6.1 Study Design Planned Number of Subjects (as randomized)	Azilsartan group: 50 subjects to receive azilsartan 20 mg<br>Telmisartan group: 50 subjects to receive telmisartan 40 mg	Azilsartan group: **20** subjects to receive azilsartan 20 mg<br>Telmisartan group: **20** subjects to receive telmisartan 40 mg	To consider for feasibility.
Page 30<br>9.1.1 Informed consent	After the conduct of the study is approved at each investigational site, subjects who meet all of the inclusion criteria and none of the exclusion criteria will be provided with an explanation using the informed consent form/information sheet for subjects and registered on the electronic CRF (eCRF) system in order of signing of the informed consent form to start observation. Discontinuation of prior antihypertensives in a subject to be enrolled in the study can be done only after having obtained the informed consent from the subject. Enrollment of new subjects at each site will be stopped when the planned number of subjects for the site is reached. Overall enrollment will be completed when the total number of randomized subjects reaches the planned sample size for the whole study (n = *50* per group, *100* in total).	After the conduct of the study is approved at each investigational site, subjects who meet all of the inclusion criteria and none of the exclusion criteria will be provided with an explanation using the informed consent form/information sheet for subjects and registered on the electronic CRF (eCRF) system in order of signing of the informed consent form to start observation. Discontinuation of prior antihypertensives in a subject to be enrolled in the study can be done only after having obtained the informed consent from the subject. Enrollment of new subjects at each site will be stopped when the planned number of subjects for the site is reached. Overall enrollment will be completed when the total number of randomized subjects reaches the planned sample size for the whole study (n = **20** per group, **40** in total).	To consider for feasibility.
Page 50<br>13.3 Determination of sample size	Planned number of randomized subjects<br>Azilsartan 20 mg group: *50* subjects<br>Telmisartan 40 mg group: *50* subjects<br>[Sample size justification]<br>The above sample size has been selected, with consideration to feasibility, as the number of subjects used to explore the effects of azilsartan 20 mg or telmisartan 40 mg on insulin resistance. This selection was not based on a statistical rationale.	Planned number of randomized subjects<br>Azilsartan 20 mg group: **20** subjects<br>Telmisartan 40 mg group: **20** subjects<br>[Sample size justification]<br>The above sample size has been selected, with consideration to feasibility, as the number of subjects used to explore the effects of azilsartan 20 mg or telmisartan 40 mg on insulin resistance. This selection was not based on a statistical rationale.	To consider for feasibility.
