# Supplementary material for: Effects of azilsartan compared with telmisartan on insulin resistance in patients with essential hypertension and type 2 diabetes mellitus: An open-label, randomized clinical trial
Source: PLoS One. 2019 Apr 3;14(4):e0214727. doi: 10.1371/journal.pone.0214727 (PMC6447197; doi:10.1371/journal.pone.0214727)
Supplement: S2 Protocol — (PDF) [file pone.0214727.s012.pdf]

企業主導臨床研究実施計画書

2 型糖尿病を合併する本態性高血圧症患者における アジルサルタンのインスリン抵抗性に対する効果の探索的検討

A study to explore the effects of Azilsartan compared to Telmisartan on insulin resistance of patients with essential hypertension on type 2 diabetes mellitus by HOMA-R (AT-HOMA)

**研究依頼者**	武田薬品工業株式会社
**臨床研究実施計画書番号**	279/NRP-001
**版数**	第 2 版
**被験薬**	アジルサルタン
**作成日**	2015 年 10 月 20 日

改訂履歴

日付	改訂番号	適用対象
2014 年 1 月 31 日	初版	全臨床研究機関
2015 年 10 月 20 日	第 2 版	全臨床研究機関

1.0 研究管理情報及び研究の原則

1.1 連絡先及び研究関連業務の責任

別紙参照

1.2 研究の原則

本研究は、本臨床研究実施計画書及び以下の要件を遵守し、参加者個人に配慮して行うものとする。

- ヘルシンキ宣言に基づく倫理的原則
- 臨床研究に関する倫理指針（厚生労働省　平成 20 年改正版）
- 適用されるすべての法令及び規則（データ機密保持に関する法令、利益相反ガイドライン等）

目次

1.0 研究管理情報及び研究の原則 3
1.1 連絡先及び研究関連業務の責任 3
1.2 研究の原則 3
2.0 要約 8
3.0 略語 15
4.0 はじめに 16
4.1 背景 16
4.2 本研究を計画した根拠 16
5.0 研究の目的及び評価項目 17
5.1 目的 17
5.2 評価項目 17
5.2.1 主要評価項目 17
5.2.2 副次評価項目 17
5.2.3 その他の評価項目 17
6.0 研究のデザイン 19
6.1 研究デザイン 19
6.2 研究デザイン、投与量、投与期間、計画被験者数の設定根拠 20
6.3 研究全体の中止又は臨床研究機関での研究中止 21
6.3.1 研究全体の中止基準 21
6.3.2 臨床研究機関での研究中止の基準 21
6.3.3 研究全体又は臨床研究機関での研究中断及び中止に関する手順 ... 21
6.4 臨床研究実施計画書の改訂の手続き 21
7.0 被験者の選定及び中止の基準 22
7.1 選択基準 22
7.2 除外基準 22
7.3 併用禁止薬及び併用禁止治療 24
7.4 被験者の管理 25
7.5 被験者ごとの中止基準 26
7.6 被験者の研究中止の手続き 27
8.0 治療 28
8.1 被験薬又は対照薬による治療 28

8.1.1 投与量及び投与方法 28
8.1.2 過量投与 28
8.2 経口血糖降下薬の治療 28
8.3 被験薬、対照薬及び経口血糖降下薬以外の治療 28
8.4 割付表作成と保管 28
8.5 割付及び投与手順 28
9.0 研究実施計画 30
9.1 研究実施手順 30
9.1.1 同意取得 30
9.1.2 人口統計学的データ、既往歴及び前治療薬 30
9.1.3 診察 30
9.1.4 体重、身長及び BMI 31
9.1.5 併用薬 31
9.1.6 合併症 31
9.1.7 バイタルサイン 31
9.1.8 家庭血圧 32
9.1.9 臨床検査 32
9.1.10 妊娠 32
9.1.11 割付 33
9.1.12 無作為化前に中止した症例の記録 33
9.2 被験者の服薬状況 33
9.3 検査・観察項目の実施時期 34
9.3.1 治療期開始前 34
9.3.2 治療期 34
9.4 生体試料の使用方法と保存、廃棄 36
10.0 PRETREATMENT EVENT 及び有害事象 37
10.1 定義 37
10.1.1 Pretreatment Event 37
10.1.2 有害事象 37
10.1.3 Pretreatment Event 及び有害事象に関して考慮すべき事項 37
10.1.4 重篤な有害事象 39
10.1.5 有害事象等の程度 39

10.1.6 有害事象の因果関係 40
10.1.7 研究手順との因果関係 40
10.1.8 発現日 40
10.1.9 消失日 41
10.1.10被験薬又は対照薬に関する処置 41
10.1.11.. 転帰 41
10.2 手順 42
10.2.1 有害事象等の収集及び報告 42
10.2.2 重篤な有害事象の収集及び報告 43
10.2.3 重篤な有害事象の臨床研究機関への周知 43
10.3 重篤な有害事象の追跡調査 44
11.0 本研究のために設置された委員会 45
12.0 データマネジメント及び記録の保存 46
12.1 症例報告書 46
12.2 記録の保存 46
13.0 統計手法 47
13.1 統計及び解析計画 47
13.1.1 解析対象集団 47
13.1.2 人口統計学的及び他の基準値の特性の解析 47
13.1.3 有効性の解析 47
13.1.4 安全性の解析 49
13.2 中間解析及び早期中止に関する基準 50
13.3 計画被験者数の決定 50
14.0 品質管理及び品質保証 51
14.1 臨床研究機関へのモニタリング 51
14.2 臨床研究に関する倫理指針及び臨床研究実施計画書からの逸脱 51
14.3 品質保証及び規制当局による調査 51
15.0 研究の倫理的な実施 52
15.1 利益相反 52
15.2 倫理審査委員会等の承認 52
15.3 同意・説明文書及び被験者の同意 52
15.4 被験者の個人情報等の保護 53

15.5 被験者の利益及び不利益 53
15.5.1 被験者の利益 53
15.5.2 被験者の不利益 53
15.6 研究成績の帰属、アクセス権及び公表の手順 53
15.6.1 研究成績の帰属 53
15.6.2 データのアクセス権 53
15.6.3 研究成績の公表の手順 54
15.7 臨床研究実施計画の事前登録及び研究成績の開示 54
15.7.1 臨床研究実施計画の事前登録 54
15.7.2 研究成績の開示 54
15.8 健康被害に対する補償 54
16.0 **参考文献** 55

付録一覧

付録 A **研究スケジュール** 56
付録 B **研究責任者の責務** 57
付録 C **改訂内容** 58

2.0　要約

研究依頼者：

武田薬品工業株式会社

被験薬：

アジルサルタン（商品名：アジルバ）

課題名：

2 型糖尿病を合併する本態性高血圧症患者におけるアジルサルタンのインスリン抵抗性に対する効果の探索的検討

臨床研究実施計画書番号：

279/NRP-001

研究デザイン：

本研究は、多施設共同、無作為化、非盲検、並行群間比較の探索的試験である。

目的：

2 型糖尿病を合併する本態性高血圧症患者を対象として、アジルサルタン 20 mg を 1 日 1 回 12 週間経口投与したときのインスリン抵抗性に対する効果について、テルミサルタン 40 mg を対照に探索的に検討する。

対象：

2 型糖尿病を合併する本態性高血圧症患者

計画被験者数：

無作為化された被験者数として
アジルサルタン 20 mg 群：20 例
テルミサルタン 40 mg 群：20 例

臨床研究機関数：

約 10 施設

用法用量：

アジルサルタン 20 mg 又はテルミサルタン 40 mg を 1 日 1 回、朝食前又は朝食後の午前中に服薬する。ただし、治療期の規定の来院時は降圧薬を服薬せずに来院し、所定の検査が終了してから降圧薬を服薬する。

投与経路：

経口

治療期及び来院回数：

研究デザインの概略図を下記に示す。

治療期：12 週間

来院回数：4 回

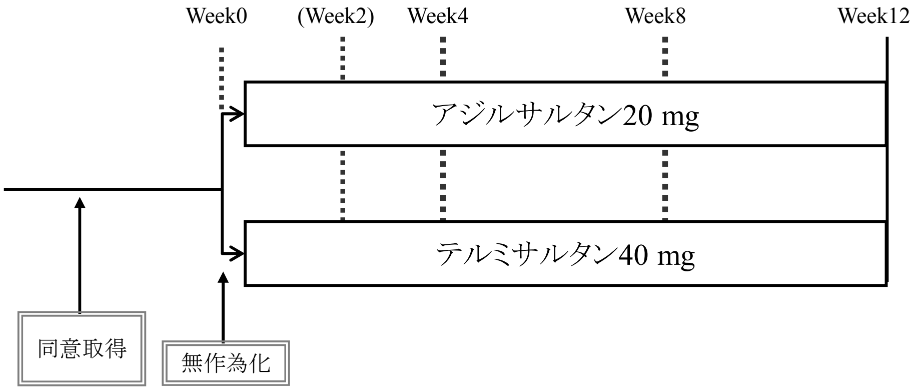

主な選択基準：

1. Ⅰ度・Ⅱ度の本態性高血圧症と診断され、研究責任者又は研究者がアジルサルタン 20mg 及びテルミサルタン 40mg による治療が適切と判断した者
2. 治療期開始時（Week0）の座位収縮期血圧*が 130 mmHg 以上 180 mmHg 未満又は、座位拡張期血圧*が 80 mmHg 以上 110 mmHg 未満の者

   *：5 分間以上、座位の姿勢で安静を保った後に、連続した 2 回の測定値が安定した値（測定値の差が拡張期血圧は 5mmHg 未満、収縮期血圧は 10 mmHg 未満）を示した時点で測定をやめ、最後の連続した 2 回の平均値（小数点第 1 位を切り捨てる）を用いる。
3. 2 型糖尿病の者
4. 同意取得前 3 ヵ月間の HbA1c（NGSP 値）が 8.4%未満かつ、同意取得前 3 ヵ月間の HbA1c の変動（最高値と最低値の差）が 0.3%以内の者
5. 生活習慣（食事・運動療法等）の改善指導がされており、食事・運動療法を実施している場合には、同意取得前 3 ヵ月間において一定の方法で実施している者
6. 同意取得時の年齢が 20 歳以上である者
7. 観察期間中の外来通院が可能な者
8. 同意のための意思能力があり、本研究の参加に先立って本人の文書による同意が得られる者

主な除外基準：

1. Ⅲ度本態性高血圧症（座位収縮期血圧 180 mmHg 以上又は座位拡張期血圧 110 mmHg 以上）、二次性高血圧症、悪性高血圧症の者
2. 降圧薬を服薬しているⅡ度本態性高血圧症（座位収縮期血圧 160 mmHg 以上又は座位拡張期血圧 100 mmHg 以上）の者
3. 治療期開始前 2 週間以内に降圧薬を服薬した者

   ただし、同意取得時に降圧薬を服薬している被験者を組み入れる場合は、同意取得後に 2 週間休薬することで本研究に組み入れることを可とする。
4. 治療期開始前 3 ヵ月以内に RAS 阻害薬又はチアゾリジン薬を服薬した者
5. 1 型糖尿病の者
6. 治療期開始時（Week0）の空腹時血糖が 180mg/dL 以上又は HOMA-R が 1.6 以下の者
7. 同意取得時に下記の薬剤による治療を受けている又は下記の薬剤による治療が必要な者

   ・インスリン及びグルカゴン様ペプチド-1（以下、GLP-1）受容体作動薬を含む経口以外の血糖降下薬
   ・3 剤以上の経口血糖降下薬

8. 治療期開始前 3 ヵ月以内に糖尿病治療薬の変更（用法用量の変更も含む）をした者

9. 治療期開始前 3 ヵ月以内に下記の循環器関連疾患の診断・治療をされた者

・心疾患：心筋梗塞、冠動脈・血行再建術

・脳血管障害：脳梗塞・脳出血、一過性脳虚血発作

・進行した高血圧性網膜症（出血又は滲出・乳頭浮腫）

10. 治療期開始前 3 ヵ月以前に下記の循環器関連疾患の診断・治療をされ、現在も状態が安定しない者

・心疾患：心筋梗塞、冠動脈・血行再建術

・脳血管障害：脳梗塞・脳出血、一過性脳虚血発作

11. 下記の循環器関連疾患の既往又は合併のある者

・弁狭窄

・加療を要する狭心症

・加療を要するうっ血性心不全

・加療を要する不整脈（発作性心房細動、高度の徐脈、心室頻拍、心室細動等）

・間欠性跛行等の症状を伴う閉塞性動脈硬化症

12. 重症ケトーシス、糖尿病性昏睡又は前昏睡、重症感染症、重篤な外傷のある者

13. 臨床的に明らかな腎障害（eGFR が 30 mL/min/1.73 ㎡未満等）のある者

14. 胆汁の分泌が極めて悪い又は重篤な肝障害のある者

15. アジルサルタン若しくはテルミサルタン又はその両者に対して過敏症又はアレルギーの既往がある者

16. 高カリウム血症（臨床検査値で 5.5mEq/L 以上）の者

17. 他の臨床試験に参加している者

18. 妊婦又は妊娠している可能性のある女性、及び授乳中の女性

19. その他、研究責任者又は研究者が対象として不適切と判断した者

評価項目：

＜主要評価項目＞

インスリン抵抗性指数（HOMA-R*）

・治療期終了時点における治療期開始時点からの変化量

*：HOMA-R=空腹時インスリン値（μU/mL）×空腹時血糖値（mg/dL）/405

＜副次評価項目＞

(1) 有効性：

① 空腹時血糖値の変化量

② 空腹時インスリン値の変化量

③ HbA1c の変化量

④ HOMA-βの変化量

HOMA-β=空腹時インスリン値（μU/mL）×360/{空腹時血糖値（mg/dL）-63}

⑤ 1,5-AG の変化量

(2) 安全性：有害事象

＜その他の評価項目＞

① 診察時血圧値の変化量

② 家庭血圧計を用いた早朝血圧値の測定値

③ 尿素窒素（以下、BUN）値の変化量

④ 血清クレアチニン値の変化量

⑤ 総コレステロール値の変化率

⑥ HDL 値の変化率

⑦ LDL 値の変化率

⑧ 空腹時トリグリセリド値の変化率

⑨ 高分子アディポネクチン値の変化量

⑩ 血漿アルドステロンの変化量

⑪ 血漿レニン活性の変化量

⑫ 高感度 C 反応性蛋白（以下、CRP）の変化量

⑬ 尿中アルブミン／クレアチニン比の変化量

尿中アルブミン／クレアチニン比（mg/g・Cr）＝尿中アルブミン（mg/L）×100／尿中クレアチニン（mg/dL）

⑭ 尿中 Na／クレアチニン比の変化量

尿中 Na／クレアチニン比（g/day）＝尿中 Na（mEq/L）×2.3／尿中クレアチニン（mg/dL）

統計手法：

(1) 解析対象集団

本研究では、解析対象集団として「最大の解析対象集団」及び「安全性データの解析対象集団」の 2 種類の解析対象集団を設ける。

「最大の解析対象集団」は、以下の基準を満たす登録被験者の集団として定義する。

・無作為化され、本研究中に被験薬又は対照薬を少なくとも 1 回以上投与された被験者

「安全性データの解析対象集団」は、以下の基準を満たす登録被験者の集団として定義する。

・本研究中に被験薬又は対照薬を少なくとも 1 回以上投与された被験者

(2)　有効性の解析

1.　主要評価項目

〔主要評価項目〕

・インスリン抵抗性指数（HOMA-R*）

治療期終了時点における治療期開始時点からの変化量

*：HOMA-R=空腹時インスリン値（μU/mL）×空腹時血糖値（mg/dL）/405

〔解析方法〕

1)　主解析

「最大の解析対象集団」を対象として、主要評価項目について、投与群別に要約統計量を算出し、平均値の投与群間差（アジルサルタン群－テルミサルタン群）の点推定値及びその両側 95%信頼区間を算出する。

2)　副解析

「最大の解析対象集団」を対象として、主要評価項目について、治療期開始時点の HOMA-R（2.5 未満、2.5 以上）、服薬中のビグアナイド薬（有、無）を層別因子とした投与群別の要約統計量を算出する。「最大の解析対象集団」を対象として、主要評価項目について、投与群別に要約統計量を算出し、平均値の投与群間差（アジルサルタン群－テルミサルタン群）の点推定値及びその両側 95%信頼区間を算出する。治療期開始時点の HOMA-R（2.5 未満、2.5 以上）、服薬中のビグアナイド薬（有、無）、治療期開始時点の HOMA-R 値（2.5 未満、2.5 以上）を層別因子とした投与群別の要約統計量を集計する。

2.　副次評価項目

「最大の解析対象集団」を対象として、以下の項目について、投与群別に要約統計量を算出する。

・空腹時血糖値の変化量

治療期終了時点における治療期開始時点からの変化量

・空腹時インスリン値の変化量

治療期終了時点における治療期開始時点からの変化量

・HbA1c の変化量

治療期終了時点における治療期開始時点からの変化量

・HOMA-β*

治療期終了時点における治療期開始時点からの変化量

*：HOMA-β=空腹時インスリン値（μU/mL）×360/｛空腹時血糖値（mg/dL）-63｝

・1,5-AG の変化量

治療期終了時点における治療期開始時点からの変化量

3.　その他の評価項目

「最大の解析対象集団」を対象として、以下の項目について、投与群別に要約統計量を算出する。

・診察時血圧値の変化量

治療期終了時点における治療期開始時点からの変化量

・家庭血圧計を用いた早朝血圧値の測定値

治療期終了時点における測定値

・BUN 値の変化量

治療期終了時点における治療期開始時点からの変化量

・血清クレアチニン値の変化量

治療期終了時点における治療期開始時点からの変化量

・総コレステロール値の変化率

治療期終了時点における治療期開始時点からの変化率

・HDL 値の変化率

治療期終了時点における治療期開始時点からの変化率

・LDL 値の変化率

治療期終了時点における治療期開始時点からの変化率

・空腹時トリグリセリド値の変化率

治療期終了時点における治療期開始時点からの変化率

・高分子アディポネクチン値の変化量

治療期終了時点における治療期開始時点からの変化量

・血漿アルドステロンの変化量

治療期終了時点における治療期開始時点からの変化量

・血漿レニン活性の変化量

治療期終了時点における治療期開始時点からの変化量

・高感度 CRP の変化量

治療期終了時点における治療期開始時点からの変化量

・尿中アルブミン／クレアチニン比の変化量

治療期終了時点おける治療期開始時点からの変化量

・尿中 Na／クレアチニン比の変化量

治療期終了時点おける治療期開始時点からの変化量

(3) 安全性の解析

・有害事象

有害事象は MedDRA を使用して読み替える。以下に示した有害事象について、器官別大分類（System Organ Class; SOC）かつ基本語（Preferred Term; PT）別の頻度集計を投与群別に行う。

・全ての有害事象<br>・程度別の有害事象<br>・被験薬又は対照薬との因果関係が否定できない有害事象<br>・程度別の被験薬又は対照薬との因果関係が否定できない有害事象<br>・投与中止に至った有害事象<br>・重篤な有害事象
**計画被験者数の設定根拠**：<br>アジルサルタン 20mg 及びテルミサルタン 40mg のインスリン抵抗性に対する影響について探索的に検討する被験者数として、実施可能性を考慮して設定した。本設定は、統計学的根拠に基づいたものではない。

3.0 略語

AE	adverse event	有害事象
ACE	angiotensin converting enzyme	アンジオテンシン変換酵素
1,5-AG	1,5-anhydroglucitol	1,5-アンヒドログルシトール
ARB	angiotensin Ⅱ receptor blocker	アンジオテンシンⅡ受容体拮抗薬
BMI	body mass index	体容量指数
BUN	Blood urea nitrogen	尿素窒素
COI	conflict of interest	利益相反
CRP	C-reactive protein	C 反応性蛋白
EDC	electronic data capture	電子症例報告書システム
eGFR	estimate glomerular filtration rate	推定糸球体濾過量
FDA	food and drug administration	米国食品医薬品局
GCP	good clinical practice	医薬品の臨床試験の実施に関する基準
GLP-1	Glucagon-like peptide-1	グルカゴン様ペプチド-1
γ-GTP	γ-glutamyl transpeptidase	ガンマグルタミルトランスペプチターゼ
GOT	glutamic oxaloacetic transaminase	グルタミン酸オキサロ酢酸トランスアミナーゼ
GPT	glutamic pyruvic transaminase	グルタミン酸ピルビン酸トランスアミナーゼ
HbA1C	Hemoglobin A1C	ヘモグロビン A1C
HDL	High density lipoprotein	HDL
HOMA-R	homeostasis model assessment ratio	インスリン抵抗性指数
HOMA-β	homeostasis model assessment beta cell	インスリン分泌能
LDL	Low density lipoprotein	LDL
MedDRA	medical dictionary for regulatory activities	医薬品規制用語集
MHRA	medicines and healthcare products regulatory agency	英国医薬品規制当局
PPARγ	peroxisome proliferator-activated receptor γ	ペルオキシソーム増殖因子活性化受容体γ
PT	Preferred Term	基本語
RAS	Renin-Angiotensin System	レニン・アンジオテンシン系
SAE	serious adverse event	重篤な有害事象
SAP	statistical analysis plan	統計解析計画書
SOC	System Organ Class	器官別大分類
TNF-α	tumor necrosis factor	腫瘍壊死因子-α
WHO	world health organization	世界保健機関

4.0 はじめに

4.1 背景

日本高血圧学会の「高血圧治療ガイドライン 2009 [1)] 」では、高血圧症と 2 型糖尿病はいずれも動脈硬化による大血管障害の重要な危険因子であり、両者を合併すると脳血管障害や虚血性心疾患の発症頻度が大きく増加するため、血圧及び血糖の厳格な管理が重要であるとしている。

高血圧症と 2 型糖尿病は、インスリン抵抗性状態を共通の背景因子としたメタボリックシンドロームの主要因子であるため、両者を合併しているときの降圧治療では、降圧効果のみならず、インスリン感受性、糖代謝や脂質代謝等についても十分な配慮が必要となる。そのため、「高血圧治療ガイドライン 2009」及び「糖尿病治療ガイド 2012-2013 [2)]」では、インスリン感受性を改善し糖尿病の新規発症抑制に優れ、脂質代謝に影響を及ぼさないアンジオテンシンⅡ受容体拮抗薬（angiotensinⅡreceptor blocker（以下、「ARB」））やアンジオテンシン変換酵素（angiotensin converting enzyme）阻害薬（以下、「ACE 阻害薬」）を第一選択薬として推奨している。

また一方で、脂肪細胞が産生する生理活性物質であるアディポネクチンは膵臓から分泌されるインスリンへの感受性を亢進する作用を有しており、脂肪組織に特異的に発現しているペルオキシソーム増殖因子活性化受容体γ（Peroxisome Proliferator-Activated Receptor γ（以下、「PPAR γ」））が活性化されることによりアディポネクチンの産生が促進され、インスリン抵抗性が改善されるといわれている。

テルミサルタンは、優れた降圧効果を示す ARB で、非臨床試験において PPARγ活性化作用を介した有用性 [3)]が認められていることから、臨床現場においては、インスリン抵抗性の改善を始めとした糖尿病に対する効果 [4)]を期待して処方されている。

また、新規 ARB であるアジルサルタンは、他の ARB と比較しても臨床的に強力な降圧作用を示すとともに、非臨床試験において PPARγの発現量を増加し、インスリンに対する感受性を低下させる炎症性サイトカインの腫瘍壊死因子-α（Tumor Necrosis Factor （以下、「TNF-α」））の発現量を低下させることが認められており [5)]、臨床においてもインスリン抵抗性指数（homeostasis model assessment ratio （以下、「HOMA-R」））等を指標としたインスリン抵抗性を改善する効果 [6)]が期待される。

しかしながら、ARB の HOMA-R 等の糖尿病関連指標に対する効果については、臨床試験で比較検討した成績は十分とはいえず、一定の評価には至っていない。そのため、今回我々の研究では、2 型糖尿病を合併している本態性高血圧症患者に対して、アジルサルタンを投与したときの HOMA-R 等の糖尿病関連指標等に対する効果について、テルミサルタンを対照に探索的に検討することとした。

4.2 本研究を計画した根拠

本研究は、2 型糖尿病を合併するⅠ度・Ⅱ度本態性高血圧症患者を対象に、アジルサルタン又はテルミサルタン投与時のインスリン抵抗性に対する効果を探索的に検討することで高血圧治療の発展に寄与するために計画した。

5.0　研究の目的及び評価項目

5.1　目的

2 型糖尿病を合併するⅠ度・Ⅱ度本態性高血圧症患者を対象に、アジルサルタン 20 mg を 1 日 1 回 12 週間経口投与したときのインスリン抵抗性に対する効果について、テルミサルタン 40 mg を対照に探索的に検討する。

5.2　評価項目

5.2.1　主要評価項目

インスリン抵抗性指数（HOMA-R*）
・治療期終了時点における治療期開始時点からの変化量
*：HOMA-R=空腹時インスリン値（μU/mL）×空腹時血糖値（mg/dL）/405

5.2.2　副次評価項目

(1)　有効性：

①　空腹時血糖値の変化量

②　空腹時インスリン値の変化量

③　HbA1c（NGSP 値）の変化量

④　HOMA-β*の変化量

*：HOMA-β=空腹時インスリン値（μU/mL）×360/｛空腹時血糖値（mg/dL）-63｝

⑤　1,5-AG の変化量

(2)　安全性：

有害事象

5.2.3　その他の評価項目

(1)　有効性：

①　診察時血圧値の変化量

②　家庭血圧計を用いた早朝血圧値の測定値

③　尿素窒素（以下、BUN）値の変化量

④　血清クレアチニン値の変化量

⑤　総コレステロール値の変化率

⑥　HDL 値の変化率

⑦　LDL 値の変化率

⑧　空腹時トリグリセリド値の変化率

⑨　高分子アディポネクチン値の変化量

⑩　血漿アルドステロンの変化量

⑪　血漿レニン活性の変化量

⑫　高感度 C 反応性蛋白（以下、CRP）の変化量

⑬　尿中アルブミン／クレアチニン比の変化量

尿中アルブミン／クレアチニン比（mg/g・Cr）＝尿中アルブミン（mg/L）×100／尿中クレアチニン（mg/dL）

⑭　尿中 Na／クレアチニン比の変化量

尿中 Na／クレアチニン比（g/day）＝尿中 Na（mEq/L）×2.3／尿中クレアチニン（mg/dL）

6.0 研究のデザイン

6.1 研究デザイン

（1） 研究デザイン

本研究は、多施設共同、無作為化、非盲検、並行群間比較の探索的試験である。

（2） 被験者の組み入れ

研究責任者及び研究責任者とともに臨床研究を実施する者（以下、「研究者」）は、被験者からの同意取得後に治療期開始時の検査を実施する。

同意取得時に降圧薬を服薬している被験者を組み入れるときには、同意取得後に降圧薬を 2 週間休薬する。

（3） 治療

適格性判定の結果、適格と判定された被験者は、治療期開始時点の HOMA-R（2.5 未満、2.5 以上）、服薬中の経口血糖降下薬（ビグアナイド薬の有無）を割付因子として、アジルサルタン 20 mg 群又はテルミサルタン 40 mg 群に 1:1 に無作為に割り付けられる。

研究責任者及び研究者は、登録センターから通知された割付結果をもとにアジルサルタン 20mg 又はテルミサルタン 40mg を処方する。

被験者は、アジルサルタン 20 mg 又はテルミサルタン 40 mg を 12 週間、1 日 1 回朝食前又は朝食後の午前中に服薬する。

（4） 計画被験者数

無作為化された被験者数として
アジルサルタン 20 mg 群：20 例
テルミサルタン 40 mg 群：20 例

（5） 臨床研究機関数

約 10 施設

（6） 被験者の治療期及び来院回数

研究デザインの概略図を図 6.a に示す。

治療期：12 週間

来院回数：4 回

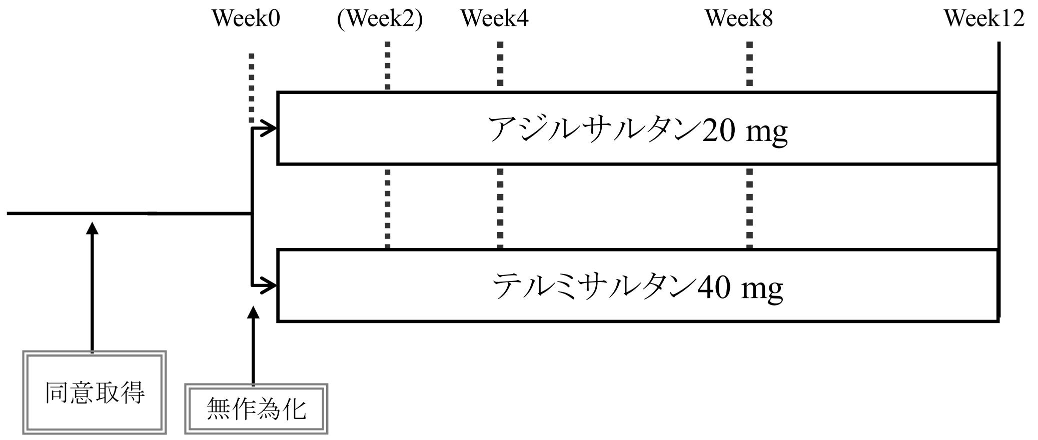

図 6.a　研究デザインの概略図

6.2　研究デザイン、投与量、投与期間、計画被験者数の設定根拠

（1）　研究デザインの設定根拠

本研究は、2 型糖尿病を合併するⅠ度・Ⅱ度本態性高血圧症患者におけるアジルサルタン 20 mg 及びテルミサルタン 40 mg 投与時のインスリン抵抗性に対する効果を客観的に評価するため、治療期開始時点の HOMA-R（2.5 未満、2.5 以上）、糖尿病治療薬（ビグアナイド薬の有無）を割付因子とした無作為層別割付二群間比較法を採用した。

アジルサルタンの HOMA-R 等の糖尿病関連指標等に対する効果を探索的に検討するために、ARB の中で非臨床試験において PPARγ 活性化作用が認められ [3]、臨床現場において糖尿病に対する効果 [4] を期待して使用されているテルミサルタンを対照群として設定した。

また、前治療薬（降圧薬）の影響を排除するために、同意取得時に降圧薬を服薬している被験者においては、2 週間の休薬期間を設定した。

（2）　投与量の設定根拠

通常の臨床用量での評価をするために、アジルサルタン 20 mg、テルミサルタン 40 mg を投与量として設定した。

（3）　投与期間の設定根拠

投与期間は、これまでにテルミサルタンのインスリン抵抗性の改善作用を 12 週間投与で検討されていること [3] 及び、アジルサルタン及びテルミサルタンの開発時のデータをもとに、両剤の降圧効果が安定するのに 8～12 週間要することを踏まえ、12 週間と設定した。

（4）　計画被験者数の設定根拠

13.3 項参照

6.3 研究全体の中止又は臨床研究機関での研究中止

6.3.1 研究全体の中止基準

研究依頼者は、研究の主目的の達成又は被験者の安全性を危うくする重大な倫理指針違反があった場合は、研究を速やかに中止する。

6.3.2 臨床研究機関での研究中止の基準

臨床研究機関、研究責任者及び研究者が倫理指針、臨床研究実施計画書又は契約書に重大な違反を犯したことが判明した場合、又は適正な研究の実施ができなくなった場合、当該臨床研究機関は研究依頼者から研究の中止を求められることがある。

6.3.3 研究全体又は臨床研究機関での研究中断及び中止に関する手順

研究依頼者又は倫理審査委員会等の臨床研究機関の委員会が、研究全体又はある臨床研究機関での研究の中断又は中止を決定した場合、当該研究に対して定められた手順が研究依頼者によって指示される。研究の中断又は中止に際して、当該臨床研究機関はその手順に従うものとする。

6.4 臨床研究実施計画書の改訂の手続き

臨床研究実施計画書を改訂する必要が生じた場合には、その可否について、研究依頼者が検討し、決定する。

臨床研究実施計画書の改訂内容は、全ての臨床研究機関の研究責任者に通知される。

本通知を受け、各臨床研究機関の研究責任者は、各臨床研究機関の規定に従い、必要に応じて再度倫理審査委員会の審査を受け、臨床研究機関の長の承認を得なければならない。

7.0 被験者の選定及び中止の基準

7.1 選択基準

以下の基準の全てを満たす被験者を本研究の対象とする。

1. Ⅰ度・Ⅱ度の本態性高血圧症と診断され、研究責任者又は研究者がアジルサルタン 20mg 及びテルミサルタン 40mg による治療が適切と判断した者
2. 治療期開始時（Week0）の座位収縮期血圧*が 130 mmHg 以上 180 mmHg 未満又は、座位拡張期血圧*が 80 mmHg 以上 110 mmHg 未満の者

   *：5 分間以上、座位の姿勢で安静を保った後に、連続した 2 回の測定値が安定した値（測定値の差が拡張期血圧は 5mmHg 未満、収縮期血圧は 10 mmHg 未満）を示した時点で測定をやめ、最後の連続した 2 回の平均値（小数点第 1 位を切り捨てる）を用いる。
3. 2 型糖尿病の者
4. 同意取得前 3 ヵ月間の HbA1c（NGSP 値）が 8.4%未満かつ、同意取得前 3 ヵ月間の HbA1c の変動（最高値と最低値の差）が 0.3%以内の者
5. 生活習慣（食事・運動療法等）の改善指導がされており、食事・運動療法を実施している場合には、同意取得前 3 ヵ月間において一定の方法で実施している者
6. 同意取得時の年齢が 20 歳以上である者
7. 観察期間中の外来通院が可能な者
8. 同意のための意思能力があり、本研究の参加に先立って本人の文書による同意が得られる者

【選択基準の設定根拠】

1.-3. 本研究の対象疾患として設定した。

4. 血糖コントロールが不十分な被験者を除外するため、被験者の安全性に配慮して上限値を 8.4%（NGSP 値）と設定した。また、血糖コントロールが安定しない被験者を除外するため、被験者の安全性に配慮して HbA1c の変動値を設定した。
5. 食事・運動療法の変化が評価に影響を与える可能性を考慮し設定した。
6. 本研究に参加することについて本人の意思による判断が可能な年齢の下限として 20 歳を規定し、可能な範囲でより多くの集団の成績を集積する目的から年齢の上限は規定しなかった。
7. 入院での血圧評価は、日常生活での活動時と比べ血圧値に乖離が生じる可能性が高いことから、評価への影響を考慮して外来と設定した。
8. 研究を実施するにあたっての基本的事項として設定した。

7.2 除外基準

以下の基準のいずれかに該当する被験者は本研究の対象としない。

1. Ⅲ度本態性高血圧症（座位収縮期血圧 180 mmHg 以上又は座位拡張期血圧 110 mmHg 以上）、二次性高血圧症、悪性高血圧症の者

2. 降圧薬を服薬しているⅡ度の本態性高血圧症（座位収縮期血圧 160mmHg 以上又は座位拡張期血圧 100 mmHg 以上）の者

3. 治療期開始前 2 週間以内に降圧薬を服薬した者

   ただし、同意取得時に降圧薬を服薬している被験者を組み入れる場合は、同意取得後に 2 週間休薬することで本研究に組み入れることを可とする。

4. 治療期開始前 3 ヵ月以内に RAS 阻害薬又はチアゾリジン薬を服薬した者

5. 1 型糖尿病の者

6. 治療期開始時（Week0）の空腹時血糖が 180mg/dL 以上又は HOMA-R が 1.6 以下の者

7. 同意取得時に下記の薬剤による治療を受けている又は下記の薬剤による治療が必要な者

   ・インスリン及びグルカゴン様ペプチド-1（以下、GLP-1）受容体作動薬を含む経口以外の血糖降下薬

   ・3 剤以上の経口血糖降下薬

8. 治療期開始前 3 ヵ月以内に糖尿病治療薬の変更（用法用量の変更も含む）をした者

9. 治療期開始前 3 ヵ月以内に下記の循環器関連疾患の診断・治療をされた者

   ・心疾患：心筋梗塞、冠動脈・血行再建術

   ・脳血管障害：脳梗塞・脳出血、一過性脳虚血発作

   ・進行した高血圧性網膜症（出血又は滲出・乳頭浮腫）

10. 治療期開始前 3 ヵ月以前に下記の循環器関連疾患の診断・治療をされ現在も状態が安定しない者

    ・心疾患：心筋梗塞、冠動脈・血行再建術

    ・脳血管障害：脳梗塞・脳出血、一過性脳虚血発作

11. 下記の循環器関連疾患の既往又は合併のある者

    ・弁狭窄

    ・加療を要する狭心症

    ・加療を要するうっ血性心不全

    ・加療を要する不整脈（発作性心房細動、高度の徐脈、心室頻拍、心室細動等）

    ・間欠性跛行等の症状を伴う閉塞性動脈硬化症

12. 重症ケトーシス、糖尿病性昏睡又は前昏睡、重症感染症、重篤な外傷のある者

13. 臨床的に明らかな腎障害（eGFR が 30 mL/min/1.73 ㎡未満等）のある者

14. 胆汁の分泌が極めて悪い又は重篤な肝障害のある者

15. アジルサルタン若しくはテルミサルタン又はその両者に対して過敏症又はアレルギーの既往がある者

16. 高カリウム血症（臨床検査値で 5.5mEq/L 以上）の者

17. 他の臨床試験に参加している者

18. 妊婦又は妊娠している可能性のある女性、及び授乳中の女性

19. その他、研究責任者又は研究者が対象として不適切と判断した者

【除外基準の設定根拠】

1-6. 本研究の対象として適切でないため設定した。

7-8. 割付けられた降圧薬（アジルサルタン又はテルミサルタン）の薬効を正確に評価するため、設定した。

9-11. 高血圧性合併症への高リスク群となる被験者を除くために、WHO-ISH 2003 Statement on management of hypertension の Factors influencing prognosis における「Associated clinical conditions」を参考に設定した。

12-13. 被験者の安全性確保のため設定した。

14. テルミサルタンの投与禁忌であることから設定した。

15. アジルサルタン及びテルミサルタンは、それぞれの成分に対し過敏症の既往歴のある者は投与禁忌であることから設定した。

16. アジルサルタン及びテルミサルタンではいずれも高カリウム血症が重大な副作用に挙げられているため、被験者の安全性確保のため設定した。

17. 本研究の評価の妥当性を確保するため設定した。

18. アジルサルタン及びテルミサルタンは、妊婦又は妊娠している可能性のある女性は投与禁忌であることから設定した。また、アジルサルタン及びテルミサルタンは、非臨床試験において乳汁中への移行が認められていることから設定した。

19. 研究を実施するにあたっての基本的事項として設定した。

7.3 併用禁止薬及び併用禁止治療

治療期開始時から治療期終了まで、HOMA-R 又は血圧に影響を及ぼす下記の薬剤の使用を禁止する。なお、併用禁止薬以外で従来から使用している合併症に対しての治療薬は継続使用して差し支えないが、既に使用している経口血糖降下薬は、用法・用量の変更は不可とする。

(1) 割付けられた降圧薬以外の降圧薬（カルシウム拮抗薬を含む）

(2) 同意取得時に服薬していた経口血糖降下薬以外の経口血糖降下薬

(3) 糖質コルチコイド製剤（外用薬など局所作用の薬物は除く）

(4) フィブラート系薬剤

(5) エストロゲン製剤（経口避妊薬を含む）

(6) 抗狭心症薬（抗血小板薬（アスピリン等）は除く）

(7) 抗不整脈薬（β遮断薬を含む）

(8) ジギタリス製剤

(9) カリウム補給薬

(10) チアゾリジン薬

(11) インスリン

(12) GLP-1 受容体作動薬

【併用禁止薬の設定根拠】

(1) - (5) 本研究の目的に照らし、割付けられた降圧薬の効果を適切に評価するため設定した。

(6), (7), (9) - (12) 除外基準との整合から設定した。

(8) ARB との併用により、高カリウム血症が発現する可能性があるため設定した。

7.4 被験者の管理

研究責任者及び研究者は、下記の事項に留意又は被験者に指導する。

(1) 割付けられた降圧薬を指示通りに服薬するよう指導する。前回の来院後、規定された服薬量の 50%未満等、被験者の服薬不遵守が判明し、改善が認められない場合には、状況に応じて研究の参加を中止させる。

(2) 併用する経口血糖降下薬がある場合には、用法用量を変更せず、指示通りに服薬するよう指導する。前回の来院後、規定された服薬量の 50%未満等、被験者の服薬不遵守が判明し、改善が認められない場合には、状況に応じて研究への参加を中止させる。

(3) 降圧薬による血圧低下の可能性について被験者に十分説明する。予定来院時以外に自宅などで血圧低下に伴う症状（めまい、立ちくらみなど）がみられた場合には直ちに臥位安静を保ち、それでも改善しないようであれば速やかに研究責任者又は研究者に電話等で報告し、指示を仰ぐか又は来院するよう被験者に指導する。

(4) 予定来院時以外に自宅等で血圧上昇に伴う症状（頭痛、動悸、ほてり、発汗等）がみられた場合には、速やかに研究責任者又は研究者に電話等で報告し指示を仰ぐよう指導する。研究責任者及び研究者は、状況に応じて被験者を来院させる。

(5) 同意取得後の降圧薬が投与されない期間において、家庭血圧を朝晩それぞれ少なくとも 1 回測定し、座位収縮期血圧が 180 mmHg 以上又は座位拡張期血圧 110 mmHg 以上等著しい昇圧がみられた場合には、速やかに研究責任者又は研究者に電話等で報告し指示を仰ぐよう指導する。研究責任者及び研究者は、状況に応じて被験者に来院させる。

(6) 家庭血圧計による血圧測定を実施するときには、2 分間以上、座位の姿勢で安静を保った後に、30 秒以上の間隔で 2 回繰り返し測定するよう指導する。家庭血圧計による血圧測定結果の収集は研究期間中 4 回行われるが、各測定時点では可能な限り同じ状態を保つよう指導する。

(7) 食事療法及び運動療法を実施している場合は、研究責任者又は研究者は指示内容（指示カロリー数、食塩量など）が研究期間を通じて一定となるようにし、また、被験者に対し食事療法及び運動療法を遵守するよう指導する。

(8) 低血糖症状（異常な空腹感、脱力感、手指のふるえ、冷汗、動悸など）がみられた場合にはブドウ糖又はショ糖（砂糖）を摂取し、それでも改善しないようであれば速やかに来院するよう指導する。

(9) 血圧測定前 30 分以内のカフェイン含有物の摂取及び喫煙はしないよう指導する。

(10) 来院日には、当日の午前中の降圧薬を服薬せずに来院するよう指導する。更に、来院ごとに来院日前日及び来院日当日の服薬の有無を報告させる。

(11) 軽度の腎疾患、肝疾患等を合併している又は著しい血圧低下・上昇が認められる等、アジルサルタン 20mg 又はテルミサルタン 40mg にて治療することの安全性を確認する必要があると判断した場合には、治療期 2 週時に来院を設定するなど、慎重に経過観察する。

(12) 妊娠する可能性のある被験者については、適切な避妊を行うよう指導する。妊娠していることが判明した場合には、速やかに報告させ、直ちに研究を中止する。

(13) 研究責任者又は研究者により指示された併用禁止薬を遵守するよう被験者に指導する。研究責任者又は研究者の処方以外の薬を服薬した際には、その内容を報告させる。

(14) 自覚症状・他覚所見について、その内容、発現日、程度、転帰及び転帰日のうち必要事項を来院の際に報告させる。

(15) 臨床検査が予定されている来院日は 10 時間以上の絶食下にて来院するよう指導する。

7.5 被験者ごとの中止基準

以下のいずれかに該当する場合、研究責任者又は研究者の判断により本研究の参加を中止する。研究責任者又は研究者は、研究中止の主な理由を以下の分類に従って症例報告書に記録する。

1. Pretreatment Event 又は有害事象

研究の継続により被験者の健康への容認できないリスクを避けるため、早期の中止が必要と考えられる Pretreatment event 又は有害事象がが発現した場合、若しくは被験者が Pretreatment Event 又は有害事象により研究の継続を望まない場合。

2. 臨床研究実施計画書からの重大な逸脱

無作為化後に、被験者が臨床研究実施計画書の組入れ基準を満たさないことが判明したこと又は臨床研究実施計画書を遵守しなかったことにより、研究を継続することにより被験者の健康に容認できないリスクを与える場合。

3. 追跡不能

被験者が来院せず、連絡も取れなかった場合。この場合は連絡を取ろうとしたことを原資料に記録する。

4. 自発的な中止

被験者が、研究への参加中止を希望する場合。

5. 研究全体の中止

研究依頼者、倫理審査委員会又は規制当局が研究を中止するよう決定した場合。

6. 妊娠

女性被験者の妊娠が判明した場合。

7. その他

その他の理由により研究責任者又は研究者が中止を判断した場合。その詳細を症例報告書に記載する。

7.6 被験者の研究中止の手続き

研究責任者又は研究者は、被験者が 7.5 項に記載する基準に抵触した場合には、当該被験者の研究への参加を中止する。また、被験者は研究実施中のいつでもその理由を説明することなく研究への参加を中止することができる。研究責任者又は研究者は、被験者の研究参加が中止される場合には、その主な理由を症例報告書に記録する。その際は、中止時に実施すべきすべての検査・観察・評価を可能な限り行う。

8.0 治療

8.1 被験薬又は対照薬による治療

8.1.1 投与量及び投与方法

アジルサルタン 20 mg 又はテルミサルタン 40 mg を 1 日 1 回、朝食前又は朝食後の午前中に服薬する。治療期開始時（Week0）の翌日より服薬を開始する。ただし、治療期の規定の来院時はアジルサルタン 20 mg 又はテルミサルタン 40 mg を服薬せずに来院し、所定の検査が終了してから服薬する。

表 8.a 投与量及び投与方法

群	投与量	投与経路	投与方法
アジルサルタン群	アジルサルタン 20 mg	経口投与	1 日 1 回、朝食前又は朝食後の午前中
テルミサルタン群	テルミサルタン 40 mg	経口投与	1 日 1 回、朝食前又は朝食後の午前中

8.1.2 過量投与

過量投与とは、判明している故意又は過失により被験薬又は対照薬が臨床研究実施計画書に規定される量を超えて投与されること又は被験者により服用されることと定義する。

過量投与に関する重要な安全性情報をデータベースに一貫して入力するため、研究責任者又は研究者は、過量投与のすべての事例（有害事象の有無に係わらず）を症例報告書の過量投与の頁に記録する。過量投与に伴う有害事象は、10.0 PRETREATMENT EVENT 及び有害事象の項に従って、症例報告書の有害事象の頁に記録する。

また、過量投与に伴う重篤な有害事象（SAE）については、10.2.2 重篤な有害事象の収集及び報告の項に記載された手順に従って報告する。

被験薬又は対照薬が過量投与された場合には、研究責任者又は研究者は症状に基づいて治療する。

8.2 経口血糖降下薬の治療

同意取得時に服薬していた経口血糖降下薬は使用可とするが、治療期間中に用法・用量は変更しない。

8.3 被験薬、対照薬及び経口血糖降下薬以外の治療

併用禁止薬（7.3 項参照）は使用不可とする。その他の治療は、日常診療下で行う。

8.4 割付表作成と保管

割付責任者が割付表を作成し、被験者の割付情報を管理する。割付の情報は安全な場所に保管され、権限を持つ者以外が入手できないようにし、本研究からの独立性を担保する。

8.5 割付及び投与手順

本研究では、降圧薬の有効性評価に影響を与える可能性があると考えられる因子について各群が均一に割付けられることを目的として層別割付を採用し、治療期開始時（Week0）に、治療

期開始時点の HOMA-R（2.5 未満、2.5 以上）、服薬中の経口血糖降下薬（ビグアナイド薬の有無）を考慮して登録センターにて実施する。登録センターでは、web 症例登録システムを用いて割付を実施する。また、登録センターでは、層別割付を行うために、割付表及び最新の割付済み例数の各群の内訳を用いるが、当該割付表及び各群の割付済み例数の情報は本研究の終了まで研究依頼者、研究調整医師、研究責任者及び研究者に開示しない。

研究責任者又はその指名する者は、被験者の割付を行うために web 症例登録システムにアクセスする。その際、研究責任者又はその指名する者は、被験者コードの他割付に必要な情報を通知する。その後、web 症例登録システムによって、各被験者に投与すべき降圧薬が通知される。研究責任者又は研究者は、通知に従って被験薬又は対照薬を処方し、その薬剤情報を各被験者の症例報告書に記載する。

〔割付因子〕

以下を考慮して層別割付を行う。

① HOMA-R：「2.5 未満」、「2.5 以上」

② 服薬中のビグアナイド薬：「有」、「無」

9.0 研究実施計画

9.1 研究実施手順

研究責任者又は研究者は、以下の手順に従ってデータを収集する。被験者の検査・観察・評価は、原則として同一の研究責任者又は研究者が行う。研究スケジュールは付録 A に示す。

9.1.1 同意取得

同意取得の方法については、15.3 項に示す。

臨床研究機関において本研究の実施が許可された以降、選択基準に合致し、除外基準に抵触しない被験者に対して、同意・説明文書を用いて説明し、文書同意が得られた順に電子症例報告書システムに登録し、観察を開始する。本研究への組み入れのために服薬中の降圧薬を休薬する場合には、本研究への参加の同意を取得した後に実施する。被験者登録は、臨床研究機関での予定被験者数に達した時点で終了する。また、研究全体の無作為割付実施例数が計画被験者数（各群 20 例、計 40 例）に達すると考えられた時点で登録を終了する。

被験者の個人情報を保護するために被験者コードリストを作成し、同意取得した各被験者に被験者識別コードを付与し、匿名化する。この被験者識別コードは、研究期間を通して使用し、変更しない。

9.1.2 人口統計学的データ、既往歴及び前治療薬

(1) 人口統計学的データ

人口統計学的データとして、生年月日、性別、喫煙歴、飲酒歴、高血圧症及び糖尿病の発症（又は診断）時期を調査する。

(2) 既往歴

既往歴として、同意取得前に消失又は終了した下記①及び②に該当する症状又は疾患を調査する。症状又は疾患が継続している場合には、既往歴とはせずに合併症とする。（9.1.6 項参照）

① 下記の循環器関連疾患

・心疾患：心筋梗塞、冠動脈・血行再建術

・脳血管障害：脳梗塞・脳出血、一過性脳虚血発作

・進行した高血圧性網膜症（出血又は滲出・乳頭浮腫）

② その他、研究責任者又は研究者が臨床的に重要と判断した症状又は疾患

(3) 前治療薬

前治療薬として、同意取得前 3 ヵ月以内に使用を終了したすべての降圧薬及び糖尿病治療薬について、薬剤名、投与終了日を調査する。

9.1.3 診察

診察を行い、治療期開始後の診察結果について、治療期開始前の診察結果と比較して臨床的に問題と考えられる異常の有無を評価する。

9.1.4 体重、身長及び BMI

体重及び身長を測定する。また、BMI は以下の数式を用いて算出する。

評価指数： BMI＝体重（kg）／（身長（m））^{2}

身長は整数のみのセンチメートルを単位として測定し、体重は小数点第 1 位までのキログラムを単位として測定する。BMI の結果は小数点第 1 位まで示す。また、身長は治療期開始時の測定値を用いる。

9.1.5 併用薬

併用薬とは、同意取得後から治療期終了までの間に使用したすべての薬剤のことである。被験者の来院の度に、同意取得から治療期終了までの間に使用した割付けられた降圧薬以外の薬剤について、使用状況（薬剤名、投与経路等）を調査する。また、同意取得時にビグアナイド薬服薬の有無を確認する。

9.1.6 合併症

合併症とは、同意取得時に有する症状又は疾患のことをいう。治療期開始時の検査及び診察で認められた臨床的に問題となる臨床検査値、心電図所見、診察所見の異常は研究責任者又は研究者の判断により合併症とする。

合併症として、下記①～③に該当する症状又は疾患を調査する。

① 脂質異常症

② 下記の循環器関連疾患

・安定した心疾患（心筋梗塞等）

・安定した脳血管障害（脳梗塞・脳出血等）

③ その他、研究責任者又は研究者が臨床的に重要と判断した症状又は疾患

9.1.7 バイタルサイン

座位収縮期・拡張期血圧、座位脈拍数を測定する。

座位血圧は 5 分間以上、座位の姿勢で安静を保った後に、1～2 分間隔で 2 回以上繰り返し測定する。連続した 2 回の測定値が安定した値（測定値の差が収縮期血圧は 10 mmHg 未満、拡張期血圧は 5mmHg 未満）を示した時点で測定をやめ、最後の連続した 2 回の測定値を記録する。

血圧測定前 30 分以内のカフェイン含有物の摂取及び喫煙は禁止する。また、血圧測定は右腕（何らかの理由により右腕での測定が困難な場合は左腕）で行い、研究期間を通じて変更しない。

治療期 2 週以降の血圧測定は、降圧薬を当日の朝に未服薬かつ前日に服薬の状態で午前中に開始する。

9.1.8 家庭血圧

家庭血圧は、起床時及び就寝前の、座位収縮期血圧及び座位拡張期血圧、座位脈拍数を測定する。

被験者への同意説明時に、本研究において家庭血圧を測定することを説明する。必ず被験者本人の血圧値を測定するよう被験者に指導する。

同意取得時に被験者へ家庭血圧計を提供し、実施手順について十分に説明する。治療期開始時の就寝前（可能な場合には、治療期開始の 7 日前の起床時）から最終来院日の起床時まで、毎日、起床時及び就寝前に 2 回ずつ測定するよう被験者に指導する。測定は、毎回の測定機会において、2 分間以上、座位の姿勢で安静を保った後に、30 秒以上の間隔で 2 回繰り返し測定する。

詳細については、別途定める家庭血圧測定に関する手順書を参照する。

9.1.9 臨床検査

臨床検査を観察スケジュール（付録 A）に従って実施する。空腹時（10 時間以上の絶食下）にて検体を採取する。

表 9.a 臨床検査

血液検査		尿検査
BUN<br>血清クレアチニン<br>総コレステロール<br>HDL<br>LDL（計算法）<br>空腹時トリグリセリド	HbA1c（NGSP 値）<br>1,5-AG<br>空腹時血糖<br>空腹時インスリン<br>高分子アディポネクチン<br>血漿アルドステロン*<br>血漿レニン活性*<br>高感度 CRP	尿中アルブミン**<br>尿中クレアチニン**<br>尿中 Na

*血漿アルドステロン、血漿レニン活性：座位で 15 分間以上安静の後に検体を採取する。

**随時尿にて尿中アルブミンを同時に測定した尿中クレアチニン濃度で補正し、尿中アルブミン／クレアチニン比を算出する。尿中アルブミン／クレアチニン比（mg/g・Cr）＝尿中アルブミン（mg/L）×100／尿中クレアチニン（mg/dL）

臨床研究機関において、上記の臨床検査を実施する。研究責任者又は研究者は、報告された臨床検査結果を評価する。

9.1.10 妊娠

研究責任者又は研究者は、女性被験者の同意のもとに、女性被験者が妊娠したときに研究に参加していたこと及び被験薬の詳細を、女性被験者の主治医（産婦人科医）に報告する。

研究責任者又は研究者は、妊娠が報告されたすべての被験者について、被験者の同意のもとに、早産等の結果も含め出産まで追跡調査し、所定の追跡調査の様式を使用して研究依頼者に報告する。出生後の評価も実施する。

9.1.11　割付

すべての選択基準を満たし、除外基準のいずれにも抵触しない被験者のみを 8.5 項に従い無作為化する。

9.1.12　無作為化前に中止した症例の記録

同意書に署名又は記名・捺印し、無作為化前に中止した全被験者について、症例報告書を作成する。症例報告書には、以下の項目を記載する。

・同意取得日

・生年月日

・性別

・適格性

・Pretreatment Event の有無（ある場合はその内容）

・中止理由

無作為化前に中止した被験者の主な理由を次の分類で症例報告書に記録する。

・Pretreatment Event

・選択基準を満たさない又は除外基準に抵触

・臨床研究実施計画書からの重大な逸脱

・追跡不能

・自発的な中止

・研究全体の中止

・妊娠

・その他（理由を記載する）

無作為化前に中止した症例の被験者識別コードは再度使用しない。

9.2　被験者の服薬状況

研究責任者又は研究者は、被験者に対し来院ごとに、降圧薬（被験薬又は対照薬）及び併用する血糖降下薬の服薬状況を確認する。また、来院日当日の被験薬又は対照薬の服薬の有無を確認する。さらに、被験薬又は対照薬の服薬終了時点において、全体の服薬状況を 4 分類「きちんと服薬した（90%以上）、だいたい服薬した（70%以上）、半分は服薬した（50%以上）、半分も服薬しなかった（50%未満）」にて確認する。

研究期間を通して、被験者に対して服薬指導を行う。前回の来院後、被験薬又は対照薬及び併用する血糖降下薬において、規定された服薬量の 50%未満等、被験者の服薬不遵守が判明し、改善が認められない場合には、状況に応じて研究を中止する。

9.3 検査・観察項目の実施時期

すべての検査・観察・評価のスケジュールを付録 A に示す。研究責任者又は研究者は、以下に記載する検査・観察・評価を規定された時点に実施する。

9.3.1 治療期開始前

同意取得後、研究への組み入れのための診察・検査を実施する。7.1 項の選択基準及び 7.2 項の除外基準に基づき被験者の適格性を確認する。

また、同意取得時に降圧薬を服薬している被験者を組み入れるときには、同意取得後に降圧薬を休薬する。

9.3.2 治療期

治療期開始前における検査・観察・評価項目の結果、適格性が認められた被験者を 8.5 項に従い、無作為化する。

治療期に実施する検査・観察・評価項目を以下に示す。

9.3.2.1 治療期開始時（Week0）

・選択基準／除外基準

・人口統計学的データ

・既往歴

・前治療薬（降圧薬、糖尿病治療薬）

・診察

・身長、体重、BMI

・合併症

・併用薬

・バイタルサイン（座位収縮期・拡張期血圧、脈拍数）

・家庭血圧（座位収縮期・拡張期血圧、脈拍数）

・臨床検査

・Pretreatment Event

9.3.2.2 治療期（Week2）

研究責任者及び研究者が必要と判断した場合に実施する。（例えば、軽度の腎疾患、肝疾患等を合併している又は著しい血圧低下・上昇が認められる等、アジルサルタン 20mg 又はテルミサルタン 40mg にて治療することの安全性を確認する必要がある場合）

・診察

・併用薬

・バイタルサイン（座位収縮期・拡張期血圧、脈拍数）

・服薬状況（被験薬又は対照薬）

・有害事象

9.3.2.3 治療期（Week4、Week8）

・診察

・併用薬

・バイタルサイン（座位収縮期・拡張期血圧、脈拍数）

・家庭血圧（座位収縮期・拡張期血圧、脈拍数）

・服薬状況（被験薬又は対照薬）

・有害事象

9.3.2.4 治療期終了時（Week12）

・診察

・体重、BMI

・併用薬

・バイタルサイン（座位収縮期・拡張期血圧、脈拍数）

・家庭血圧（座位収縮期・拡張期血圧、脈拍数）

・臨床検査

・服薬状況（被験薬又は対照薬）

・有害事象

・終了状態

9.3.2.5 治療期中止時

・診察

・体重、BMI

・併用薬

・バイタルサイン（座位収縮期・拡張期血圧、脈拍数）

・家庭血圧（座位収縮期・拡張期血圧、脈拍数）

・臨床検査

・服薬状況（被験薬又は対照薬）

・有害事象

・中止理由

9.4 生体試料の使用方法と保存、廃棄

研究責任者は、被験者の個人に関する情報の保護に必要な管理体制を整備し、試料の採取、保存、廃棄に関して、当該臨床研究機関の規定に従い実施する。

10.0 PRETREATMENT EVENT 及び有害事象

10.1 定義

10.1.1 Pretreatment Event

Pretreatment Event（PTE）とは、同意取得後、被験薬又は対照薬が投与されない期間において被験者に生じたあらゆる好ましくない医療上のできごと。必ずしも研究の手順との因果関係が明らかなもののみを示すものではない。

10.1.2 有害事象

有害事象とは、医薬品が投与された被験者に生じたあらゆる好ましくない医療上のできごと。必ずしも当該医薬品の投与との因果関係が明らかなもののみを示すものではない。

つまり有害事象とは、医薬品が投与された際に起こる、あらゆる好ましくない、あるいは意図しない徴候（臨床検査値の臨床的に問題のある異常を含む）、症状又は病気のことであり、当該医薬品との因果関係の有無は問わない。

10.1.3 Pretreatment Event 及び有害事象に関して考慮すべき事項

一般的に好ましくない所見には以下を含む：

- 新たに診断された疾患又は既存症状の予期せぬ悪化を示すもの（既存症状の間欠的事象は PTE 又は有害事象とはみなさない）
- 処置又は診療行為を必要とするもの
- 侵襲性の診断的処置が必要なもの
- 使用中の治療薬又は併用薬の中止又は投与量の変更を必要とするもの
- 研究責任者が好ましくないとみなしたもの

診断名と徴候・症状：

PTE 又は有害事象（以下、「有害事象等」）は診断名で記載する。随伴する徴候（臨床検査値の異常、心電図の異常所見を含む）及び症状は有害事象等としない。診断名がつかない有害事象等の場合は、徴候や症状を有害事象等とする。

臨床検査値及び心電図所見：

臨床検査値異常や心電図所見については、推移より臨床的に問題があると研究責任者又は研究者が判断した場合、有害事象等とする（すなわち、何らかの処置又は診療行為を必要とする場合、若しくは研究責任者又は研究者が、被験者の正常な生理的変動値域を超えた変化であると判断した場合）。

再検査及び異常値の継続的なモニタリングは診療行為とはみなさない。さらに、検証、評価、異常値のモニタリングの目的で非侵襲性検査を繰返し実施すること又は追加で実施することも診療行為とはみなさない。

ただし、臨床検査値異常や心電図所見が、有害事象等としての診断名がつく疾患に随伴する場合（例：腎機能障害によるクレアチニン増加等）は、その診断名を有害事象等とする。

既存症状（同意取得前から存在する疾患、症状）：

同意取得前から存在する疾患、症状については合併症とし、有害事象等としない。また、同意取得後の最初の検査・観察（例：臨床検査、心電図、X 線検査等）によって認められた異常所見は、研究手順に関連する場合を除き PTE としない。ただし、最初の検査・観察の手順によって生じた異常所見（例：採血時の内出血等）は PTE とし、症例報告書に記録する。合併症が悪化した場合は、その悪化を有害事象等とし、研究責任者又は研究者は、その有害事象等が合併症の悪化であることを症例報告書に記載する（例：「糖尿病の悪化」等）。

被験者が一過性の既存症状（例：喘息、てんかん）を有する場合にその症状の発現頻度が増加、あるいは重篤又は高度となる場合には、有害事象等として記録する。また、被験者が慢性的な疾患（例：白内障、関節リウマチ）を有する場合、予想以上に症状の悪化が見られる場合には、有害事象等として記録する。研究責任者又は研究者は、報告する有害事象名がベースラインからの症状の変化（例：～の悪化）であることがわかるように記録する。

有害事象等の悪化：

被験薬又は対照薬投与後に PTE が悪化した場合、あるいは PTE によって二次的に徴候・症状が発現した場合には、新たな有害事象とし、症例報告書に記録する。研究責任者又は研究者は、報告する有害事象名が症状の変化（例：～の悪化）であることがわかるように記録する。

有害事象又は重篤な PTE の重症度の変化：

被験者の有害事象又は重篤な PTE の重症度に変化がある場合には、重症度が最大となったときの一回を記録する。

あらかじめ計画されていた手術又は処置：

同意取得前にあらかじめ計画されていた手術又は処置は、有害事象等としない。ただし、その手術又は処置を緊急的に実施する必要が生じるような既存症状の悪化が生じた場合には、その状態や事象を有害事象等とする。あらかじめ計画されていた手術による合併症は、有害事象等として報告する。

急を要しない手術又は処置：

被験者の病状に変化を与えない急を要しない手術又は処置（美容整形等）は有害事象等としない。急を要しない手術による合併症は、有害事象等として報告する。

臨床的な反応の不足（有効性の欠如）：

臨床的な反応、有効性、薬理作用の不足は有害事象等としない。研究責任者又は研究者は、既存症状の悪化と有効性の欠如を区別して取り扱う。

過量投与：

事象の発現を伴わない過量投与は有害事象としないが、事象が発現した場合には、有害事象として症例報告書の有害事象頁に記録する。

10.1.4　重篤な有害事象

重篤な有害事象とは、医薬品が投与された（投与量にかかわらない）際に生じたあらゆる好ましくない医療上のできごとのうち、以下のものをいう。なお、PTE が下記重篤の基準に該当する場合には重篤な有害事象と同様の手順で報告する（10.2.2 項及び 10.2.3 項参照）。

1. 死に至るもの
2. 生命を脅かすもの*
3. 入院又は入院期間の延長が必要となるもの
4. 永続的又は顕著な障害若しくは機能不全に陥るもの
5. 先天異常をきたすもの
6. 1～5 に準じて医学的に重大な状態　即座に生命を脅かしたり、死や入院には至らなくとも、被験者を危険にさらしたり、上記 1～5 のような結果に至らぬように処置や治療を必要とするような重大な医学的事象。なお、Takeda Medically Significant AE List（表 10.a）に記載されているものは本項に含まれる。

*「生命を脅かす」とは、その事象が起こった際に被験者が死の危険にさらされていたという意味であり、その事象がもっと重症なものであったなら死に至っていたかもしれないという仮定的な意味ではない。

表 10.a　Takeda Medically Significant AE List

急性呼吸不全／急性呼吸窮迫症候群（ARDS）	肝壊死
トルサード　ド　ポアン／心室細動／心室性頻脈	急性肝不全
悪性高血圧	アナフィラキシーショック
痙攣性発作（痙攣、てんかんを含む）	急性腎不全
無顆粒球症	肺高血圧症
再生不良性貧血	肺線維症（間質性肺炎を含む）
中毒性表皮壊死融解症／皮膚粘膜眼症候群（スティーブンス・ジョンソン症候群）	悪性症候群／悪性高熱
	自然流産／死産及び胎児死
	薬剤を介した感染症の伝播又は伝播の疑い
	エンドトキシンショック又はその疑い

10.1.5　有害事象等の程度

有害事象等の程度については次のように分類・定義する。

軽　度	一過性で容易に耐えられるもの
中等度	通常の活動に支障をきたす程度のもの
高　度	通常の活動を不可能にする程度のもの

10.1.6　有害事象の因果関係

有害事象の被験薬又は対照薬との因果関係については次のように分類・定義する。

関連あり	時間的に明白な相関関係（投与中止後の経過を含む）がある。又は、原疾患、合併症、併用薬、併用処置等の他要因も推定されるが、当該被験薬、対照薬による可能性も考えられる有害事象。
関連なし	当該被験薬、対照薬との時間的に明白な相関関係がない。又は、原疾患、合併症、併用薬、併用処置等の他要因によると十分に考えられる有害事象。

10.1.7　研究手順との因果関係

有害事象等について、研究責任者又は研究者が、研究の手順に起因すると判断する場合には因果関係を「有」とし、研究の手順に起因しないと判断する場合には「無」とする。

10.1.8　発現日

有害事象等の発現日は、以下の基準で判断する。

有害事象等	発現日
徴候、症状、疾患（診断名）の場合	被験者又は研究責任者又は研究者が最初に有害事象等の徴候、症状に気付いた日を記載する。
無症候性の疾患の場合	診断のために検査が実施され、診断が確定した日を記載する。<br>検査所見から陳旧性の所見が見られる場合や、発生時期がおおよそ推定できる場合でも、診断が確定した日を記載する。
合併症又は PTE の悪化の場合	被験者又は研究責任者又は研究者が最初に疾患、症状の悪化に気付いた日を記載する。
同意取得後の最初の検査で正常であったものが、その後の検査で異常となった場合（PTE の場合）<br>被験薬又は対照薬投与開始後の検査で異常となった場合（有害事象の場合）	臨床的に問題があると判断される検査値異常が認められた検査日を記載する。
同意取得後の最初の検査で異常が見られ、その後の検査で悪化した場合（PTE の場合）<br>被験薬又は対照薬投与開始後の検査で異常が見られ、その後の検査で悪化した場合（有害事象の場合）	検査値の推移より医学的判断で明らかな上昇、低下、増加、減少が認められた検査日を記載する。

10.1.9　消失日

有害事象等が、回復した日（回復したが後遺症ありを含む）とする。当該有害事象等で死亡した場合には、死亡日とする。研究終了時に回復が確認できない場合には、継続中と判定する。

10.1.10 被験薬又は対照薬に関する処置

被験薬又は対照薬に関する処置については次のように分類・定義する。

投与中止	当該有害事象に対する処置として被験薬又は対照薬の投与が中止された場合（被験者の自己判断による中止も含む）を「投与中止」とする。
投与量変更せず	当該有害事象発現後も被験薬又は対照薬の投与量を変更しなかった場合を「投与量変更せず」とする。<br>当該有害事象以外の有害事象により被験薬又は対照薬投与が中止・減量・増量された場合は「投与量変更せず」とする。<br>また、被験者の不注意等の有害事象への対処以外の理由によって服薬を止めたり、減量する等した場合には「投与量変更せず」とする。
不明	被験者と連絡が取れず、発現日以降の経過が把握できない場合等。
該当せず	当該有害事象の発現を認めた時点で、被験薬又は対照薬の投与が既に終了又は中止されていた場合等。
減量	当該有害事象に対する処置として被験薬又は対照薬の投与量が減量された場合（被験者の自己判断による減量も含む）を、「減量」とする。
増量	当該有害事象に対する処置として被験薬又は対照薬の投与量が増量された場合（被験者の自己都合による増量も含む）を、「増量」とする。
休薬	当該有害事象に対する処置として被験薬又は対照薬の投与量が一時中止（中断）され、後日、投与が再開された場合（被験者の自己都合による一時中止も含む）を「休薬」とする。

10.1.11 転帰

有害事象等の転帰については次のように分類する。

区分	判断基準
回復	・症状、所見の消失又は回復<br>・検査値の正常化あるいは投与前の検査値（有害事象の場合）若しくは同意取得後最初の検査値（PTE の場合）への回復
軽快	・程度が 1 段階以上軽減<br>・症状、所見がほぼ消失<br>・検査値の改善が認められたが、正常化又は投与前の検査値（有害事象の場合）若しくは同意取得後最初の検査値（PTE の場合）に回復していない<br>・死亡例で、当該有害事象等が直接の死因でない場合で、当該有害事象等が軽快のまま死亡（この場合は死亡日の記載は不要）

未回復	・症状、所見や検査値に変化がない<br>・観察できた期間の最後の日の症状、所見や検査値が発現時の程度より悪化<br>・不可逆性の先天異常<br>・死亡例で、当該有害事象等が直接の死因でない場合で、当該有害事象等が未回復のまま死亡（この場合は死亡日の記載は不要）
回復したが後遺症あり	・日常生活に支障をきたす程度の機能不全が起きた
死亡	・死亡と当該有害事象等との間に直接の関連性が認められた<br>「直接の関連性が認められた」とは、当該有害事象等が死亡の原因になった、又は当該有害事象等が明らかに死亡に寄与したことをさす<br>・同一症例で見られた直接の死因ではないと判定（判断、推定）される有害事象等の転帰については、死亡としない<br>・転帰が、死亡の場合には、死亡日を記載する
不明	・発現日以降の経過が、転院、転居等により臨床研究実施計画書に記載されている追跡が不可能となった

10.2 手順

10.2.1　有害事象等の収集及び報告

10.2.1.1　有害事象等の収集期間と対象

有害事象等の収集期間及び対象は、以下の通りとする。

・同意取得時から被験薬又は対照薬の投与開始までに発現したすべての PTE

・被験薬又は対照薬の投与開始から治療期終了時（又は中止時）までに発現したすべての有害事象

10.2.1.2　有害事象等の報告

研究責任者又は研究者は、被験者来院の際、自覚症状の発現の有無を確認する。「前回の来院以降どうでしたか」等の質問を行い、来院時以外に発現した有害事象等を被験者から聴取する。

PTE が重篤の基準に該当する場合には、研究責任者又は研究者は、症状が消失するまで、臨床的に問題がある検査値の異常が同意取得後の最初の検査値に回復するまで、そうでない場合（永続的・不可逆的な PTE）には、観察された変化について十分な説明がつくまで、有害事象等が発現したすべての被験者を追跡調査する。PTE が重篤の基準に該当しない場合には、研究手順との関連性に係わらず、研究実施計画書上の追跡調査は必要としない。

有害事象等については、研究責任者又は研究者は、被験薬又は対照薬との関連性に係わらず、症状が消失するまで、臨床的に問題がある検査値の異常が被験薬又は対照薬の開始時の検査値に回復するまで、そうでない場合（永続的・不可逆的な有害事象等）は観察された変化について十分な説明がつくまで、有害事象等が発現したすべての被験者を追跡調査する。

すべての有害事象等を症例報告書に記載する。有害事象等の名称、発現日、消失日、程度、研究手順との因果関係（関連なし、関連あり）、被験薬又は対照薬との因果関係（関連なし、関連あり）、被験薬又は対照薬に関する処置、転帰及び重篤性について記録する。

有害事象及び重篤の基準に該当する Pretreatment Event の追跡期間は、有害事象等が回復するまで、あるいは研究責任者又は研究者がこれ以上の追跡は不要と判断するまでの期間とする。

研究依頼者からの要請がある場合には、研究責任者又は研究者は、指定する期間内に必要な追加情報、データを確認し、電子症例報告書システムへの入力を完了する。

10.2.2　重篤な有害事象の収集及び報告

10.2.2.1　重篤な有害事象の収集期間と対象

重篤な有害事象の収集期間及び対象は、以下の通りとする。

・被験薬又は対照薬の投与開始から治療期終了時（又は中止時）までに発現したすべての重篤な有害事象

・治療期終了時（又は中止時）以降に発現した本研究との因果関係が「関連あり」と判定された重篤な有害事象

10.2.2.2　重篤な有害事象等の報告

重篤な有害事象が収集期間内に発現した場合には、以下の手順に従って報告する。また、PTE が 10.1.4 項に記載の重篤の基準に該当する場合には、重篤な有害事象と同様の手順で報告する。

研究責任者又は研究者は、重篤な有害事象の発現時又は被験者等からの事象発現の通知後、研究依頼者の 1 就業日以内に、当該臨床研究機関の長及び研究依頼者（連絡先は別紙参照）に報告書を提出する。さらに、研究責任者は 10 暦日以内に正式な詳細報告書を提出する。

なお、報告する内容は以下の項目を必須とし、その他の項目についても可能な限り連絡する。

- 研究課題名
- 被験者識別コード
- 臨床研究機関名
- 研究責任者名
- 重篤な有害事象名、経過（発現日、重篤と判断した理由、使用中の治療薬名、治療薬との因果関係、経過、転帰）
- 重篤な有害事象に対する措置（新規登録の中断、同意・説明文書の改訂、他の被験者への再同意）

10.2.3　重篤な有害事象の臨床研究機関への周知

臨床研究機関の長は、研究責任者から重篤な有害事象の報告を受けた場合には、倫理審査委員会に意見を聴き、研究責任者からの報告に以下の項目を追加して、研究依頼者を通じて臨

床研究を実施する臨床研究機関に周知する。なお、PTE が 10.1.4 項に記載の重篤の基準に該当する場合には、重篤な有害事象と同様の手順で周知する。

- 倫理審査委員会における審査日、審査内容の概要、結果、必要な措置等

10.3 重篤な有害事象の追跡調査

研究責任者又は研究者は、すべての重篤な有害事象が回復するまで、又は最終の転帰が確定するまで追跡調査する。なお、PTE が 10.1.4 項に記載の重篤の基準に該当する場合には、重篤な有害事象と同様の手順で追跡調査する。

研究責任者又は研究者は、転帰の変更等、重篤な有害事象の報告内容に変更が生じた場合、臨床研究機関の長及び研究依頼者に変更内容を記した報告書を提出する。研究依頼者又は倫理審査委員会から要求された場合には、臨床研究機関の関連データ（例：心電図、臨床検査値、退院報告の要約、検死結果等）を提供する。

11.0　本研究のために設置された委員会

本研究では、いずれの委員会も設置しない。

12.0 データマネジメント及び記録の保存

データマネジメントに関する詳細な手順については、データマネジメント計画書に記載する。PTE、有害事象、既往歴及び合併症は MedDRA を用いて読み替える。薬剤については、WHO Drug を用いて読み替える。

12.1 症例報告書

研究責任者又は研究者は、同意を取得したすべての被験者について症例報告書を作成する。

研究依頼者又はその指名する者は、研究責任者、研究者及び研究協力者に電子症例報告書システム（electronic data capture「以下、EDC」）へのアクセス権を提供する。研究依頼者は、EDC の利用に際し、研究責任者、研究者及び研究協力者を対象にトレーニングを提供する。症例報告書は、EDC にデータを直接入力することにより作成する。症例報告書は日本語で作成する。

症例報告書の変更又は修正は、変更又は修正前後の情報、変更又は修正者、変更又は修正日及びその理由を記録した監査証跡として記録される。

研究責任者は症例報告書が正確かつ完全に作成されているかを確認し、症例報告書の該当頁に電子署名する。研究責任者は、EDC に入力された全データに関する正確性と信頼性について全責任を負う。

以下は症例報告書として EDC に直接入力されたデータである。（ただし、原資料に記載がある場合は除く）

- 研究責任者又は研究者のコメント
- 有害事象等の重篤度、程度、被験薬又は対照薬並びに研究手順との因果関係、転帰

研究依頼者又はその指名する者は、必要に応じて臨床研究機関を訪問し、症例報告書が正確かつ完全に作成されていることを確認する。研究依頼者又はその指名する者は、症例報告書の正確性を確保するため、研究に関連する被験者の医療記録及び院内記録を必要に応じて閲覧する。

作成が完了した症例報告書は研究依頼者の所有物であり、研究調整医師、研究責任者及び研究者は、その情報を研究依頼者の書面による許可なしに、規制当局を除く第三者に開示してはならない。

データベースを固定した後に、研究責任者又は研究者が症例報告書の入力内容を変更又は修正する場合には、研究依頼者から提供される症例報告書の変更及び修正記録（Data Clarification Form）を用いて行う。研究責任者は、症例報告書の変更及び修正記録が正確かつ完全に記載されているかを確認後、署名又は記名・捺印し、日付を記載する。

12.2 記録の保存

研究責任者及び臨床研究機関の長は、規制当局及び研究依頼者又はその指名する者による調査又は監査のため、本研究固有の文書を含む以下の資料を保管する。資料には、被験者コードリスト、医療記録、署名・日付入り同意書原本等がある。また、研究責任者及び臨床研究機関の長は、保管すべき必須文書を、研究の中止又は終了後 5 年が経過した日までの間保存しなければならない。ただし、研究依頼者がこれよりも長期間の保存を必要とする場合には、臨床研究機関の長は、保存期間及び保存方法について研究依頼者と協議する。

さらに、研究責任者及び臨床研究機関の長は、保存すべき必須文書についてその保存の必要がなくなった旨研究依頼者から通知を受けるまで保存する。

13.0 統計手法

13.1 統計及び解析計画

本研究に関わる統計解析の主な内容を以下に示す。

解析担当者は、データ固定前に統計解析計画書を作成し、最終化する。統計解析計画書には、すべての研究の目的に対処するため、評価項目の定義及び解析方法の詳細を記載する。

13.1.1 解析対象集団

本研究では、解析対象集団として「最大の解析対象集団」及び「安全性データの解析対象集団」の 2 種類の解析対象集団を設ける。

「最大の解析対象集団」は、以下の基準を満たす登録被験者の集団として定義する。

・ 無作為化され、本研究中に被験薬又は対照薬を少なくとも 1 回以上投与された被験者

「安全性データの解析対象集団」は、以下の基準を満たす登録被験者の集団として定義する。

・ 本研究中に被験薬又は対照薬を少なくとも 1 回以上投与された被験者

13.1.1.1 有効性の解析対象集団

有効性評価項目について、「最大の解析対象集団」を対象として解析を行う。

13.1.1.2 安全性の解析対象集団

安全性評価項目について、「安全性データの解析対象集団」を対象として解析を行う。

13.1.2 人口統計学的及び他の基準値の特性の解析

「無作為化された全例」を対象として、主要な被験者背景項目を投与群別及び投与群を併合して集計する。

13.1.3 有効性の解析

13.1.3.1 主要評価項目

〔主要評価項目〕

・インスリン抵抗性指数（HOMA-R*）

治療期終了時点における治療期開始時点からの変化量

*：HOMA-R=空腹時インスリン値（μU/mL）×空腹時血糖値（mg/dL）/405

〔解析方法〕

1) 主解析

「最大の解析対象集団」を対象として、主要評価項目について、投与群別に要約統計量を算出し、平均値の投与群間差（アジルサルタン群－テルミサルタン群）の点推定値及びその両側 95%信頼区間を算出する。

2) 副解析

「最大の解析対象集団」を対象として、主要評価項目について、治療期開始時点のHOMA-R（2.5 未満、2.5 以上）、服薬中のビグアナイド薬（有、無）を層別因子とした投与群別の要約統計量を算出する。

13.1.3.2 副次評価項目

「最大の解析対象集団」を対象として、以下の項目について、投与群別に要約統計量を算出する。

・空腹時血糖値の変化量

治療期終了時点における治療期開始時点からの変化量

・空腹時インスリン値の変化量

治療期終了時点における治療期開始時点からの変化量

・HbA1c の変化量

治療期終了時点における治療期開始時点からの変化量

・HOMA-β*の変化量

治療期終了時点における治療期開始時点からの変化量

*：HOMA-β=空腹時インスリン値（μU/mL）×360/｛空腹時血糖値（mg/dL）-63｝

・1,5-AG の変化量

治療期終了時点における治療期開始時点からの変化量

13.1.3.3 その他の評価項目

「最大の解析対象集団」を対象として、以下の項目について、投与群別に要約統計量を算出する。

・診察時血圧値の変化量

治療期終了時点における治療期開始時点からの変化量

・家庭血圧計を用いた早朝血圧値の測定値

治療期終了時点における測定値

・BUN 値の変化量

治療期終了時点における治療期開始時点からの変化量

・血清クレアチニン値の変化量

治療期終了時点における治療期開始時点からの変化量

・総コレステロール値の変化率

治療期終了時点における治療期開始時点からの変化率

・HDL 値の変化率

治療期終了時点における治療期開始時点からの変化率

・LDL 値の変化率

治療期終了時点における治療期開始時点からの変化率

・空腹時トリグリセリド値の変化率

治療期終了時点における治療期開始時点からの変化率

・高分子アディポネクチン値の変化量

治療期終了時点における治療期開始時点からの変化量

・血漿アルドステロンの変化量

治療期終了時点における治療期開始時点からの変化量

・血漿レニン活性の変化量

治療期終了時点における治療期開始時点からの変化量

・高感度 CRP の変化量

治療期終了時点における治療期開始時点からの変化量

・尿中アルブミン／クレアチニン比の変化量

治療期終了時点おける治療期開始時点からの変化量

尿中アルブミン／クレアチニン比（mg/g･Cr）＝尿中アルブミン（mg/L）×100／尿中クレアチニン（mg/dL）

・尿中 Na／クレアチニン比の変化量

治療期終了時点おける治療期開始時点からの変化量

尿中 Na／クレアチニン比（g/day）＝尿中 Na（mEq/L）×2.3／尿中クレアチニン（mg/dL）

13.1.3.4　有意水準、信頼係数

信頼係数：95%（両側推測）

13.1.4　安全性の解析

・有害事象

有害事象は MedDRA を使用して読み替える。以下に示した有害事象について、器官別大分類（System Organ Class; SOC）かつ基本語（Preferred Term; PT）別の頻度集計を投与群別に行う。

・全ての有害事象

・程度別の有害事象

・被験薬又は対照薬との因果関係が否定できない有害事象

・程度別の被験薬又は対照薬との因果関係が否定できない有害事象

・投与中止に至った有害事象

・重篤な有害事象

13.2 中間解析及び早期中止に関する基準

中間解析は予定していない。

13.3 計画被験者数の決定

無作為化された被験者数として

アジルサルタン 20 mg 群：20 例

テルミサルタン 40 mg 群：20 例

【計画被験者数の設定根拠】

アジルサルタン 20mg 及びテルミサルタン 40mg のインスリン抵抗性に対する影響について探索的に検討する被験者数として、実施可能性を考慮して設定した。本設定は、統計学的根拠に基づいたものではない。

14.0 品質管理及び品質保証

14.1 臨床研究機関へのモニタリング

研究依頼者又はその指名する者は、臨床研究実施計画書に記載されているすべての内容に従って研究が実施されているかを確認するため、臨床研究機関へのモニタリングを研究期間を通して定期的に行う。症例報告書に記載されているデータを確認するため、原資料と照合する。原資料とは元となる文書、データ及び記録のことをいう。研究責任者及び臨床研究機関の長は、研究依頼者又はその指名する者及び倫理審査委員会が原資料を閲覧できることを保証する。

研究依頼者又はその指名する者は、被験者コードリスト、医療記録、署名・日付入り同意書原本を含む記録を閲覧し、研究が臨床研究実施計画書を遵守して適切に行われていることを確認する。研究責任者、研究者及びその他研究に携わる者は、モニタリングのための臨床研究機関への訪問が行われる際は作業進行のために十分な時間を割いて、対応するよう努める。

モニタリングに関する詳細な手順については、モニタリング計画書に記載する。

14.2 臨床研究に関する倫理指針及び臨床研究実施計画書からの逸脱

研究責任者は、臨床研究に関する倫理指針及び臨床研究実施計画書から逸脱した行為をすべて記録する。研究責任者は、逸脱が判明した場合、速やかに臨床研究機関の長及び研究依頼者に通知する。必要に応じ、臨床研究実施計画書の改訂について研究依頼者と協議し、改訂する場合には、臨床研究機関の長に提出し、倫理審査委員会の承認を得る。

逸脱に関する詳細な規準については、逸脱規定書に記載する。

14.3 品質保証及び規制当局による調査

研究依頼者又はその指名する者は、必要に応じて臨床研究機関において監査を実施する。この場合、研究依頼者が指定した監査担当者は事前に臨床研究機関と連絡を取り、監査のための訪問を取決める。監査担当者は、臨床検査検体の採取場所及びその他研究期間中使用する場所への訪問を要求できる。また、本研究は、海外政府機関（例：米国食品医薬品局[FDA]、英国医薬品規制当局[MHRA]）を含む規制当局によって調査されることもある。臨床研究機関が規制当局より調査を要求する連絡を受けた場合、研究依頼者に速やかに通知する。研究責任者及び臨床研究機関の長は、監査担当者が原資料をすべて閲覧できることを保証する。

15.0 研究の倫理的な実施

本研究は、被験者個人に配慮し、本臨床研究実施計画書、ヘルシンキ宣言及び「臨床研究に関する倫理指針（厚生労働省 平成 20 年改正版）」を遵守して実施する。

15.1 利益相反

研究責任者は、本研究を実施するに先立ち、本研究実施に利益相反（以下、「COI」）の問題がないことについて、臨床研究機関の規定に従い適切に管理しなければならない [7)-11)]。

臨床研究機関は、倫理審査委員会が規定するすべての要件を遵守する。これには、COI 自己申告書、臨床研究実施計画書、同意・説明文書が含まれる。

15.2 倫理審査委員会等の承認

研究責任者は、本研究の実施又は継続にあたり倫理審査委員会の審査のために、本研究実施計画書の他に、同意・説明文書及びその他規制によって求められる文書を、臨床研究機関の長に提供する。臨床研究機関の長は、臨床研究計画が臨床研究に関する倫理指針に適合しているか否かその他臨床研究の適正な実施に関し必要な事項について、あらかじめ、倫理審査委員会に審査を行わせる。研究責任者は、本研究を実施又は継続する前に倫理審査委員会が承認し、臨床研究機関の長が許可した記録を入手する。

臨床研究機関は、倫理審査委員会が規定するすべての要件を遵守する。これには、臨床研究実施計画書の改訂、同意・説明文書の改訂、規制要件に従った安全性に関する報告、倫理審査委員会によって設定された間隔での研究の実施状況報告及び研究終了報告の提出等、倫理審査委員会への通知が含まれる。

15.3 同意・説明文書及び被験者の同意

同意・説明文書には、ヘルシンキ宣言、臨床研究に関する倫理指針及びすべての適用される法規制で求められている内容が、具体的に記載されている。同意・説明文書には、被験者の個人情報及び個人医療情報に関して、本研究における利用方法（国内外を問わず：第三者提供）、開示について記述されている。同意・説明文書には、研究の主旨、目的、起こりうる危険と利益が詳しく説明されている。また、同意・説明文書には研究参加に関する条件と被験者がいつでも理由を説明することなく、また治療にあたって不利益を受けることなく研究参加を中止することができる旨が記載されている。

研究責任者は、同意・説明文書の作成、内容、倫理審査委員会の承認に関して責任を負う。同意・説明文書は、使用する前に倫理審査委員会から承認を受ける。

同意・説明文書には、被験者が理解できる表現を使用する。研究責任者又は研究者は、同意・説明文書の内容を被験者に詳しく説明する責任がある。情報は可能な限り、口頭及び書面で、倫理審査委員会が適切であると認める方法で提供する。

研究責任者又は研究者は、被験者に（1）研究に関する問い合わせの機会及び（2）研究に参加するかどうかを決定するための十分な時間を与える。被験者が研究への参加を決定した場合、研究に参加する前に本人は、同意書に署名又は記名・捺印し、日付を記載する。研究責任者又は研究者は、黒又は青のボールペンを用いて通称ではなく、正式な氏名で署名又は記名・捺印するよう被験者に求める。研究責任者又は研究者も被験者が研究に参加する前に、同意書に署名又は記名・捺印し、日付の記載を行う。

研究責任者又は研究者は、署名又は記名・捺印された同意書の原本を保管する。研究責任者又は研究者は、被験者が同意書に署名又は記名・捺印した日付を被験者の医療記録に記録する。また、署名又は記名・捺印入りの同意書の写しを被験者に提供する。

研究責任者又は研究者は、同意・説明文書を改訂した場合（研究期間等の軽微な変更は除く）、初回同意取得時と同様の手順で、再度同意を取得し、同意書の原本を保管する。再同意を取得した日付を被験者の医療記録に記録し、改訂した同意書の写しを被験者に提供する。

15.4 被験者の個人情報等の保護

研究依頼者又はその指名する者は、被験者の個人情報等保護の原則を遵守する。本研究では、被験者識別コードにより本研究データベース及び研究関連文書と被験者の原データを関連付ける。被験者の特定及び被験者識別コードの正確性確認のため、すべての適用される法令及び規則の範囲内で、性別、生年月日等の限定的な被験者の情報を用いることができる。

臨床研究に関する倫理指針に従い、本研究実施計画書が遵守され、研究が実施されていることを確認するため、研究依頼者は、研究依頼者が指名する者、規制当局の代表者、監査担当者及び倫理審査委員会が、臨床検査成績、研究参加期間中の入退院記録、検死結果等の医療記録の原本（原データ又は資料）を閲覧することの許可を研究責任者に要請する。研究責任者又は研究者は、被験者の同意を取得する際、研究依頼者が指名する者及び規制当局の代表者による医療記録原本の閲覧について、被験者から承諾を得る（15.3 項参照）。

研究依頼者に原資料の写しを提供する場合、研究責任者又は研究者は、個人が特定できる情報（被験者氏名、住所、その他被験者の症例報告書に記載されない個人識別情報）を削除する。

15.5 被験者の利益及び不利益

15.5.1 被験者の利益

本研究に参加することにより、自身の高血圧症及び糖尿病の状態等について詳しく知ることができる。

15.5.2 被験者の不利益

本研究に参加することにより来院回数や検査回数が増加するため、日常診療に比べて被験者の負担が増えると考えられる。また、治療期開始前の一定期間は、降圧薬を服薬できず、治療期間中は、高血圧、糖尿病及び合併症の治療が制限される場合がある。

15.6 研究成績の帰属、アクセス権及び公表の手順

15.6.1 研究成績の帰属

本研究によって得られたすべてのデータ及び情報は、研究依頼者に帰属する。

15.6.2 データのアクセス権

本研究から得られたすべてのデータ及び情報に対するアクセス権は、研究依頼者が承認した者に与える。

15.6.3　研究成績の公表の手順

研究実施中及び研究終了後、研究依頼者又はその指名する者は、速やかにその成果をまとめ、医学雑誌及び学会等に発表する。

研究依頼者は、研究から得られたデータ及び情報のいずれも公表することができる。

研究責任者は、本研究により得られた情報を専門の学会等外部に公表する場合には、事前に文書により研究依頼者の承認を得るものとする。

15.7　臨床研究実施計画の事前登録及び研究成績の開示

15.7.1　臨床研究実施計画の事前登録

研究依頼者又はその指名する者は、臨床研究の情報を適時に公表することを保証し、適用される法令、規則及び指針に準拠するため、本研究の開始前に、本研究の実施及び計画の概要を臨床研究計画の内容が公開されているデータベースに登録する。

15.7.2　研究成績の開示

研究依頼者又はその指名する者は、結果に関係なく、事前登録した本研究の実施及び計画の概要を臨床研究計画の内容が公開されているデータベースに本研究の成績を掲示する。

15.8　健康被害に対する補償

本研究に参加している被験者は、健康被害が生じた場合には、被験者が参加している臨床研究機関に適用される規制に従って補償される。なお、本研究の実施に際し、研究依頼者は、被験者の健康被害の補償のため保険に加入する。

16.0 参考文献

1) 高血圧治療ガイドライン 2009（日本高血圧学会）.

2) 糖尿病治療ガイド 2012-2013（日本糖尿病学会）

3) Benson S. C. et al.: Identification of Telmisartan as a unique Angiotensin Ⅱ receptor antagonist with selective PPAR γ -modulating activity. Hypertension 2004; 43; 993-1002

4) Watanabe M. et al: Effects of Telmisartan on insulin resistance in Japanese type 2 diabetic patients. Inter Med. 2010; 49; 1843-1847

5) Iwai M. et al.: TAK-536, a new AT1 receptor blocker, improvese glucose intolerance and adipocyte differentiation. Am J Hypertension 2007; 20; 579-586

6) 平光和博ら: 糖尿病合併高血圧患者に対するアジルサルタン（アジルバ錠）の有用性. 血圧 2013; 20 (1); 94-99

7) 利益相反ワーキング・グループ報告書（文部科学省　平成 14 年 11 月 1 日）

8) 臨床研究の利益相反ポリシー策定に関するガイドライン（臨床研究の倫理と利益相反に関する検討班　平成 18 年 3 月）

9) 厚生労働科学研究における利益相反（Conflict of Interest:COI）の管理に関する指針（平成 20 年 3 月 31 日）

10) 医学研究の COI マネージメントに関するガイドライン（日本医学会　臨床部会利益相反委員会 平成 23 年 2 月）

11) 臨床研究の利益相反（COI）に関する共通指針（日本内科学会、日本肝臓学会、日本循環器学会、日本内分泌学会、日本糖尿病学会、日本呼吸器学会、日本血液学会、日本アレルギー学会、日本感染症学会　2011 年 8 月）

付録 A　研究スケジュール

				**治療期**				
時期	**Week**	**-**	**0**	**2[**]**	**4**	**8**	**12**	中止[***]
	**Day**	**-**	**0[*]**	**14**	**28**	**56**	**84**	**-**
許容範囲		**-**	**0**	**1～21**	**22～42**	**43～70**	**71～98**	**-**
VISIT 番号		**-**	**1**	**2**	**3**	**4**	**5**	**-**
同意取得		X(a)						
選択基準／除外基準		X(a)	X					
人口統計学的データ			X					
既往歴・前治療薬			X					
診察			X	(X)	X	X	X	X
身長			X					
体重・BMI			X				X	X
合併症			X					
併用薬			←		X			→
バイタルサイン<br>座位血圧・脈拍数			X	(X)	X	X	X	X
家庭血圧		(X)	←		X			→
臨床検査		X(b)(d)					X(b)	X(c)
被験薬又は対照薬の処方			X	(X)	X	X		
服薬状況				(X)	X	X	X	X
有害事象等の評価			←		X			→

X：検査・観察等を実施。
← X →：期間を通じて、検査・観察等を実施。
(a) 同意取得時に降圧薬を服薬している被験者を組み入れるときには、同意取得後に降圧薬を休薬する。
(b) 空腹時（10 時間以上の絶食下）にて測定する。
(c) 可能な場合、空腹時（10 時間以上の絶食下）にて測定する。
(d) 治療期開始前 1 週間以内に実施する。治療期開始時点で検査結果を確認することができ、可能な限り治療期開始時点に近い日に実施する。
[*]：被験薬又は対照薬は、Week0 の翌日より服薬を開始する。被験薬又は対照薬の投与開始日を Day1 とする。
[**]：研究責任者及び研究者が必要と判断した場合に実施。（例えば、軽度の腎疾患、肝疾患等を合併している又は著しい血圧低下・上昇が認められる等、アジルサルタン 20mg 又はテルミサルタン 40mg にて治療することの安全性を確認する必要がある場合）
[***]：可能な限り実施する。

付録 B　研究責任者の責務

1. 本研究実施計画書及び臨床研究に関する倫理指針を遵守し、被験者の人権、安全及び福祉に配慮して適切な研究を実施する。
2. 同意・説明文書を作成し、必要に応じて改訂する。
3. 臨床研究の契約内容を確認する。
4. 臨床研究機関の他の研究者及び研究に携わる者に臨床研究実施計画書、各人の業務について十分な情報を与え、指導及び監督する。
5. 臨床研究実施計画書に合致した被験者を選定し、文書により説明し、文書で同意を取得する。
6. 研究に関連する医療上のすべての判断に責任を負う。
7. 被験者の研究参加期間中及びその後を通じ、研究に関連した臨床上の問題となるすべての有害事象に対して、臨床研究機関の長とともに十分な医療が被験者に提供されることを保証する。
8. 重篤な有害事象等の緊急報告が必要な場合には、臨床研究機関の長に速やかに文書にて報告するとともに、研究依頼者を通じて臨床研究を実施する臨床研究機関の研究責任者に報告する。
9. 正確かつ完全な症例報告書を作成する。
10. 研究依頼者から臨床研究実施計画書等の改訂について提案があった場合には協議する。
11. 研究が終了した場合には、臨床研究機関の長に文書で通知する。

付録 C　改訂内容

以下に初版から第 2 版への変更を記載する。

変更項目	変更前	変更後	変更理由
Page 8<br>2.0　要約　計画被験者数	無作為化された被験者数として<br>アジルサルタン 20 mg 群：*50* 例<br>テルミサルタン 40 mg 群：*50* 例	無作為化された被験者数として<br>アジルサルタン 20 mg 群：**20** 例<br>テルミサルタン 40 mg 群：**20** 例	実施可能性を考慮して再考したため。
Page 19<br>6.1　研究デザイン	無作為化された被験者数として<br>アジルサルタン 20 mg 群：*50* 例<br>テルミサルタン 40 mg 群：*50* 例	無作為化された被験者数として<br>アジルサルタン 20 mg 群：**20** 例<br>テルミサルタン 40 mg 群：**20** 例	実施可能性を考慮して再考したため。
Page 30<br>9.1.1　同意取得	（略）<br>臨床研究機関において本研究の実施が許可された以降、選択基準に合致し、除外基準に抵触しない被験者に対して、同意・説明文書を用いて説明し、文書同意が得られた順に電子症例報告書システムに登録し、観察を開始する。本研究への組み入れのために服薬中の降圧薬を休薬する場合には、本研究への参加の同意を取得した後に実施する。<br>被験者登録は、臨床研究機関での予定被験者数に達した時点で終了する。また、研究全体の無作為割付実施例数が計画被験者数（各群 *50* 例、計 *100* 例）に達すると考えられた時点で登録を終了する。<br>（略）	（略）<br>臨床研究機関において本研究の実施が許可された以降、選択基準に合致し、除外基準に抵触しない被験者に対して、同意・説明文書を用いて説明し、文書同意が得られた順に電子症例報告書システムに登録し、観察を開始する。本研究への組み入れのために服薬中の降圧薬を休薬する場合には、本研究への参加の同意を取得した後に実施する。<br>被験者登録は、臨床研究機関での予定被験者数に達した時点で終了する。また、研究全体の無作為割付実施例数が計画被験者数（各群 **20** 例、計 **40** 例）に達すると考えられた時点で登録を終了する。<br>（略）	実施可能性を考慮して再考したため。
Page 50<br>13.3　計画被験者数の決定	無作為化された被験者数として<br>アジルサルタン 20 mg 群：*50* 例<br>テルミサルタン 40 mg 群：*50* 例<br>【計画被験者数の設定根拠】<br>アジルサルタン 20mg 及びテルミサルタン 40mg のインスリン抵抗性に対する影響について探索的に検討する被験者数として、実施可能性を考慮して設定した。本設定は、統計学的根拠に基づいたものではない。	無作為化された被験者数として<br>アジルサルタン 20 mg 群：**20** 例<br>テルミサルタン 40 mg 群：**20** 例<br>【計画被験者数の設定根拠】<br>アジルサルタン 20mg 及びテルミサルタン 40mg のインスリン抵抗性に対する影響について探索的に検討する被験者数として、実施可能性を考慮して設定した。本設定は、統計学的根拠に基づいたものではない。	実施可能性を考慮して再考したため。
